# Machine learning-assisted crystal engineering of a zeolite

**DOI:** 10.1038/s41467-023-38738-5

**Published:** 2023-05-31

**Authors:** Xinyu Li, He Han, Nikolaos Evangelou, Noah J. Wichrowski, Peng Lu, Wenqian Xu, Son-Jong Hwang, Wenyang Zhao, Chunshan Song, Xinwen Guo, Aditya Bhan, Ioannis G. Kevrekidis, Michael Tsapatsis

**Affiliations:** 1 Department of Chemical Engineering and Materials Science, University of Minnesota, 421 Washington Avenue SE, Minneapolis, MN 55455 USA; 2 State Key Laboratory of Fine Chemicals, PSU-DUT Joint Center for Energy Research, School of Chemical Engineering, Dalian University of Technology, Dalian, 116024 Liaoning Province China; 3 Department of Chemical and Biomolecular Engineering, Johns Hopkins University, 3400 North Charles Street, Baltimore, MD 21218 USA; 4 Department of Applied Mathematics and Statistics, Johns Hopkins University, 3400 North Charles Street, Baltimore, MD 21218 USA; 5 X-ray Science Division, Advanced Photon Source, Argonne National Laboratory, Lemont, IL 60439 USA; 6 Division of Chemistry and Chemical Engineering, California Institute of Technology, Pasadena, CA 91125 USA; 7 Applied Physics Laboratory, Johns Hopkins University, 11100 Johns Hopkins Road, Laurel, MD 20723 USA; 8 Institute for NanoBioTechnology, Johns Hopkins University, 3400 North Charles Street, Baltimore, MD 21218 USA

**Keywords:** Materials for energy and catalysis, Theory and computation

## Abstract

It is shown that Machine Learning (ML) algorithms can usefully capture the effect of crystallization composition and conditions (inputs) on key microstructural characteristics (outputs) of faujasite type zeolites (structure types FAU, EMT, and their intergrowths), which are widely used zeolite catalysts and adsorbents. The utility of ML (in particular, Geometric Harmonics) toward learning input-output relationships of interest is demonstrated, and a comparison with Neural Networks and Gaussian Process Regression, as alternative approaches, is provided. Through ML, synthesis conditions were identified to enhance the Si/Al ratio of high purity FAU zeolite to the hitherto highest level (i.e., Si/Al = 3.5) achieved via direct (not seeded), and organic structure-directing-agent-free synthesis from sodium aluminosilicate sols. The analysis of the ML algorithms’ results offers the insight that reduced Na_2_O content is key to formulating FAU materials with high Si/Al ratio. An acid catalyst prepared by partial ion exchange of the high-Si/Al-ratio FAU (Si/Al = 3.5) exhibits improved proton reactivity (as well as specific activity, per unit mass of catalyst) in propane cracking and dehydrogenation compared to the catalyst prepared from the previously reported highest Si/Al ratio (Si/Al = 2.8).

## Abstract (web summary)

Zeolites are porous aluminosilicate molecular sieves with uniform pores of molecular dimensions that have a wide range of applications. Here authors use machine learning to guide zeolite synthesis and predict the structure and properties of faujasite zeolites from synthesis conditions.

## Introduction

Zeolites are crystalline, porous aluminosilicate molecular sieves with uniform pores of molecular dimensions that are widely used in industrial applications such as catalysis, adsorption, membrane separation and ion exchange^1–6^. Their performance (sorption capacity, catalytic activity, selectivity, stability) depends on a hierarchy of microstructural characteristics. In addition to the framework topology (represented by a three-letter code)^7^, framework composition (i.e., the atomic Si/Al ratio of the tetrahedra in the framework) and extra-framework cation content, numerous other characteristics can be tuned to optimize the performance of a zeolite including crystallographic positions of Si and Al atoms^8^, crystallographic location of extra-framework cations^9^, crystal size and shape^10–12^, extent of crystallite aggregation comprising a zeolite particle^13,14^, the presence of meso-porosity^15,16^, the occurrence and frequency of intergrowths with related framework types^17,18^, and other types of defects like Si or Al framework vacancies and associated silanol nests^19^, and pore blockages by extra-framework matter^20,21^.

These microstructural characteristics are the *output* of a batch crystallization process, whose *inputs* include the chemical composition of the mixture, the chemicals and sequence of steps used to prepare this mixture, the temperature and time of crystallization, and the extent of mixing during crystallization (e.g., static or rotating autoclaves)^21,22^. Additional variations further expand the range of synthesis *inputs* that can affect the crystallization *output*. For example, mid-synthesis changes in composition and temperature during crystallization can have a significant effect on crystal size and framework type^11,13^. Crystallization mixtures used for zeolite synthesis contain species varying from small ions to colloidal particles and gels, the interconversions and interactions of which cannot be predicted quantitatively^23^. Therefore, the ability to determine the effect of crystallization *inputs* on the microstructural outcome (*output*) is very limited, and microstructural optimization requires a large number of experiments exploring all possible *input* combinations^24,25^. Here, it is demonstrated that Machine Learning algorithms can be used to quantitatively capture the effect of crystallization *inputs* on key microstructural characteristics (*outputs*) of faujasite, which is widely used as a catalyst in fluid catalytic cracking and as an adsorbent for oxygen/nitrogen separation^21,26,27^. Comprehensive combinations of crystal morphologies, composition and phase purity are reported, and improved catalytic properties are demonstrated.

## Results

**Fig. 1:**
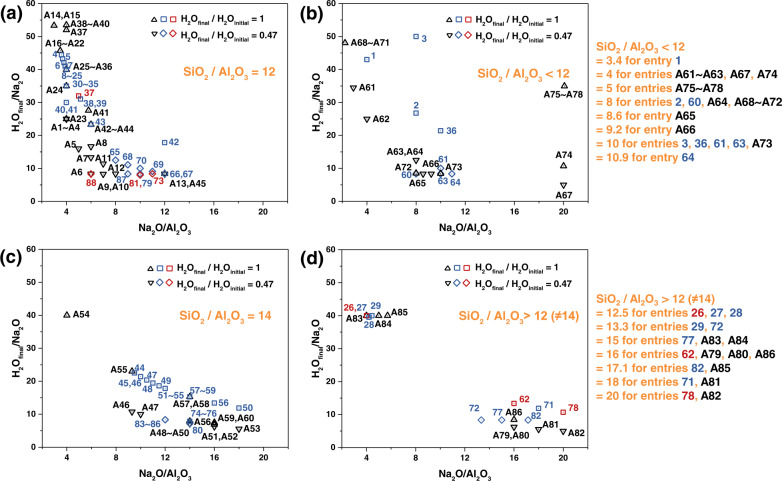
Explored region in composition space aiming at pure FAU and FAU/EMT zeolite synthesis. x- and y- axes are Na_2_O/Al_2_O_3_ (input), H_2_O_final_/Na_2_O (input), respectively. The 174 (88 + 86) synthesis experiments can be divided into three groups based on the SiO_2_/Al_2_O_3_ ratio of the synthesis mixture: (**a**) SiO_2_/Al_2_O_3_ = 12, (**b**) SiO_2_/Al_2_O_3_ < 12, (**c**) SiO_2_/Al_2_O_3_ = 14, and (**d**) SiO_2_/Al_2_O_3_ > 12 (other than 14). Square (□) and diamond (◇) points (in blue and red color, see below for explanation of distinction based on color) represent pure faujasite zeolites (FAU and FAU/EMT) without (H_2_O_final_/H_2_O_initial_ = 1) and with (H_2_O_final_/H_2_O_initial_ = 0.47) the freeze drying step, respectively. Black up (△) and down (▽) triangle points represent tried recipes leading to other zeolites (GIS, SOD, etc.) or amorphous phases. Entry number is listed in the Figs., and entry details are provided in the Supporting Information (Tables S1 and S2). Entries A1-A86 (Supplementary Table 2) contain other zeolites (GIS, SOD, etc.) or amorphous phases instead of pure faujasite; entries 1-88 (Supplementary Table 1) are pure faujasite zeolites (FAU or FAU/EMT) used for ML algorithm training (represented in blue) and testing (represented in red). Details of entry points are provided in Supplementary Fig. 1.

The focus is on the synthesis of the zeolite faujasite, and we aim to prepare faujasite crystals with a combination of characteristics (outputs): Si/Al ratio, crystal size, particle size, FAU/EMT ratio, microporosity. Figure 1 summarizes experiments performed initially to outline the region in composition space (details are provided in Supplementary Fig. 1), which results in pure faujasite (i.e., FAU, EMT or FAU/EMT intergrowths). The initial selection of the synthesis region in Fig. 1 is based on our prior works^13,28^ and prior work by Rimer et al. ^29^, which empirically explored and broadened the boundaries of faujasite synthesis conditions. Within this region, we performed 174 synthesis experiments. From these, 86 experiments (indicated by A1-A86 in Fig. 1, Supplementary Fig. 1, and Supplementary Table 2) did not produce pure FAU or FAU/EMT, and these entries are excluded from further analysis. The remaining 88 experiments (indicated by 1-88 in Fig. 1, Supplementary Fig. 1, and Supplementary Table 1) were used for training (81 entries) and testing (7 entries) of the ML algorithm (the latter suggests 4 more entries as prediction points), except when analyzing crystal size, where we have excluded crystal sizes larger than 60 nm and used 46 experiments (42 entries for training, and 4 entries for testing).

Our synthesis involves 5 parameters representing the crystallization mixture composition (x, y, z, m, n) x SiO_2_: y Al_2_O_3_: z Na_2_O: m H_2_O_initial_ (n H_2_O_final_). The initial and final water contents indicate the water present during the aging and crystallization steps, respectively. In some synthesis experiments they are the same, i.e., there is no adjustment of the water content, while in others the water content is reduced by freeze drying to set the ratio of H_2_O_final_/ H_2_O_initial_ equal to ca. 0.47. In all experiments, we use Al_2_O_3_ content as a basis by setting it equal to 1. We have then four independent parameters to describe the composition of our mixtures, i.e., the relative ratios of Na_2_O/Al_2_O_3_, SiO_2_/Al_2_O_3_, H_2_O_final_/Na_2_O, and H_2_O_final_/H_2_O_initial_. Figure 1 shows all experiments in plots of H_2_O_final_/Na_2_O versus Na_2_O/Al_2_O_3_ for different SiO_2_/Al_2_O_3_ ratios (SiO_2_/Al_2_O_3_ = 12, <12, 14, and >12 (other than 14) in Fig. 1(a–d), respectively, and details are provided in Supplementary Fig. 1), and reflects the two H_2_O_final_/H_2_O_initial_ levels of 1 and 0.47. In addition to the four independent parameters needed to describe the composition of the mixture, we have five other synthesis parameters: the source of silica, the source of alumina, the type of oven used (rotation vs. static autoclave, or oil bath), and the crystallization time and temperature (for all experiments aging was performed at 25 °C for 24 h with stirring).

In total, we have 9 independent parameters (inputs) that describe the synthesis (processing) conditions. For the 88 experiments that gave FAU or FAU/EMT with no other phases, we determined 5 microstructural characteristics (structure): the Si/Al ratio by ICP, the particle size by TEM and/or SEM images, the crystal size from XRD peak broadening (in our analysis, we only considered crystal sizes smaller than 60 nm), the degree of intergrowth represented as FAU/(FAU + EMT) (determined by analysis of XRD data), and the Ar adsorption at *p*/*p*_0_ = 0.01 as an indication of the microporosity. These five quantities/microstructural characteristics represent the outputs of the crystallization process; we have also considered as a separate, sixth output, the ratio of particle over crystal size (for crystal sizes smaller than 60 nm), as a measure of the level of aggregation. The characterization results for the 88 experiments are presented in Section S2 (Tables S3–S25 and Supplementary Figs. S2–S24).

In many branches of materials science, both experimental and computational (including metal additive manufacturing^30^, polymer science^31^, and drug design^32^ and delivery^33^), there have been extensive studies of structure-property relations with the help of ML. An important ingredient of these is the knowledge—whether from first principles, experience, or intuition—of the appropriate *structural features* that correlate the properties of interest. ML holds the promise of turning such correlations from an informed art to a reliable, data-driven, computer assisted process; learning such correlations can then lead to the educated design and optimization of developed materials^33–35^. The processing/fabrication of materials with desired structure is an equally (if not more challenging) problem; data science and ML have the potential to be transformative in deriving processing-structure relations, leading to breakthroughs in ultimately establishing the ideal “processing-structure-property” pathway to materials design^30–34,36–39^.

ML algorithms have proven useful for predicting both quantitative and discrete characteristics of various zeolitic materials. Carr et al.^40^ constructed a classifier based on the topology of zeolites into different mineral types and framework types^41,42^. Coudert et al.^43,44^ used ML algorithms on data from DFT computations to construct a link between structural properties and mechanical properties. Moliner et al.^45^ discussed the potential of ML in zeolites synthesis (a) in the construction of high throughput platforms, (b) in the prediction of stable structures for zeolites and guidance of the zeolite synthesis involved with different structures, and (c) in automated data extraction. Gurney et al.^46^ presented different ML tools that can be key elements for an ML-based design and discovery of zeolites and other crystalline materials. Ducamp et al.^38^ used DFT data to construct a structure-property relation between features of the geometry, topology, and porosity of the zeolitic materials, and their thermal properties. Jensen et al.^47^ built a text mining pipeline for extracting zeolite synthesis data from a database of ~70,000 relevant journal articles. They further constructed, through ML, an input-output relationship between synthesis conditions and framework density of zeolitic materials.

Here, we study the synthesis (ingredients, composition, processing conditions, operating protocols) leading to the fabrication of faujasite zeolite; and explore the capabilities and avenues that ML opens toward the optimization of desired microstructural characteristics like the framework Si/Al ratio. Selecting appropriate synthesis conditions leading to a particular set of microstructural characteristics is challenging, since the known crystallization mechanism is not adequate to derive predictive models^45^. ML algorithms can be used to construct experimentally-informed candidate input-output (processing/structure) relationships from data in the absence of closed-form (physics-driven) expressions. In our case, given processing/structure information for zeolite fabrication, we aim to construct a function that maps synthesis conditions to final structure. Positing such a model allows us to estimate, predict, and even optimize structure of a zeolite material given unexplored synthesis conditions, thus guiding further experimentation.

Learning a candidate mapping between inputs and outputs can be attempted through several, in principle comparable, ML approaches, including Neural Networks (NN), Gaussian Process Regression (GPR), and Geometric Harmonics (GH). This paper focuses primarily on predicting via GH, but we also provide comparisons with the other two methods in order to illustrate the qualitative similarity of corresponding results. All methods use input and corresponding output data from a (posited) function of interest to construct a surrogate model (an approximation) of the true function. To the best of our knowledge, Diffusion Maps/Geometric Harmonics have not been previously used in this context. We provide a brief description of each method below and additional details in the SI (Section S2, Supplementary Figs. S25–S27).

Geometric Harmonics (GH) uses the input-output data to numerically construct a hierarchical set of data-driven basis functions (to be exact, basis vectors, that constitute discretized versions of basis functions) in the space of inputs. Any function of the inputs (e.g., a structural characteristic of the resulting material) can be approximated as a linear combination of the leading (data-driven) basis functions, in the same spirit as a function of space can be approximated by a truncated sum of its Fourier components^48,49^.

Similarly, a Neural Network (NN) can construct a surrogate function $f$ between inputs (synthesis conditions), ${{{{{\boldsymbol{x}}}}}}$, and outputs (structural characteristics), $y=f\left({{{{{\boldsymbol{x}}}}}};{{{{{\boldsymbol{\theta }}}}}}\right)$, by adapting the values of the parameters ${{{{{\boldsymbol{\theta }}}}}}$ to achieve the best function approximation^50^. The selection of the parameters is achieved via an optimization stage that is called *training*. During training, the network computes derivatives with respect to its parameters ${{{{{\boldsymbol{\theta }}}}}}$ in order to minimize a loss function across the training data^46,50,51^.

Gaussian Process Regression (GPR) models an output function $f$ of the inputs as a collection of jointly normal random variables that describe one’s knowledge about $f({{{{{\boldsymbol{x}}}}}})$ at each point ${{{{{\boldsymbol{x}}}}}}$ in the function’s domain^52^. After the user specifies (via a kernel function) how these random variables are correlated with each other, conditional probability allows one to predict the function value ${y}^{{\prime} }=f\left({{{{{{\boldsymbol{x}}}}}}}^{{\prime} }\right)$ at another input point ${{{{{{\boldsymbol{x}}}}}}}^{{\prime} }$. Such a result is expressed as a Gaussian distribution, from which the mean may serve as the prediction and the variance as a measure of uncertainty in the estimate^53^.

**Fig. 2:**
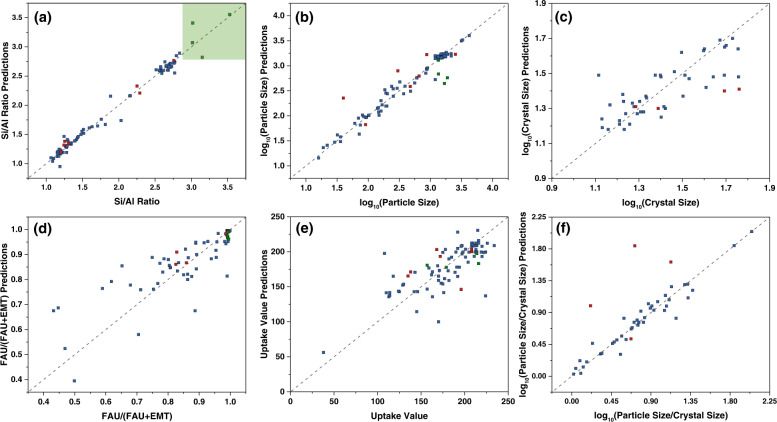
Comparison between experimental values and predicted values via the Machine Learning algorithm we label “rescaled 10-CV” (a Geometric Harmonics algorithm). **a** Si/Al ratio via ICP analysis; the green square represents outcome from an input region we selected to explore guided by our surrogate model to enhance the Si/Al ratio to values higher than 3, (**b**) log_10_(Particle size) measured from SEM/TEM images, (**c**) log_10_(Crystal size) via XRD patterns peak-broadening analysis in accordance with the Scherrer equation using FAU(311) and FAU(331) reflections (only crystal sizes smaller than 60 nm are included), (**d**) FAU fraction via deconvolution of the first main diffraction peak in XRD patterns, namely the peak area ratio of FAU(111)/(FAU(111) + EMT(100)), (**e**) Uptake values at *P*/*P*_0_ = 0.01 via Ar-adsorption isotherms, (**f**) log_10_(particle size to crystal size ratio), lower value means lower aggregation of FAU particles. Blue dots represent training points for model identification, red dots represent testing points for the identified model, and green dots represent prediction points toward obtaining high-silica FAU zeolites. Entry numbers for dots in (**a**–**f**) are labelled in Supplementary Fig. 28. Fewer points were involved in (**c**) and (**f**), since we only consider entries with crystal sizes smaller than 60 nm for the crystal size analysis.

We characterize the five outputs of each experiment as functions of nine input quantities. Because six of the inputs are numerical and three of the inputs (oven type, silica source, and alumina source) are categorical, the input for each experiment is represented as a vector in 12-dimensional space (see Section S4 for explanation of the number of dimensions). We developed forty-four GH (nine for Si/Al ratio, etc., see Section S4.2), two NN, and five GPR models, which are described in detail in Section S4 of the SI. Comparisons of experimental and predicted microstructural characteristics (outputs) from one of these models (i.e., GH with rescaled inputs and 10-fold cross validation: “rescaled 10-CV”) are shown in Fig. 2 (Details of entry points are provided in Supplementary Fig. 28). For crystal size, we only include experiments and predictions for sizes smaller than 60 nm to ensure that instrumental broadening does not affect the experimental measurements. In addition to the five outputs discussed earlier, we also consider the particle to crystal size ratio as a measure for differentiating single from aggregated/intergrown crystals.

Figure 2 shows that ML can approximate output functions of interest (entry numbers for points in Fig. 2(a–f) are labelled in Supplementary Fig. 28). We use a set of 81 experiments as training points (represented as blue points in Fig. 2(a–f)) and 7 experiments as testing points (represented as red points in Fig. 2(a–f)). In addition, the green points are for unseen experiments, which will be discussed later. The blue, red, green color scheme, indicating training, testing, and prediction, respectively, is used in Figs. 1 and 2, Supplementary Table 1, Supplementary Tables 3–25, Supplementary Figs. 28–37.

All our GH, GPR, and NN models were able to learn a surrogate model from the training data, choosing hyperparameter values based on cross-validation, log-likelihood maximization, and ADAM minimization of training mean squared error, respectively. These models also performed well on the test set, which consisted of experiments that had already been performed but were set aside for this purpose. Predictions from the different algorithms are provided in the Supporting Information (Supplementary Figs. 28–37). In order to compare the reliability of these models, we applied error analysis and calculated the *R*^2^ and MSE values for each model and each output from the training (blue entries) and test (red entries) sets as listed in Supplementary Table 26. Error analysis results (Supplementary Table 26) show that if we select MSE training as a performance metric “rescaled 10-CV” is best for Si/Al (second smallest value for MSE training and smallest value for MSE testing) followed by “rescaled LOOCV (w/o categorical)” (ranking third for MSE training and second for MSE test). By comparison, “rescaled 10-CV (w/o categorical)” has the smallest value for MSE training but ranks fourth for MSE test. Therefore, the algorithm “rescaled 10-CV” is the most reliable one among different CV schemes, and correlations developed by this algorithm are provided in Fig. 2. Correlations for the algorithm “rescaled LOOCV (w/o categorical)” are provided in Supplementary Fig. 36.

An example of training synthesis is Entry 3 with a molar composition of 10 SiO_2_: 1 Al_2_O_3_: 8 Na_2_O: 400 H_2_O, which is heated at 100 °C for 18 h in a static autoclave. It leads to the synthesis of large pure FAU crystals with Si/Al ratio of 1.6. In our prior work, we used this FAU material to investigate the accessibility and reactivity of protons located within sodalite cages of the FAU framework that become accessible during ion exchange^54^.

An example of a test synthesis is Entry 81, with an initial molar composition of 12 SiO_2_: 1 Al_2_O_3_: 10 Na_2_O: 180 H_2_O, which after aging at 25 °C for 24 h was subjected to water reduction by freeze drying to 80 H_2_O and was then heated at 50 °C for 4 days in a rotating autoclave (6 rpm). The trained model predicts the output of this test synthesis that leads to non-aggregated high FAU content nanocrystals with Si/Al ratio of 1.3. Such low Si/Al ratio nanocrystals could be useful for the fabrication of FAU membranes.

**Fig. 3:**
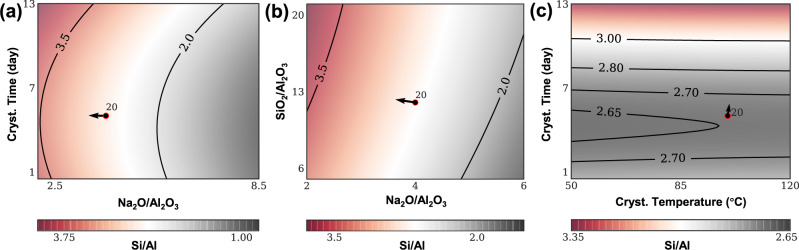
Predictions for unseen experiments to exceed Si/Al = 3. Predictions for Si/Al ratio in different regions of input space. Each subplot varies two quantities at a time. **a** GH prediction (“rescaled 10-CV”) as crystallization time and Na_2_O change. **b** GH prediction (“rescaled 10-CV”) as SiO_2_ and Na_2_O change. **c** GH prediction (“rescaled 10-CV”) as crystallization time and crystallization temperature change. These three figures illustrate neighborhoods of entry 20, which was made using molar composition 12 SiO_2_: 1 Al_2_O_3_: 4 Na_2_O: 160 H_2_O, 100 °C for 5 days under static conditions; the arrow indicates (the projection of) the Si/Al gradient’s direction.

ML was then used to suggest inputs aiming at achieving the desirable output of FAU with Si/Al ratio higher than 3. Exceeding a Si/Al of 3, by direct synthesis (i.e., without dealumination treatments)^21^ in the absence of organic-structure-directing agents (OSDA) in sodium aluminosilicate sols/gels remains elusive despite many decades of effort in developing FAU synthesis methods, e.g., OSDA structure design (bottom-up)^22,26^, post-synthesis dealumination (top-down)^21,26^, etc. It is a highly desirable outcome, as it may lead to robust catalytic properties^29^, improved stability^26^, and lower manufacturing cost^11^. We test the ability of the best algorithm to predict properties for unseen synthesis conditions by using it as a surrogate model for optimization. Since our input space is 12-dimensional, we use plots that vary two quantities at a time while keeping others fixed, which produces hyperplane “slices” of the complete model in the synthesis conditions space as seen in Fig. 3, which provides projective views of the GH predictions for Si/Al ratio, as a function of different pairs of process parameters (input variables). Entry 20 was reported from the prior work by Rimer et al.^29^, and this entry holds the reported hitherto highest Si/Al ratio to prepare high-silica faujasite zeolites via template-free routes. The gradients were computed at entry 20 in our training set (Supplementary Table 1) as the base point. Supplementary Fig. 39 includes two similar plots of predictions for a model (Supplementary Fig. 39(a)(b)) that instead uses a Matérn(0.5) kernel (see method description in Section S4) and counterparts developed by the algorithm LOOCV (Supplementary Fig. 39(c)(d)). Therefore, the predicted contours and gradients developed from multiple ML models suggest decreasing Na_2_O (or reducing pH) and increasing the crystallization time to achieve Si/Al > 3.

**Fig. 4:**
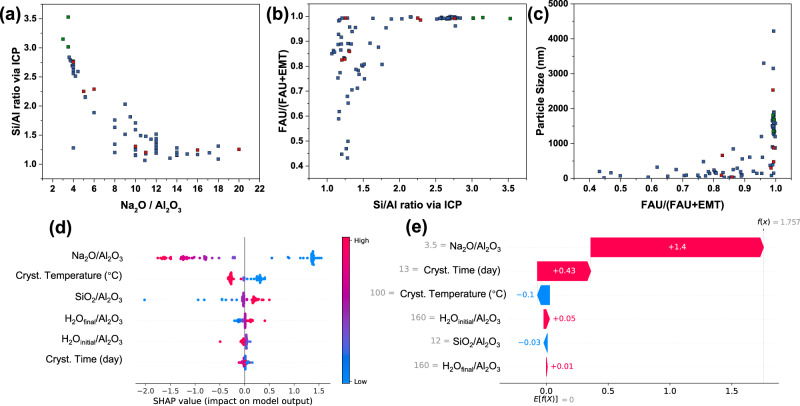
Input/output correlations from raw data and SHAP (Shapley value based) analyses. **a** Correlation between Na_2_O/Al_2_O_3_ in synthesis mixture (input) and Si/Al ratio of product via ICP (output). **b** Correlation between Si/Al ratio via ICP (output) and FAU fraction (output), the latter refers to FAU/(FAU + EMT). **c** Correlation between FAU fraction (output) and particle size (output). In (**a**–**c**), blue/red/green dots represent training/testing/prediction points, respectively. **d** The constructed SHAP summary plot for the trained Si/Al model “rescaled LOOCV (w/o categorical)”. **e** The waterfall plot on the model “rescaled LOOCV (w/o categorical)” for entry 91 indicating the contribution of each variable (synthesis condition) for the predicted normalized output of the model $f(x)$. To the estimated value of the output $f(x)$ the denormalization ${f}_{{True}}\left(x\right)=f\left(x\right){\sigma }_{{Si}/{Al}}+{\mu }_{{Si}/{Al}}$ is needed to recover the true values, where ${\sigma }_{{Si}/{Al}}$ corresponds to the standard deviation and ${\mu }_{{Si}/{Al}}$ corresponds to the mean of the training set.

An inspection of input/output correlations from just plotting the raw data (Supplementary Figs. 40–43), which demonstrate the dependences among these variables and the complexity of the zeolite synthesis itself, also indicates that low Na_2_O increases the Si/Al ratio (as shown in entries of Supplementary Table 1, Fig. 4(a), and Supplementary Fig. 41). Indeed entries 4-8 of Supplementary Table 1 show the progressive increase of Si/Al from 2.7 to 2.8 as the Na_2_O/Al_2_O_3_ ratio decreases from 4 to 3.6. Lower Na_2_O/Al_2_O_3_ entries, e.g., A16 to A22 of Supplementary Table 2 with Na_2_O/Al_2_O_3_ ratio of 3.5, have been, however, excluded from the training set because they yield amorphous or impure (e.g., mixtures of FAU + LTA) products. In particular, entries A22 and A20, which were performed at 100 °C (i.e., same temperature as entries 4–8 discussed above) for 3 and 7 days yield products that are either amorphous or amorphous mixed with some FAU, respectively. A potential explanation for this observation is that as Na_2_O is being reduced, the associated pH reduction slows down the crystallization kinetics. Therefore, a possible path to FAU with Si/Al > 3 would be to increase the time and/or temperature of crystallization. Since increasing the temperature to 120 °C (entries A16-A19 and A21) also yields FAU with amorphous or LTA impurities, we decided to explore longer crystallization times at 100 °C. Entries 89–92, with crystallization times 9–13 days, yield pure FAU with Si/Al larger than 3.

The best performing algorithm (based on MSE training in Supplementary Table 26) “rescaled 10-CV” predicts this outcome (see Fig. 2(a) and Supplementary Fig. 28(a)). From the remaining models, the second best performer “rescaled LOOCV (w/o categorical)” also makes good predictions. The rest, except for two models (normalized 10-CV and rescaled 5-CV), fail to predict Si/Al > 3. The “rescaled 10-CV” model successfully predicts additional outcomes of the synthesis for entries 89–92. Particle Size, FAU/(FAU + EMT) ratio, and Uptake Values are shown in Fig. 2 and Supplementary Fig. 28. We note that since the crystal sizes for entries 89–92, as determined from XRD peak broadening, are larger than 60 nm, they are not included in the plots of Supplementary Fig. 28(c) and 28(f). Once Si/Al ratio exceeds 2, FAU fraction increases to near unity, and the high Si/Al materials are pure FAU products (Fig. 4(b)). Similarly, particle size consistently increases with increasing Si/Al (Fig. 4(c)).

Despite the small number of training and testing data, it can be concluded that the selected best performing Geometric Harmonics algorithm (“rescaled 10-CV”) is successful in predicting the outcome of unseen experiments with different combinations of properties (outputs). These predictions can also steer experimental conditions (inputs) to achieve desirable outcomes. On the contrary, Neural Networks and Gaussian Process Regression were not successful in providing good predictions.

The dominant role of Na_2_O is evident by the input/output correlation of Fig. 4(a). It becomes also evident in SHAP (Shapley value based) analyses^55–57^ (Fig. 4(d) and (e), Supplementary Fig. 44). The Shapely values measure the average contribution of each feature’s (variable’s) value to the prediction and thus provide a sense of how the change of a variable might affect the output^56,57^. We applied the model-agnostic exact explainer algorithm^57^ on the model Si/Al trained with LOOCV, rescaling as preprocessing and without categorical inputs (namely the “rescaled LOOCV (w/o) categorical” model). The selection of this model was made based on its performance (second best based on its MSE metrics) and on the fact that, by excluding the categorical inputs, all of its inputs are continuous. The SHAP analysis on the Si/Al model trained with “rescaled 10-CV” is provided in SI (Supplementary Fig. 45). We generate the summary plot (Fig. 4(d)) for all the training points to get a sense of the importance of contribution of each variable (synthesis condition). In the Fig. 4(d), the x-axis is the Shapely value that indicates the contribution of a particular feature to the output. The y-axis reports the variables (synthesis conditions), and the color corresponds to the magnitude of the value for each variable if it is large or small. The variables are sorted in descending order based on their contribution. SHAP suggests that Na_2_O contributes the most to the output (Si/Al) and that deceasing relative Na_2_O amount (Na_2_O/Al_2_O_3_) will contribute positively to the output (increase the Si/Al). The SHAP analysis for the model “rescaled 10-CV” provides a similar conclusion regarding the role of Na_2_O/Al_2_O_3_, being most prominent and contributing positively to the output when decreasing (Supplementary Fig. 45).

For the four prediction points after optimization (89, 90, 91, and 92) the Shapely values predicted separately. The waterfall plot for the entry 91 is shown in Fig. 4(e) and for the remaining prediction points we included their waterfall plots in Supplementary Fig. 44. In the waterfall plot, the x-axis corresponds to the normalized values of the output variable (Si/Al ratio) $f(x)$. To obtain the true ${f}_{{True}}\left(x\right)$ values for the output, denormalization is needed: ${f}_{{True}}\left(x\right)=f\left(x\right){\sigma }_{{Si}/{Al}}+{\mu }_{{Si}/{Al}},$ where ${\sigma }_{{Si}/{Al}}$ corresponds to the standard deviation of the training set, ${\sigma }_{{Si}/{Al}}=0.67$, and ${\mu }_{{Si}/{Al}}$ corresponds to the mean of the training set, ${\mu }_{{Si}/{Al}}=1.91$. The Shapely value of each feature is given by the length of the bar. If the contribution is positive is colored red and if is negative is colored blue. The (absolute) Shapely values shows how much a single variable affects the prediction. In Fig. 4(e) it appears that the change in Na_2_O and Crystallization Temperature positively affects (increases) the output prediction of the model.

**Fig. 5:**
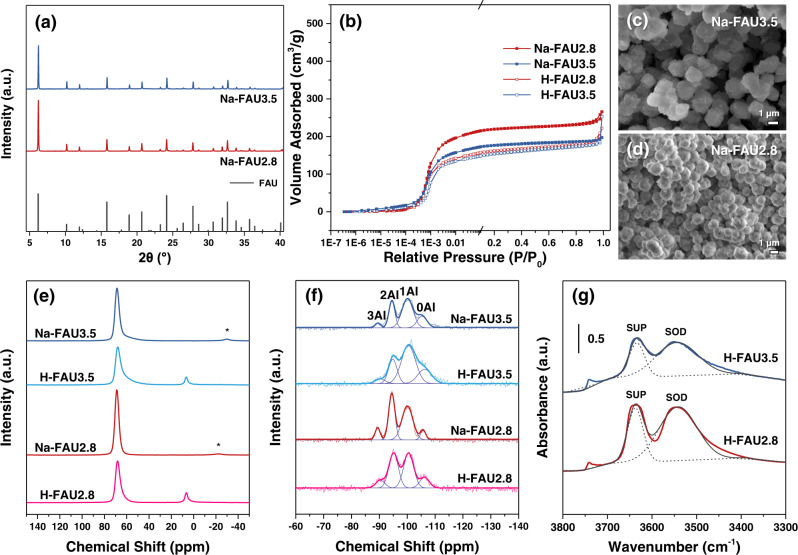
Characterization results of FAU3.5 and FAU2.8 materials. **a** XRD patterns (plotted for the CuKα wavelength of 1.54059 Å) for Na-FAU3.5 and Na-FAU2.8 zeolites converted from synchrotron XRD patterns (obtained using 0.45228 Å), and the standard pattern of FAU zeolite (PDF#38-0240) is provided on the bottom. **b** Ar-adsorption isotherms for different FAU zeolites at 87 K, in which *P*/*P*_0_ from 10^−6^ to 0.1 is plotted logarithmically and *P*/*P*_0_ from 0.1 to 1.0 is plotted linearly. SEM images for (**c**) Na-FAU3.5 and (**d**) Na-FAU2.8, the scale bar = 1 μm for (**c**) and (**d**). **e** Solid-state ^27^Al-NMR spectra for different FAU zeolites. Asterisks denote spinning side bands^29^. **f** Solid-state ^29^Si-NMR spectra for different FAU zeolites reflecting Q^4^ silicon species coordinated with *n* OAl bonds (where *n* = 0, 1, 2, and 3). **g** Infrared spectra after dehydration at 603 K over H-FAU3.5 and H-FAU2.8 zeolites. Black dash lines centered at ~3640 and ~3550 cm^−1^ are ascribed to protons located within supercages (SUP) and sodalite cages (SOD), respectively. Black solid lines refer to cumulative lines from deconvolution.

Next, we compared this as-synthesized faujasite (Na-FAU3.5) with the previously reported highest Si/Al-ratio faujasite made by direct synthesis (Na-FAU2.8 with a Si/Al ratio of 2.8 prepared from the composition of 12 SiO_2_: 1 Al_2_O_3_: 4 Na_2_O: 160 H_2_O)^29^. XRD patterns (Fig. 5(a)) show that these two faujasite materials are pure FAU without EMT intergrowths, or other impurities. According to Ar-adsorption isotherms (Fig. 5(b)), although Na-FAU3.5 has a lower uptake value at *P*/*P*_0_ = 0.01 than Na-FAU2.8, the corresponding ion exchanged forms, H-FAU3.5 and H-FAU2.8 exhibit similar isotherms (ion exchange for both was performed using 1 M of NH_4_NO_3_ solution for 1 h, and 0.25 g zeolite powder per 40 cm^3^ of ammonium solution). As shown in SEM images (Fig. 5(c)(d)), Na-FAU3.5 exhibited a larger particle size than Na-FAU2.8. Solid-state ^27^Al-NMR (Fig. 5(e)) proved that both Na-FAU materials did not contain octahedral Al species (typically observed at a chemical shift δ of 0 ppm)^29^ prior to ion exchange, reflecting integrity of the FAU framework and the absence of extra-framework Al. Extra-framework Al species were formed only after ion exchange^16,54^. The framework Si/Al ratios (Table 1, columns 4&5, Supplementary Table 27) could be estimated from the ^29^Si-NMR spectra (Fig. 5(f)) based on “Loewenstein’s rule” (Eq. 1)^58^, which stipulates the absence of Al-O-Al linkages in the zeolite framework.

1 $$\frac{{{{{{\rm{Si}}}}}}}{{{{{{\rm{Al}}}}}}}=\mathop{\sum }\limits_{x=0}^{4}{I}_{{{{{{\rm{Si}}}}}}{({{{{{\rm{OAl}}}}}})}_{x}}/0.25\mathop{\sum }\limits_{x=0}^{4}x{I}_{{{{{{\rm{Si}}}}}}{({{{{{\rm{OAl}}}}}})}_{x}}$$

**Table 1:** Physical properties of H-FAU3.5 and H-FAU2.8 zeolites

Zeolite^a^	Si/Al ratio^b^	Na/Al ratio^b^	(Si/Al)_F, Na-FAU_ ratio^c^	(Si/Al)_F, H-FAU_ ratio^c^	Chemical formula per zeolite unit cell^d^	Proton density (μmol/g)^e^	H_SOD_/H_SUP_ (infrared)^f^	H_SOD_ (μmol/g)^g^	H_SUP_ (μmol/g)^g^
H-FAU2.8	2.8	0.32	2.73	2.79	(Al_2_O_3_)_0.6_(Na_16_·65H_2_O)H_34.4_Al_51_Si_141_O_384_	2630	3.03	1977	653
H-FAU3.5	3.5	0.50	3.48	3.63	(Al_2_O_3_)_0.9_(Na_21_·83H_2_O)H_20.7_Al_41_Si_151_O_384_	1529	3.24	1168	361

^a^2.8 and 3.5 were measure from ICP analysis for corresponding Na-FAU zeolites. Na-FAU3.5 was prepared from entry 91 of Supplementary Table 1, and Na-FAU2.8 was prepared from entry 24 of Supplementary Table 1.

^b^From ICP analysis for H-FAU zeolites.

^c^Estimated from ^29^Si-NMR spectra for Na-FAU and H-FAU zeolites respectively in accordance with “Loewenstein’s rule” (see Section S5 in SI, Supplementary Table 27)^58^.

^d^Calculated via a combination of framework Si/Al ratio for Na-FAU (column 4), Na/Al ratio for H-FAU (column 3), and framework Si/Al ratio for H-FAU (column 5), considering that the total number of T atoms per unit cell is 192 and each Na^+^ cation is coordinated with four water molecules^65,66^. Extra-framework aluminum content is approximated as Al_2_O_3_ and is provided along with the zeolite unit cell formula. All numbers that appear in chemical formulae are rounded to the nearest integer, except H and Al_2_O_3_ components.

^e^Calculated by dividing the specific H^+^ number per unit cell by the molecular weight using chemical formulae (column 6).

^f^Calculated from the equation of H_SOD_/H_SUP_ = (OH_3550_/ε_3550_)/(OH_3640_/ε_3640_) based on the Lambert-Beer law, and the infrared spectra of dehydrated H-FAU zeolites are shown in Fig. 5(g). Here, extinction coefficients *ε*(OH)_3640_ = 6.76 cm/μmol, and *ε*(OH)_3550_ = 5.39 cm/μmol^59,67^.

^g^Calculated from the equations of H_SOD_ = H_SOD_/(H_SOD_ + H_SUP_) × proton density and H_SUP_ = H_SUP_/(H_SOD_ + H_SUP_) × proton density, where proton densities and H_SOD_/H_SUP_ values are provided in columns 7 and 8, respectively.

These framework Si/Al ratios (Table 1, columns 4&5) over H-FAU materials can be combined with Na/Al ratios via ICP analysis (Table 1, column 3) to calculate chemical formulae (Table 1, column 6), and determine H^+^ site densities over these two H-FAU materials (Table 1, column 7). Infrared spectra recorded upon adsorption of pyridine (Supplementary Fig. 46) show that pyridine molecules only titrate protons (at ~3640 cm^−1^)^54,59^ located within supercages over these two H-FAU materials. Protons with sodalite cages are able to be fully titrated only when the framework collapses partially (e.g., steam treatment of FAU materials to prepare ultra-stable Y)^60^. Both H-FAU3.5 and H-FAU2.8 zeolites still sustain bulk framework stability as evidenced by Infrared spectra recorded upon adsorption of pyridine (Supplementary Fig. 46) and Ar-adsorption isotherms (Fig. 5(b)) after ion exchange with 1 M of NH_4_NO_3_ solution. Our prior work compared the reactivities and selectivities for protolytic reactions of propane between protons within opened sodalite cages and protons within supercages over high-silica faujasite zeolites^54^. We reported in this prior work that sodalite cages could be fully opened when 0.6 M of NH_4_NO_3_ solution was used to perform ion exchange by virtue of infrared spectra of H-D exchange with deuterated propane^54^. Thus, upon ion exchange using 1 M of NH_4_NO_3_ solution, protons within both sodalite cages and supercages could be titrated by propane. Infrared spectra after dehydration at 603 K (Fig. 5(g)) reflect that H-FAU3.5 and H-FAU2.8 zeolites exhibit a similar proton density ratio of H_SOD_/H_SUP_ (Table 1, column 8). We also observed in our prior work that unlike low-silica FAU zeolites, which contain protons on site II and site III within supercages, high-silica FAU zeolites only contain protons on site II within supercages^61^. Thus, H-FAU3.5 and H-FAU2.8 zeolites possess similar H_SOD_/H_SUP_ ratios and the same atomic configurations (i.e., protons on site II within supercages, and protons on site I′ within sodalite cages).

Having now established the similarities of the two materials in terms of phase purity, porosity, particle size, extra-framework Al, acid site density and location, we proceed to compare their catalytic performance. We compared reactivities of protons on H-FAU3.5 and H-FAU2.8 zeolites by using molecular dehydrogenation and cracking of propane (Eqs. 2 and 3) as probe reactions.

2 $${{{{{{\rm{C}}}}}}}_{3}{{{{{{\rm{H}}}}}}}_{8}\mathop{\longrightarrow }\limits^{{k}_{{{{{{\rm{D}}}}}}}}{{{{{{\rm{C}}}}}}}_{3}{{{{{{\rm{H}}}}}}}_{6}+{{{{{{\rm{H}}}}}}}_{2}$$

3 $${{{{{{\rm{C}}}}}}}_{3}{{{{{{\rm{H}}}}}}}_{8}\mathop{\longrightarrow }\limits^{{k}_{{{{{{\rm{C}}}}}}}}{{{{{{\rm{C}}}}}}}_{2}{{{{{{\rm{H}}}}}}}_{4}+{{{{{{\rm{CH}}}}}}}_{4}$$

**Fig. 6:**
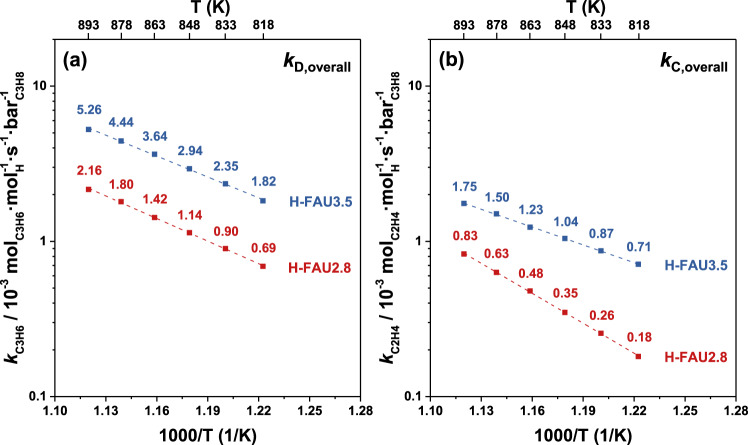
Analysis of rate constants for molecular cracking and dehydrogenation of propane over H-FAU3.5 and H-FAU2.8 zeolites. Temperature dependence of rate constants on a per overall H^+^ site basis for (**a**) dehydrogenation and (**b**) cracking over H-FAU3.5 and H-FAU2.8 zeolites. Reaction conditions: H_2_/C_3_H_8_/Ar/He = 3/3/1.5/60, with a total pressure of 120 kPa and a total flow rate of 67.5 sccm, and space velocity = 3600 cm^3^_C3H8_·g_cat_^−1^ h^−1^, time-on-stream = 60 s. Propane conversions are <1%.

Gounder et al.^62^ reported that alkane dehydrogenation can be promoted over extrinsic active sites of carbonaceous deposits formed during reaction, and the removal of remnant reactive carbon species should be taken into consideration to precisely assess intrinsic H^+^- catalyzed propylene formation rates. Sample pretreatment in H_2_, and H_2_ co-feed in the inlet stream were thus incorporated in the experimental protocol to mitigate on-stream deposition of reactive carbon species. Two H-FAU samples were pretreated using H_2_/He mixtures (*p*_H2_ = 35 kPa, and H_2_/He = 1:2) and co-fed H_2_ (H_2_/C_3_H_8_/Ar/He = 3/3/1.5/60, *p*_H2_ = 5.3 kPa,) in the inlet stream at different temperatures (818, 833, 848, 863, 878, and 893 K). Once protons within sodalite cages are rendered accessible by partial framework collapse upon ion exchange at NH_4_NO_3_ concentrations exceeding 0.6 M, then propane dehydrogenation and cracking occurs both over H^+^ sites in the sodalite cage and in the supercage as we reported previously^54^. Since these two H-FAU samples share similar proton density ratios (H_SOD_/H_SUP_ in Table 1, column 8), we directly normalized rates per overall H^+^ site. By comparison, H-FAU3.5 exhibits higher propane dehydrogenation and cracking rate constants per overall H^+^ site than H-FAU2.8 (Fig. 6). Despite the lower proton density (Table 1, column 7), H-FAU3.5 also exhibits higher rate constants per gram (Supplementary Fig. 47). We surmise that these two samples were partially dealuminated at the harsh ion exchange conditions (1 M NH_4_NO_3_) used, which could be proved by ^27^Al-NMR spectra (Fig. 5(e)). Lercher et al.^63^ have examined solid state reactions that occur in partially-dealuminated zeolites to note IR, NMR, and EXAFS spectroscopic signatures of extra-framework Al in close proximity to Brønsted acid sites in H-ZSM-5 materials. Active centers with adjacent Brønsted acid sites and partially dislodged framework Al species showed higher rates for H_2_/D_2_ exchange and protolytic butane cracking and it was noted that accessible pore space in the zeolite was adjusted to accommodate alkane cracking transition states better leading to higher entropies of activation. The higher reactivity of H-FAU3.5 for protolytic alkane activation relative to H-FAU2.8 likely arises from similar tunability of pore size and space upon ion exchange.

## Discussion

Herein, we reported that a ML-based model, created using an in-house set of synthesis data, directed us to explore synthesis routes to enhance the Si/Al ratio of FAU zeolites via OSDA-free direct synthesis. Based on 81 training synthesis inputs and outcomes, the ML algorithm was validated with 7 testing points, and suggested synthesis conditions that elevated Si/Al to hitherto highest level (i.e., Si/Al = 3.5). Compared to a previously reported high-silica FAU zeolite (H-FAU2.8, entry 24) made by direct synthesis, H-FAU3.5 zeolite exhibits 2.5- and 2-fold increments in propane dehydrogenation and cracking rate constants per H^+^ site, respectively, demonstrating the potential of ML-directed synthesis to improve catalytic performance.

## Methods

### Na-FAU zeolites synthesis

The synthesis mixtures employed in this work were sodium aluminosilicates via template-free routes, and the molar compositions are listed in Supplementary Tables 1 and 2, in which the entries have different Si and Al sources. Specifically, Al sources include aluminum powder (99.9%, MilliporeSigma, abbreviated as Al powder), aluminum foil (99.999%, MilliporeSigma, abbreviated as Al foil), aluminium isopropoxide (98%, MilliporeSigma, abbreviated as Al(O-iPr)_3_), and sodium aluminate (Sigma-Aldrich, abbreviated as NaAl). Si sources include LUDOX HS-30 colloidal silica (abbreviated as HS30), LUDOX AS-40 colloidal silica (abbreviated as AS40), and sodium silicate (Sigma-Aldrich, abbreviated as NaSi). Sodium hydroxide solution (50% w/w, Neta Scientific) and deionized water are also used here for synthesis.

Two solutions were prepared during synthesis. Solution A (Si precursor solution) was prepared by adding a given amount of sodium hydroxide solution to a given amount of deionized water, followed by addition of a given amount of LUDOX HS-30 colloidal silica (or other Si precursors) into the prepared solution. Solution A formed a gel initially, and then it was heated in an oven at 343 K for 15–30 min until reaching a clear sol. Solution B (Al precursor solution) was prepared by adding a given amount of sodium hydroxide solution to a given amount of deionized water, followed by dissolving a given amount of aluminum powder (or other Al precursors) into the prepared solution (*Note: the reaction is exothermic and produces hydrogen, hence the addition of aluminum powder should be performed with caution and appropriate safety protocols in place*). Solutions A and B were cooled to ambient temperature and then solution B was added dropwise into solution A in a Teflon bottle while stirring. A freeze drying step is applied to remove water to a desired level (e.g., H_2_O_final_/H_2_O_initial_ = 0.47) within a lyophilizer at ambient temperature with a pressure of 20 mTorr. The synthesis mixture was aged with stirring at ambient temperature for 24 h, and then the vessel was heated in a static oven at a given temperature for a given duration (see details in Supplementary Tables 1 and 2). The products were then washed by repetitive centrifugation and redispersion by deionized water until the pH dropped to 9–10, and then they were dried at 343 K overnight.

### H-FAU zeolites preparation

Na-FAU zeolites were ion-exchanged with aqueous ammonium nitrate solutions for 1 h at ambient temperature. Each time 0.25 g of Na-FAU material was added to 40 cm^3^ of ammonium nitrate solution with stirring, and the ammonium concentrations were selected as **1** **M**. The solid products were thoroughly washed with deionized water at ambient temperature and then dried at 343 K for 6 h. Finally, the solid products were heated under inert helium flowing gas (0.167 cm^3^ s^−1^, Matheson) from ambient temperature to 673 K with a ramping rate of 0.033 K s^−1^ and maintained at 673 K for 6 h. The resulting samples were denoted as H-FAU zeolites.

### Synchrotron X-ray diffraction

XRD patterns were collected at Beamline 17-BM at Advanced Photon Source, Argonne National Laboratory. Powder samples were crushed finely with a pestle and mortar and loaded into 0.8–1 mm diameter Kapton capillaries. The X-ray wavelength used was 0.45192–0.45228 Å. 2-D diffraction data were collected in transmission geometry by a PerkinElmer amorphous silicon flat panel detector, and then 2-D diffraction data were processed with software GSAS II^64^ to obtain conventional XRD plots of intensity vs. 2θ. All XRD patterns presented in Supplementary Figs. 1–23 are converted to a wavelength of 1.54059 Å (Cu Kα).

### Argon physisorption

Measurements were performed at 87.3 K using an automatic manometric sorption Analyzer (Quantachrome Instruments Autosorb iQ MP). Prior to adsorption measurements, the samples were outgassed at 573 K for 10 h under turbomolecular pump vacuum ( < 0.003 Torr). Cumulative pore volume curves were calculated from the isotherms by applying an advanced NLDFT method, which assumes that argon adsorption at 87 K occurs in spherical siliceous zeolite pores in the micropore range and cylindrical silica pores in the mesopore range^59^.

### Scanning electron microscopy (SEM)

SEM images for tested samples were acquired using a JEOL JSM-6500 scanning electron microscope operated at 5 kV. SEM specimens were prepared by suspension of the sample powder in ethanol by ultrasonication for 30 min, and then the solution was dropped onto the surface of a silicon chip and dried at room temperature.

### Transmission electron microscopy (TEM)

TEM images were taken using a Tecnai T12 microscope operated at 120 kV with a LaB 6 filament. The specimens were prepared by dispersing the sample powder in ethanol and ultrasonicating for 30 min, and then the solution was dropped onto a Formvar-coated Cu grid and dried at room temperature.

### Solid-state magic angle spinning (MAS) nuclear magnetic resonance (NMR) spectroscopy

^27^Al and ^29^Si MAS NMR spectra were acquired with a Bruker DSX-500 spectrometer (11.7 T magnet) and a 4 mm Bruker MAS probe. The spectral frequencies were 78.2 MHz and 99.4 MHz for the ^27^Al and ^29^Si nucleus, respectively.

### Infrared spectroscopy

Infrared (IR) spectra for pyridine adsorption were collected for H-FAU samples on a Nicolet™ iS50 Fourier transform infrared spectrometer with a Hg-Cd-Te (MCT, cooled to 77 K by liquid N_2_) detector by averaging 128 scans at 2 cm^−1^ resolution in the 600–4000 cm^−1^ range and were taken relative to an empty cell background reference collected under dynamic vacuum (~0.01 Torr) at 498 K. Self-supporting wafers (0.01–0.03 g cm^−2^, with a diameter of 13 mm) were sealed within an IR transmission cell with ZnSe windows (High Temperature Transmission Cell, Harrick Scientific Products Inc.). Wafer temperatures were measured by K-type thermocouples (Omega) attached to the sample holder. The IR cell was connected to a glass vacuum manifold, which was used for sample exposure to controlled amounts of gaseous pyridine. The temperature program followed for these measurements is described herein: sample dehydration was performed initially, the temperature of the cell was initially raised from ambient temperature to 673 K at a ramping rate of 0.033 Ks^−1^ followed by holding temperature at 673 K for 6 h; then the temperature was cooled down to 498 K and pyridine was introduced until saturation of the adsorbate was noted with invariance among successive spectra recorded.

### Catalytic tests

Proton-catalyzed monomolecular propane reactions were performed in a tubular glass-lined stainless steel reactor (6.35 mm O.D. and 4 mm I.D., SGE Analytical Science) equipped with a thermocouple to monitor the reaction temperature. The catalyst sample was heated in helium flow (0.083 cm^3^ s^−1^, Matheson) from ambient temperature to the reaction temperature at atmospheric pressure. Prior to data acquisition, we pretreated samples using H_2_/He mixtures (*p*_H2_ = 35 kPa, H_2_/He = 1:2, and the total flow rate = 0.5 cm^3^ s^−1^) for 20 min to remove any remnant reactive carbon species. Molar ratios of feed gas mixtures were fixed as H_2_/C_3_H_8_/Ar/He = 3/3/1.5/60 with Ar serving as an internal standard, and space velocity of 3600 cm^3^_C3H8_·g_cat_^−1^ h^−1^ with a total flow rate of 1.125 cm^3^ s^−1^. H_2_ is present in the inlet stream to mitigate on-stream deposition of organic species. Reactor effluent was vented to atmospheric pressure, system pressure varied from 101 kPa to 120 kPa as measured by a PX209-300G5V pressure transducer. Reactor temperature varied from 818 to 893 K with an interval of 15 K. Propane conversions were <1% and considered differential for assessment of catalytic rates. The composition of the reactor effluent was analyzed by an online Agilent 7890 A gas chromatograph (GC) using a flame ionization detector (FID) and a thermal conductivity detector (TCD). Eluent separation was achieved in parallel using a dimethylpolysiloxane J&W HP-1 column (50 m long, 320 μm diameter, 0.52 μm film thickness) connected to the FID and a GS-GasPro (60 m long, 320 μm diameter) preceding the TCD. Ar was quantified using the TCD, and all hydrocarbon species were quantified using the FID.

### Reporting summary

Further information on research design is available in the Nature Portfolio Reporting Summary linked to this article.

## Supplementary information

Supplementary Information

Reporting Summary

## Data Availability

The data that support the findings of this study are provided in Supplementary Information and Source Data file. Source Data are provided as a Source Data file and enclosed with this paper. Details listed in the Supplementary Information consist of the synthesis procedures, characterization results (XRD patterns, Ar-adsorption isotherms, SEM/TEM images, ^29^Si solid-state NMR), reactivity analysis, and machine learning methods and results. Source data are provided with this paper.

## Source data

Source Data File

## Code availability

The codes used to train the Machine learning models can be accessed in the public Gitlab repository (https://gitlab.com/nicolasevangelou/zeolites_ml.git) and the Figshare Dataset.
