# Supplementary material for: Machine learning-assisted crystal engineering of a zeolite
Source: Nat Commun. 2023 May 31;14:3152. doi: 10.1038/s41467-023-38738-5 (PMC10232492; doi:10.1038/s41467-023-38738-5)
Supplement: Supplementary file 1 — Supplementary Information [file 41467_2023_38738_MOESM1_ESM.pdf]

Supplementary Information

Machine Learning-Assisted Crystal Engineering of a Zeolite

Xinyu Li[1,9], He Han[1,2,9], Nikolaos Evangelou[3,9], Noah J. Wichrowski[4,9], Peng Lu[3], Wenqian Xu[5], Son-Jong Hwang[6], Wenyang Zhao[1], Chunshan Song[2], Xinwen Guo[2], Aditya Bhan[1,*], Ioannis G. Kevrekidis[3,4,*], and Michael Tsapatsis[1,3,7,8,*]

[1]Department of Chemical Engineering and Materials Science, University of Minnesota, 421 Washington Avenue SE, Minneapolis, Minnesota 55455, United States

[2]State Key Laboratory of Fine Chemicals, PSU-DUT Joint Center for Energy Research, School of Chemical Engineering, Dalian University of Technology, Dalian, 116024, Liaoning Province, China

[3]Department of Chemical and Biomolecular Engineering, Johns Hopkins University, 3400 North Charles Street, Baltimore, Maryland 21218, United States

[4]Department of Applied Mathematics and Statistics, Johns Hopkins University, 3400 North Charles Street, Baltimore, Maryland 21218, United States

[5]X-ray Science Division, Advanced Photon Source, Argonne National Laboratory, Lemont, Illinois 60439, United States

[6]Division of Chemistry and Chemical Engineering, California Institute of Technology, Pasadena, California 91125, United States

[7]Applied Physics Laboratory, Johns Hopkins University, 11100 Johns Hopkins Road, Laurel, Maryland 20723, United States

[8]Institute for NanoBioTechnology, Johns Hopkins University, 3400 North Charles Street, Baltimore, Maryland 21218, United States

[9]These authors contributed equally: Xinyu Li, He Han, Nikolaos Evangelou, Noah J. Wichrowski.

*Corresponding Author: abhan@umn.edu; yannisk@jhu.edu; tsapatsis@jhu.edu

Contents

S1. Experimental Recipes

S2. Characterization Results of Faujasite Zeolites

S3. Method Description of Machine Learning

S4. Correlations of Experimental and Predicted Physical Properties of Faujasite Zeolites

S5. Supporting Information for Framework Si/Al ratio, Infrared Spectra and Reactivity of Faujasite Zeolites

S1. Experimental Recipes

Supplementary Figure 1 shows all experiments in plots of H_2O_{final}/Na_2O versus Na_2O/Al_2O_3 for different SiO_2/Al_2O_3 ratios (SiO_2/Al_2O_3 = 12, < 12, 14, and > 12 (other than 14) in Supplementary Figure 1 (a)~(d), respectively), and reflects the two $H_2O_{final}/H_2O_{initial}$ levels of 1 and 0.47. Entry number is listed in Supplementary Figure 1, and entry details are provided in the Supporting Information (Supplementary Tables 1 and 2). Entries A1-A86 contain other zeolites (GIS, SOD, etc.) or amorphous phases instead of pure faujasite; entries 1-88 are pure faujasite zeolites (FAU or FAU/EMT) used for ML algorithm training (represented in blue) and testing (represented in red).

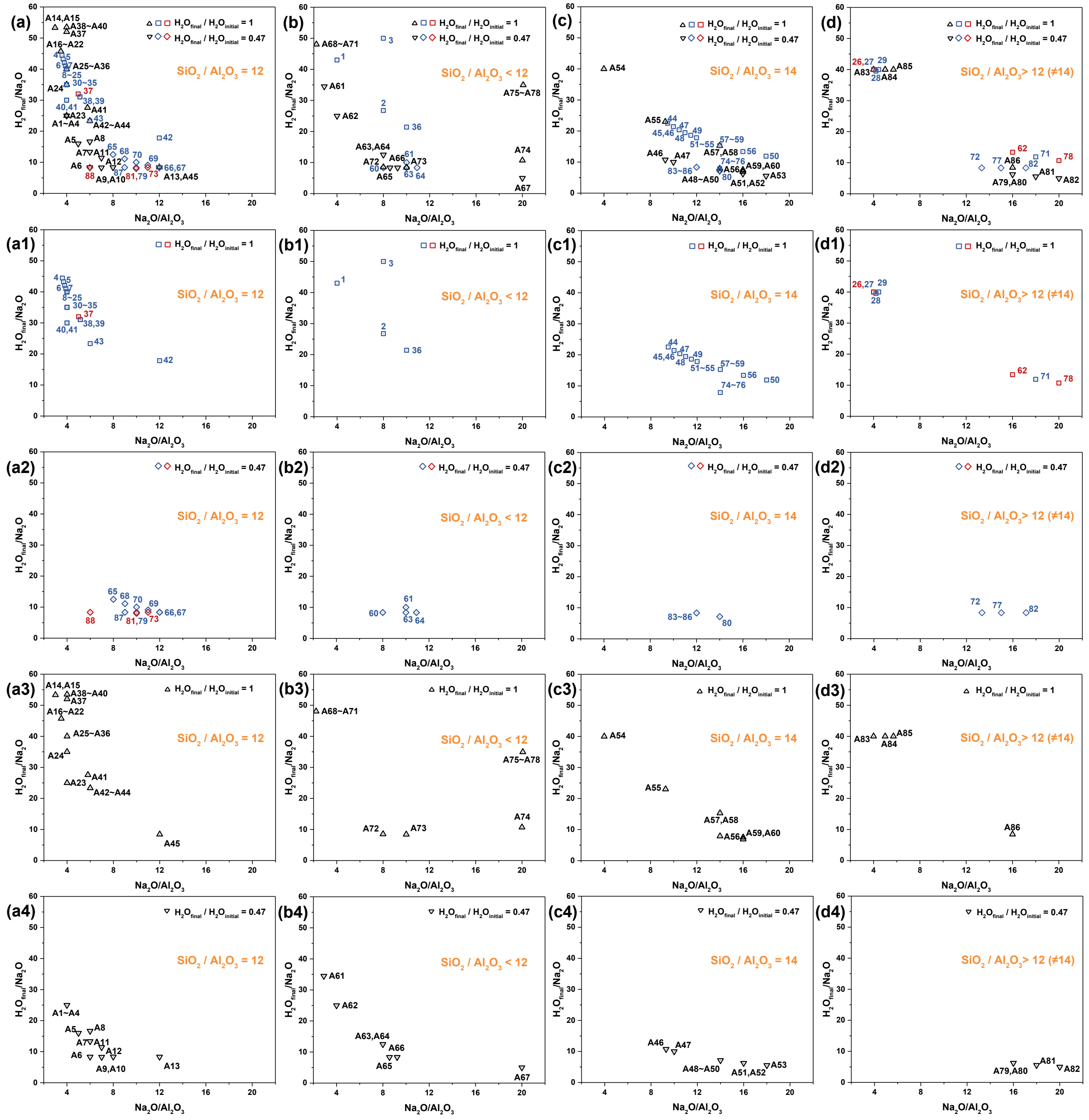

SiO$_2$ / Al$_2$O$_3$ < 12
= 3.4 for entry **1**
= 4 for entries **A61~A63**, **A67**, **A74**
= 5 for entries **A75~A78**
= 8 for entries **2**, **60**, **A64**, **A68~A72**
= 8.6 for entry **A65**
= 9.2 for entry **A66**
= 10 for entries **3**, **36**, **61**, **63**, **A73**
= 10.9 for entry **64**

SiO$_2$ / Al$_2$O$_3$ > 12 (≠14)
= 12.5 for entries **26**, **27**, **28**
= 13.3 for entries **29**, **72**
= 15 for entries **77**, **A83**, **A84**
= 16 for entries **62**, **A79**, **A80**, **A86**
= 17.1 for entries **82**, **A85**
= 18 for entries **71**, **A81**
= 20 for entries **78**, **A82**

Supplementary Figure 1. Explored region in composition space aiming at pure FAU and FAU/EMT zeolite synthesis. x- and y- axes are Na_2O/Al_2O_3 (input), H_2O_{final}/Na_2O (input), respectively. The 174 (88 + 86) synthesis experiments can be divided into three groups based on the SiO_2/Al_2O_3 ratio of the synthesis mixture: (a) SiO_2/Al_2O_3 = 12, (b) SiO_2/Al_2O_3 < 12, (c) SiO_2/Al_2O_3 = 14, and (d) SiO_2/Al_2O_3 > 12 (other than 14). Square (□) and diamond (◇) points (in blue and red color, see

below for explanation of distinction based on color) represent pure faujasite zeolites (FAU and FAU/EMT) without ($H_2O_{final}/H_2O_{initial}$ = 1) and with ($H_2O_{final}/H_2O_{initial}$ = 0.47) the freeze drying step, respectively. Black up (△) and down (▽) triangle points represent tried recipes leading to other zeolites (GIS, SOD, etc.) or amorphous phases. Figs. 1(a)~(d) are divided into (a1)(a2)(a3)(a4)~(d1)(d2)(d3)(d4) subplots based on product phases and ratio of $H_2O_{final}/H_2O_{initial}$. Entry numbers are listed in the figures, and entry details are provided in the Supporting Information (Supplementary Tables 1 and 2). Entries A1-A86 contain other zeolites (GIS, SOD, etc.) or amorphous phases instead of pure faujasite; entries 1-88 are pure faujasite zeolites (FAU or FAU/EMT) used for ML algorithm training (represented in blue) and testing (represented in red).

Supplementary Table 1. Synthesis recipes and corresponding physical properties of faujasite zeolite (FAU and EMT) materials.

Entry	SiO_2	Al_2O_3	Na_2O	$H_2O_{initial}$	H_2O_{final}	Si source	Al source	Type of oven	Cryst. Temp. (°C)	Cryst. Time (day)	Si/Al ratio via ICP	Particle Size (nm)	Crystal Size (nm)	FAU/ (FAU + EMT)	uptake value at p/p_0= 0.01 (cm^3/g)
1	3.40	1.00	4.00	172.00	172.00	NaSi	NaAl	static oven	50	6.00	1.281	4219	103.8	0.993	215
2	8.00	1.00	8.00	214.00	214.00	NaSi	NaAl	static oven	50	6.00	1.343	81	17.1	0.705	182
3	10.00	1.00	8.00	400.00	400.00	HS30	Al powder	static oven	100	0.75	1.633	3150	113.9	0.992	197
4	12.00	1.00	3.60	160.00	160.00	AS40	NaAl	static oven	100	7.00	2.838	1678	88.3	0.993	196
5	12.00	1.00	3.70	160.00	160.00	AS40	NaAl	static oven	100	7.00	2.793	1659	85.1	0.994	208
6	12.00	1.00	3.80	160.00	160.00	AS40	NaAl	static oven	100	5.00	2.772	1643	86.5	0.991	209
7	12.00	1.00	3.90	160.00	160.00	AS40	NaAl	static oven	100	5.00	2.693	1468	89.9	0.990	214
8	12.00	1.00	4.00	160.00	160.00	AS40	NaAl	static oven	100	3.00	2.651	1516	89.9	0.989	214
9	12.00	1.00	4.00	160.00	160.00	AS40	NaAl	static oven	100	3.00	2.669	1570	89.9	0.995	219
10	12.00	1.00	4.00	160.00	160.00	AS40	NaAl	static oven	100	3.00	2.688	1580	85.1	0.993	224
11	12.00	1.00	4.00	160.00	160.00	AS40	Al powder	static oven	100	3.00	2.709	1900	85.4	0.994	213
12	12.00	1.00	4.00	160.00	160.00	AS40	NaAl	static oven	100	3.00	2.679	1560	88.3	0.993	190
13	12.00	1.00	4.00	160.00	160.00	AS40	NaAl	static oven	100	3.00	2.669	1480	86.5	0.994	212
14	12.00	1.00	4.00	160.00	160.00	AS40	NaAl	static oven	100	3.00	2.694	1254	82.1	0.994	198
15	12.00	1.00	4.00	160.00	160.00	AS40	Al powder	static oven	100	3.00	2.720	1477	85.1	0.994	208
16	12.00	1.00	4.00	160.00	160.00	HS30	Al powder	static oven	100	3.00	2.677	1798	86.5	0.994	216
17	12.00	1.00	4.00	160.00	160.00	AS40	NaAl	static oven	100	5.00	2.697	1579	84.8	0.999	209
18	12.00	1.00	4.00	160.00	160.00	AS40	NaAl	static oven	100	5.00	2.683	1400	85.0	0.994	234
19	12.00	1.00	4.00	160.00	160.00	AS40	Al powder	static oven	100	5.00	2.712	2160	Not collected	Not collected	226
20	12.00	1.00	4.00	160.00	160.00	AS40	NaAl	static oven	100	5.00	2.695	1293	85.1	0.992	197
21	12.00	1.00	4.00	160.00	160.00	HS30	Al powder	rotation oven	100	5.00	2.770	3300	47.0	0.961	201
22	12.00	1.00	4.00	160.00	160.00	AS40	NaAl	static oven	100	6.00	2.655	1470	Not collected	Not collected	212
23	12.00	1.00	4.00	160.00	160.00	AS40	Al powder	static oven	100	7.00	2.729	1197	80.2	0.998	209
24	12.00	1.00	4.00	160.00	160.00	AS40	NaAl	static oven	100	7.00	2.762	1200	82.4	0.998	196
25	12.00	1.00	4.00	160.00	160.00	AS40	Al powder	static oven	100	9.00	2.764	1368	80.6	0.993	201
26	12.50	1.00	4.00	160.00	160.00	AS40	Al powder	static oven	100	3.00	2.758	2530	83.4	0.991	168
27	12.50	1.00	4.00	160.00	160.00	AS40	Al powder	static oven	100	8.00	2.635	2170	Not collected	Not collected	212
28	12.50	1.00	4.20	166.00	166.00	AS40	NaAl	static oven	100	3.00	2.514	1657	86.5	0.989	205
29	13.33	1.00	4.44	177.78	177.78	AS40	NaAl	static oven	100	5.00	2.593	1803	16.2	0.992	220
30	12.00	1.00	4.00	140.00	140.00	AS40	NaAl	static oven	100	3.00	2.580	1340	82.6	0.996	222
31	12.00	1.00	4.00	140.00	140.00	AS40	Al powder	static oven	100	3.00	2.577	1604	82.1	0.993	178
32	12.00	1.00	4.00	140.00	140.00	AS40	Al powder	static oven	100	5.00	2.575	1741	85.6	0.991	216

Entry	SiO_2	Al_2O_3	Na_2O	$H_2O_{initial}$	H_2O_{final}	Si source	Al source	Type of oven	Cryst. Temp. (°C)	Cryst. Time (day)	Si/Al ratio via ICP	Particle Size (nm)	Crystal Size (nm)	FAU/ (FAU + EMT)	uptake value at p/p_0= 0.01 (cm^3/g)
33	12.00	1.00	4.00	140.00	140.00	AS40	Al powder	static oven	100	5.00	2.561	1497	79.3	0.992	208
34	12.00	1.00	4.00	140.00	140.00	AS40	Al powder	static oven	100	7.00	2.558	1340	80.8	0.992	208
35	12.00	1.00	4.00	140.00	140.00	AS40	Al powder	static oven	100	7.00	2.683	1764	85.1	0.992	213
36	10.00	1.00	10.00	214.00	214.00	NaSi	NaAl	static oven	50	6.00	1.288	75	13.5	0.499	142
37	12.00	1.00	5.00	160.00	160.00	AS40	Al powder	static oven	100	3.00	2.251	876	83.4	0.993	208
38	12.00	1.00	5.15	160.00	160.00	AS40	NaAl	static oven	100	3.00	2.158	870	83.9	0.997	214
39	12.00	1.00	5.15	160.00	160.00	AS40	NaAl	static oven	100	5.00	2.150	880	82.9	0.998	211
40	12.00	1.00	4.00	120.00	120.00	AS40	Al powder	static oven	100	3.00	2.669	1870	80.8	0.993	208
41	12.00	1.00	4.00	120.00	120.00	AS40	NaAl	static oven	100	3.00	2.614	1700	78.1	0.992	108
42	12.00	1.00	12.00	214.00	214.00	NaSi	NaAl	static oven	50	6.00	1.266	161	13.5	0.469	110
43	12.00	1.00	6.00	140.00	140.00	HS30	Al powder	rotation oven	90	4.00	1.886	904	82.5	0.989	171
44	14.00	1.00	9.50	214.00	214.00	NaSi	NaAl	static oven	65	2.00	1.814	1200	57.2	0.954	175
45	14.00	1.00	10.00	214.00	214.00	NaSi	NaAl	static oven	65	2.00	1.593	560	43.8	0.892	185
46	14.00	1.00	10.00	214.00	214.00	NaSi	NaAl	static oven	50	6.00	1.709	293	25.2	0.876	177
47	14.00	1.00	10.50	214.00	214.00	NaSi	NaAl	static oven	65	2.00	1.493	239	25.2	0.801	204
48	14.00	1.00	11.00	214.00	214.00	NaSi	NaAl	static oven	65	2.00	1.479	186	25.0	0.804	191
49	14.00	1.00	11.50	214.00	214.00	NaSi	NaAl	static oven	65	2.00	1.430	193	23.7	0.783	163
50	14.00	1.00	18.00	214.00	214.00	NaSi	NaAl	static oven	50	6.00	1.088	846	40.6	0.863	170
51	14.00	1.00	12.00	214.00	214.00	NaSi	NaAl	oil bath	50	2.00	1.419	94	16.2	0.712	192
52	14.00	1.00	12.00	214.00	214.00	HS30	NaAl	oil bath	50	2.00	1.516	92	18.9	0.749	114
53	14.00	1.00	12.00	214.00	214.00	HS30	Al foil	oil bath	50	2.00	1.398	141	21.0	0.787	113
54	14.00	1.00	12.00	214.00	214.00	NaSi	NaAl	static oven	50	6.00	1.442	481	20.3	0.767	173
55	14.00	1.00	12.00	214.00	214.00	NaSi	NaAl	static oven	65	2.00	1.301	254	32.5	0.863	192
56	14.00	1.00	16.00	214.00	214.00	NaSi	NaAl	static oven	50	6.00	1.167	323	16.8	0.618	175
57	14.00	1.00	14.00	214.00	214.00	NaSi	NaAl	static oven	50	6.00	1.281	253	18.6	0.679	129
58	14.00	1.00	14.00	214.00	214.00	NaSi	NaAl	static oven	65	2.00	1.281	200	13.0	0.432	125
59	14.00	1.00	14.00	214.00	214.00	NaSi	NaAl	static oven	65	2.00	1.153	193	49.2	0.887	146
60	8.00	1.00	8.00	142.67	66.67	HS30	Al powder	rotation oven	50	6.00	1.202	16	14.4	0.447	38
61	10.00	1.00	10.00	214.00	100.00	NaSi	NaAl	static oven	50	6.00	1.160	36	17.0	0.589	170
62	16.00	1.00	16.00	214.00	214.00	NaSi	NaAl	static oven	50	6.00	1.244	660	49.3	0.828	138
63	10.00	1.00	10.00	178.33	83.33	HS30	Al powder	rotation oven	50	6.00	1.150	19	18.1	0.753	170
64	10.91	1.00	10.91	194.55	90.91	HS30	Al powder	rotation oven	50	6.00	1.065	22	17.3	0.850	224
65	12.00	1.00	8.00	214.00	100.00	HS30	Al powder	rotation oven	50	6.00	1.759	161	26.4	0.807	124
66	12.00	1.00	12.00	214.00	100.00	HS30	Al powder	rotation oven	50	5.00	1.190	29	21.5	0.865	220
67	12.00	1.00	12.00	214.00	100.00	NaSi	NaAl	static oven	50	6.00	1.232	32	21.6	0.834	162
68	12.00	1.00	9.00	214.00	100.00	HS30	Al powder	rotation oven	50	6.00	1.513	147	26.0	0.808	130
69	12.00	1.00	11.00	214.00	100.00	HS30	Al powder	rotation oven	50	6.00	1.179	72	20.1	0.774	159
70	12.00	1.00	10.00	214.00	100.00	HS30	Al powder	rotation oven	50	6.00	1.291	61	14.7	0.652	118
71	18.00	1.00	18.00	214.00	214.00	NaSi	NaAl	static oven	50	6.00	1.311	605	67.2	0.957	182
72	13.33	1.00	13.33	237.78	111.11	HS30	Al powder	rotation oven	50	6.00	1.103	36	29.0	0.856	192

Entry	SiO_2	Al_2O_3	Na_2O	$H_2O_{initial}$	H_2O_{final}	Si source	Al source	Type of oven	Cryst. Temp. (°C)	Cryst. Time (day)	Si/Al ratio via ICP	Particle Size (nm)	Crystal Size (nm)	FAU/ (FAU + EMT)	uptake value at p/p_0= 0.01 (cm^3/g)
73	12.00	1.00	11.00	196.00	92.00	HS30	Al powder	rotation oven	50	5.00	1.204	91	19.3	0.825	135
74	14.00	1.00	14.00	110.00	110.00	NaSi	NaAl	oil bath	50	2.00	1.196	102	31.3	0.889	181
75	14.00	1.00	14.00	110.00	110.00	NaSi	NaAl	oil bath	50	3.00	1.162	372	56.9	0.982	189
76	14.00	1.00	14.00	110.00	110.00	NaSi	NaAl	oil bath	50	3.00	1.159	204	39.9	0.928	143
77	15.00	1.00	15.00	267.50	125.00	HS30	Al powder	rotation oven	50	6.00	1.174	85	49.4	0.986	209
78	20.00	1.00	20.00	214.00	214.00	NaSi	NaAl	static oven	50	6.00	1.255	475	86.5	0.993	208
79	12.00	1.00	10.00	178.00	83.00	HS30	Al powder	rotation oven	50	5.00	1.245	168	19.6	0.83	114
80	14.00	1.00	14.00	214.00	100.00	NaSi	NaAl	static oven	50	6.00	1.207	150	60.7	0.993	194
81	12.00	1.00	10.00	180.00	80.00	HS30	Al powder	rotation oven	50	4.00	1.308	40	24.5	0.859	196
82	17.14	1.00	17.14	305.71	142.86	HS30	Al powder	rotation oven	50	6.00	1.197	608	53.7	0.916	157
83	14.00	1.00	12.00	214.00	100.00	NaSi	NaAl	oil bath	50	2.00	1.288	103	33.9	0.891	211
84	14.00	1.00	12.00	214.00	100.00	HS30	NaAl	oil bath	50	2.00	1.391	146	50.2	0.955	176
85	14.00	1.00	12.00	214.00	100.00	HS30	Al foil	oil bath	50	2.00	1.345	146	39.6	0.939	163
86	14.00	1.00	12.00	214.00	100.00	NaSi	NaAl	static oven	50	6.00	1.310	70	31.8	0.889	169
87	12.00	1.00	9.00	160.00	75.00	HS30	Al powder	rotation oven	50	5.00	2.032	392	80.6	0.99	145
88	12.00	1.00	6.00	107.00	50.00	HS30	Al powder	static oven	70	4.00	2.289	300	57.6	0.985	172
89	12.00	1.00	3.50	160.00	160.00	AS40	NaAl	static oven	100	9.00	3.016	1347	91.8	0.993	214
90	12.00	1.00	3.50	160.00	160.00	AS40	NaAl	static oven	100	12.00	3.018	1350	89.9	0.992	216
91	12.00	1.00	3.50	160.00	160.00	AS40	NaAl	static oven	100	13.00	3.528	1808	92.5	0.991	157
92	13.00	1.00	3.00	160.00	160.00	NaSi	NaAl	static oven	100	13.00	3.150	1685	83.9	0.995	179

Note: Blue entries represent training points, red entries represent testing points, and green entries represent prediction points.

Supplementary Table 2. Synthesis recipes leading to other zeolites or amorphous phases.

Entry	SiO_2	Al_2O_3	Na_2O	$H_2O_{initial}$	H_2O_{final}	Si source	Al source	Type of oven	Cryst. Temp. (°C)	Cryst. Time (day)	Phase
A1	12	1	4	214	100	AS40	NaAl	static oven	100	1.00	Amorphous
A2	12	1	4	214	100	AS40	NaAl	static oven	100	3.00	Amorphous
A3	12	1	4	214	100	AS40	NaAl	static oven	100	5.00	FAU+GIS
A4	12	1	4	214	100	AS40	NaAl	static oven	100	7.00	Amorphous
A5	12	1	5	180	80	HS30	Al powder	rotation oven	50	4.00	Amorphous
A6	12	1	6	107	50	HS30	Al powder	rotation oven	50 (step1) + 70 (step 2)	5.00 (step 1) + 3.00 (step 2)	FAU+GIS
A7	12	1	6	180	80	HS30	Al powder	rotation oven	50	4.00	Amorphous
A8	12	1	6	214	100	HS30	Al powder	rotation oven	50 (step 1) + 65 (step 2)	6.00 (step 1) + 5.00 (step 2)	Zeolite LZ-276
A9	12	1	7	125	58	HS30	Al powder	rotation oven	50	5.00	Amorphous
A10	12	1	7	125	58	HS30	Al powder	rotation oven	50 (step 1) + 70 (step 2)	5.00 (step 1) + 3.00 (step 2)	FAU + GIS
A11	12	1	7	180	80	HS30	Al powder	rotation oven	50	4.00	Amorphous
A12	12	1	8	143	67	HS30	Al powder	rotation oven	50	5.00	FAU + GIS
A13	12	1	12	214	100	HS30	Al powder	rotation oven	50	7.00	FAU + SOD
A14	12	1	3	160	160	AS40	NaAl	static oven	100	3.00	Amorphous
A15	12	1	3	160	160	AS40	Al powder	static oven	100	3.00	Amorphous
A16	12	1	3.5	160	160	AS40	NaAl	static oven	120	3.00	FAU + LTA
A17	12	1	3.5	160	160	AS40	NaAl	static oven	120	5.00	FAU + LTA
A18	12	1	3.5	160	160	AS40	NaAl	static oven	120	7.00	FAU + LTA
A19	12	1	3.5	160	160	AS40	NaAl	static oven	120	2.00	Amorphous + FAU
A20	12	1	3.5	160	160	AS40	NaAl	static oven	100	7.00	Amorphous (FAU)
A21	12	1	3.5	160	160	AS40	NaAl	static oven	120	1.00	Amorphous
A22	12	1	3.5	160	160	AS40	Al powder	static oven	100	3.00	Amorphous
A23	12	1	4	100	100	AS40	Al powder	static oven	100	3.00	FAU + Amorphous
A24	12	1	4	140	140	AS40	Al powder	static oven	100	3.00	FAU + Amorphous
A25	12	1	4	160	160	AS40	NaAl	static oven	100	3.00	Faulted CHA
A26	12	1	4	160	160	AS40	NaAl	static oven	100	3.00	Faulted CHA
A27	12	1	4	160	160	AS40	NaAl	static oven	100	3.00	Faulted CHA
A28	12	1	4	160	160	AS40	NaAl	static oven	100	2.00	FAU + Amorphous
A29	12	1	4	160	160	AS40	NaAl	static oven	100	2.00	FAU + Amorphous
A30	12	1	4	160	160	AS40	Al powder	static oven	100	5.00	FAU + Amorphous.
A31	12	1	4	160	160	AS40	NaAl	rotation oven	100	3.00	Amorphous + FAU
A32	12	1	4	160	160	AS40	Al powder	rotation oven	100	3.00	Amorphous
A33	12	1	4	160	160	Silicic acid	NaAl	static oven	100	3.00	Amorphous
A34	12	1	4	160	160	Silicic acid	Al powder	static oven	100	3.00	Amorphous
A35	12	1	4	160	160	AS40	$Al(O\text{-}iPr)_3$	static oven	100	3.00	Amorphous
A36	12	1	4	160	160	HS30	$Al(O\text{-}iPr)_3$	static oven	100	3.00	Amorphous

Entry	SiO_2	Al_2O_3	Na_2O	$H_2O_{initial}$	H_2O_{final}	Si source	Al source	Type of oven	Cryst. Temp. (°C)	Cryst. Time (day)	Phase
A37	12	1	4	208	208	fumed silica	Al powder	static oven	100	3.00	Amorphous (FAU)
A38	12	1	4	214	214	AS40	NaAl	static oven	100	3.00	FAU + GIS
A39	12	1	4	214	214	AS40	NaAl	static oven	100	1.00	Amorphous
A40	12	1	4	214	214	AS40	NaAl	static oven	100	5.00	Amorphous
A41	12	1	5.8	160	160	NaSi	NaAl	static oven	100	3.00	GIS
A42	12	1	6	140	140	NaSi	NaAl	static oil bath	90	2.00	GIS
A43	12	1	6	140	140	NaSi	NaAl	static oil bath	90	2.30	GIS
A44	12	1	6	140	140	NaSi	NaAl	static oil bath	90	2.00	Amorphous
A45	12	1	12	101	101	NaSi	NaAl	static oven	50	6.00	FAU/EMT + SOD
A46	14	1	9.3	214	100	NaSi	NaAl	static oven	65	2.00	GIS
A47	14	1	10	214	100	NaSi	NaAl	static oven	50	6.00	Amorphous + FAU
A48	14	1	14	214	100	NaSi	NaAl	oil bath	50	6.00	FAU + SOD
A49	14	1	14	214	100	HS30	NaAl	oil bath	50	6.00	FAU + SOD
A50	14	1	14	214	100	AS40	NaAl	oil bath	50	6.00	FAU + SOD
A51	14	1	16	214	100	NaSi	NaAl	rotation oven	50	6.00	SOD
A52	14	1	16	214	100	NaSi	NaAl	static oven	50	6.00	FAU + SOD
A53	14	1	18	214	100	NaSi	NaAl	static oven	50	6.00	SOD
A54	14	1	4	160	160	AS40	NaAl	static oven	100	10.00	FAU + CHA
A55	14	1	9.3	214	214	NaSi	NaAl	static oven	65	2.00	FAU + GIS
A56	14	1	14	110	110	NaSi	NaAl	static oil bath	50	1.25	FAU + SOD
A57	14	1	14	214	214	NaSi	NaAl	flow synthesis	50	0.50	FAU/EMT + Amorphous
A58	14	1	14	214	214	NaSi	NaAl	static oven	50	3.00	FAU/EMT + Amorphous
A59	14	1	16	110	110	NaSi	NaAl	static oil bath	50	2.00	SOD
A60	14	1	16	120	120	NaSi	NaAl	static oven	50	6.00	SOD
A61	4	1	2.9	214	100	NaSi	NaAl	static oven	65	2.00	FAU + GIS
A62	4	1	4	214	100	NaSi	NaAl	static oven	65	2.00	FAU/EMT
A63	4	1	8	214	100	NaSi	NaAl	static oven	65	2.00	SOD
A64	8	1	8	214	100	NaSi	NaAl	static oven	50	6.00	Amorphous + EMT
A65	8.6	1	8.6	153	71	HS30	Al powder	room temp	50	6.00	FAU/EMT + SOD
A66	9.2	1	9.2	165	77	HS30	Al powder	room temp	50	6.00	FAU/EMT + SOD
A67	4	1	20	214	100	NaSi	NaAl	static oven	65	2.00	SOD
A68	8	1	2.22	107	107	AS40	NaAl	static oven	100	9.00	FAU + LTA
A69	8	1	2.22	107	107	AS40	NaAl	static oven	100	7.00	FAU (LTA)
A70	8	1	2.22	107	107	AS40	NaAl	static oven	100	3.00	FAU (LTA)
A71	8	1	2.22	107	107	AS40	NaAl	static oven	100	1.00	Amorphous
A72	8.6	1	8.6	153	71	HS30	Al powder	rotation oven	50	6.00	FAU/EMT + SOD

Entry	SiO_2	Al_2O_3	Na_2O	$H_2O_{initial}$	H_2O_{final}	Si source	Al source	Type of oven	Cryst. Temp. (°C)	Cryst. Time (day)	Phase
A73	10	1	10	84	84	NaSi	NaAl	static oven	50	6.00	EMT + SOD
A74	4	1	20	214	214	NaSi	NaAl	static oven	65	2.00	SOD
A75	5	1	20.1	702	702	NaSi	NaAl	static oven	50	1.00	Amorphous
A76	5	1	20.1	702	702	NaSi	NaAl	static oven	50	3.00	Amorphous
A77	5	1	20.1	702	702	NaSi	NaAl	static oven	50	2.00	Amorphous
A78	5	1	20.1	702	702	NaSi	NaAl	static oven	50	2.50	Amorphous
A79	16	1	16	214	100	NaSi	NaAl	rotation oven	50	6.00	SOD
A80	16	1	16	214	100	NaSi	NaAl	static oven	50	6.00	Amorphous
A81	18	1	18	214	100	NaSi	NaAl	static oven	50	6.00	SOD
A82	20	1	20	214	100	NaSi	NaAl	static oven	50	6.00	Amorphous
A83	15	1	4	160	160	AS40	NaAl	static oven	100	10.00	Amorphous
A84	15	1	5	200	200	AS40	NaAl	static oven	100	5.00	FAU + Amorphous
A85	17.1	1	5.7	229	229	AS40	NaAl	static oven	100	5.00	Amorphous
A86	16	1	16	135	135	NaSi	NaAl	static oven	50	6.00	FAU/EMT + SOD

Note: Entries A1-A86 contain other zeolites (GIS, SOD, etc.) or amorphous phases instead of pure faujasite, and they are shown as black up (△) and down (▽) triangle points in Fig. 1 from the main text.

S2. Characterization Results of Faujasite Zeolites

Details of characterization results of FAU zeolites are provided in Supplementary Tables 3 ~ 25 and Supplementary Figures 2 ~ 24, including Ar-adsorption isotherms, XRD patterns, SEM images, ICP analysis. Si/Al ratio is measured via ICP analysis. Particle size is measured from SEM/TEM images. Crystal size is measured via XRD patterns in accordance with the Scherrer equation averaged for FAU(311) and FAU(331) reflections (only entries with Scherrer particle size < 60 nm were considered for the ML analysis to avoid error introduced by instrumental broadening, no correction for instrumental broadening was applied). FAU fraction (FAU/(FAU+EMT) is estimated via deconvolution of the first main diffraction peak in XRD patterns, namely the peak area ratio of FAU(111) / (FAU(111) + EMT(100)). Uptake values at P/P_0 = 0.01 refer to a single point in the Ar-adsorption isotherms. Log_{10}(Particle size / Crystal size) can be used to describe the degree of aggregation for FAU particles, lower values of this parameter imply lower aggregation (i.e., a value of zero corresponds to single-crystal FAU nanoparticles).

Supplementary Table 3. Synthesis recipes and corresponding physical properties of faujasite zeolite (FAU and EMT) materials (Entries 1~4).

Entry	SiO_2	Al_2O_3	Na_2O	$H_2O_{initial}$	H_2O_{final}	Si source	Al source	Type of oven	Cryst. Temp. (°C)	Cryst. Time (day)	Si/Al ratio via ICP	Particle Size (nm)	Crystal Size (nm)	FAU/ (FAU + EMT)	uptake value at p/p_0= 0.01 (cm^3/g)
1	3.40	1.00	4.00	172.00	172.00	NaSi	NaAl	static oven	50	6.00	1.281	4219	103.8	0.993	215
2	8.00	1.00	8.00	214.00	214.00	NaSi	NaAl	static oven	50	6.00	1.343	81	17.1	0.705	182
3	10.00	1.00	8.00	400.00	400.00	HS30	Al powder	static oven	100	0.75	1.633	3150	113.9	0.992	197
4	12.00	1.00	3.60	160.00	160.00	AS40	NaAl	static oven	100	7.00	2.838	1678	88.3	0.993	196

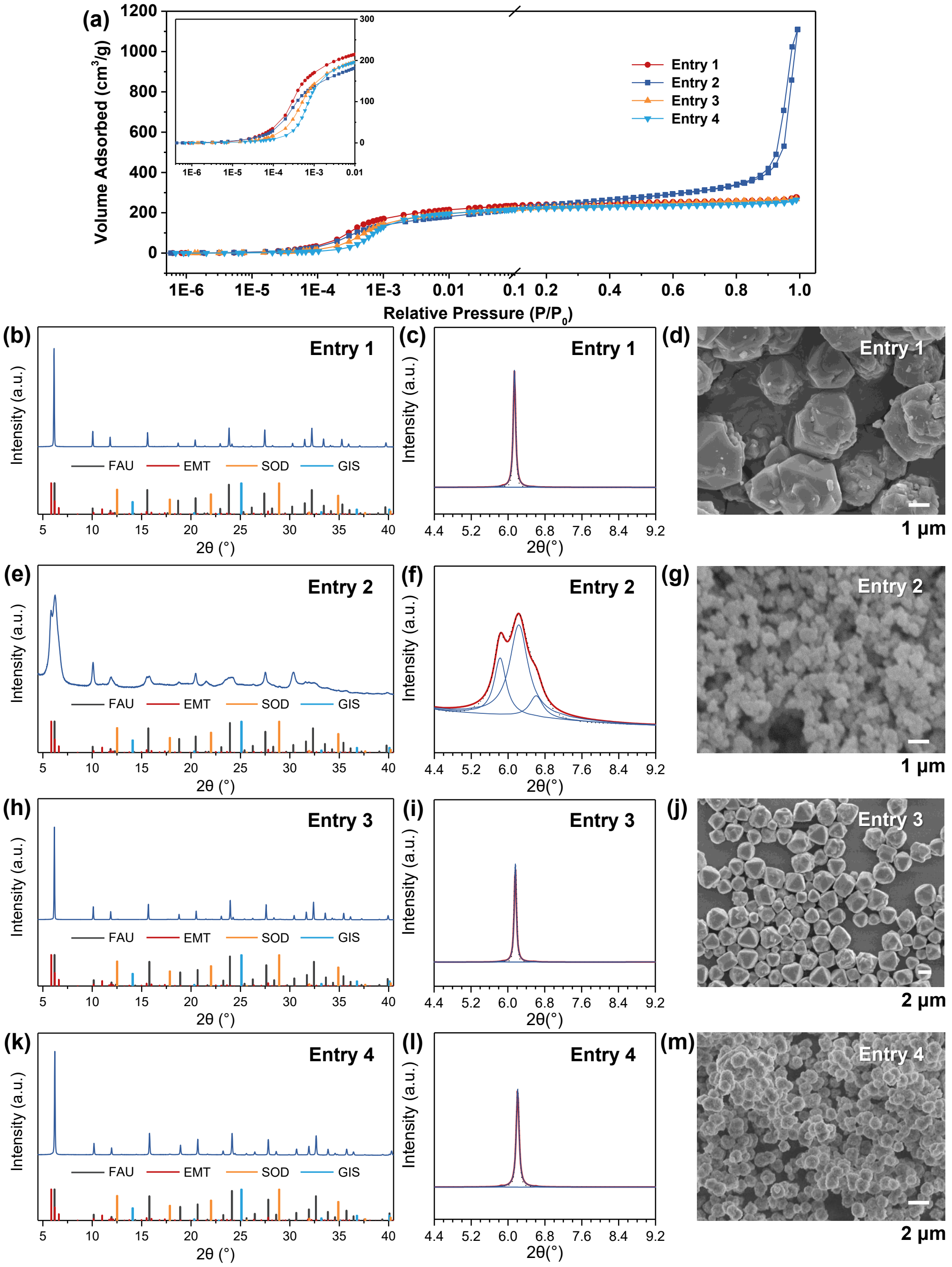

Supplementary Figure 2. Characterization results of faujasite zeolite (FAU and EMT) materials (Entries 1~4). (a) Ar-adsorption isotherms. (b)(e)(h)(k) XRD patterns (plotted for the CuKα wavelength of 1.54059 Å) for Na-FAU zeolites converted from synchrotron XRD patterns. (c)(f)(i)(l) Zoomed-in XRD patterns for the first main diffraction peak used to determine the FAU/(FAU+EMT) ratio. FAU(111) peak is centered at ~ 6.2°, and EMT(100) peak is centered at ~ 5.8°. (d)(g)(j)(m) SEM images of Na-FAU zeolites.

Supplementary Table 4. Synthesis recipes and corresponding physical properties of faujasite zeolite (FAU and EMT) materials (Entries 5~8).

Entry	SiO_2	Al_2O_3	Na_2O	$H_2O_{initial}$	H_2O_{final}	Si source	Al source	Type of oven	Cryst. Temp. (°C)	Cryst. Time (day)	Si/Al ratio via ICP	Particle Size (nm)	Crystal Size (nm)	FAU/(FAU + EMT)	uptake value at p/p_0= 0.01 (cm^3/g)
5	12.00	1.00	3.70	160.00	160.00	AS40	NaAl	static oven	100	7.00	2.793	1659	85.1	0.994	208
6	12.00	1.00	3.80	160.00	160.00	AS40	NaAl	static oven	100	5.00	2.772	1643	86.5	0.991	209
7	12.00	1.00	3.90	160.00	160.00	AS40	NaAl	static oven	100	5.00	2.693	1468	89.9	0.990	214
8	12.00	1.00	4.00	160.00	160.00	AS40	NaAl	static oven	100	3.00	2.651	1516	89.9	0.989	214

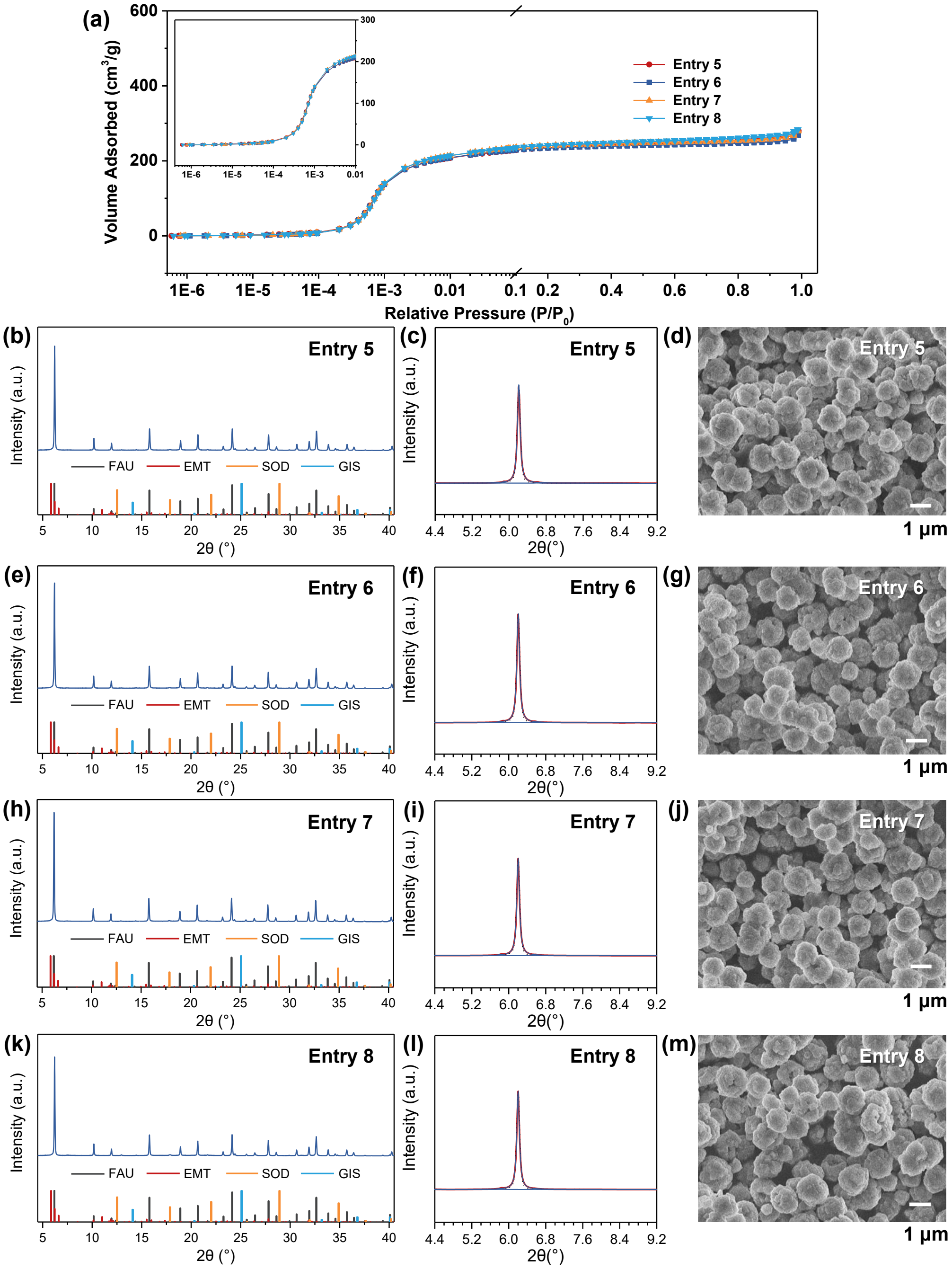

Supplementary Figure 3. Characterization results of faujasite zeolite (FAU and EMT) materials (Entries 5~8). (a) Ar-adsorption isotherms. (b)(e)(h)(k) XRD patterns (plotted for the CuKα wavelength of 1.54059 Å) for Na-FAU zeolites converted from synchrotron XRD patterns. (c)(f)(i)(l) Zoomed-in XRD patterns for the first main diffraction peak used to determine the FAU/(FAU+EMT) ratio. FAU(111) peak is centered at ~ 6.2°, and EMT(100) peak is centered at ~ 5.8°. (d)(g)(j)(m) SEM images of Na-FAU zeolites.

Supplementary Table 5. Synthesis recipes and corresponding physical properties of faujasite zeolite (FAU and EMT) materials (Entries 9~12).

Entry	SiO_2	Al_2O_3	Na_2O	$H_2O_{initial}$	H_2O_{final}	Si source	Al source	Type of oven	Cryst. Temp. (°C)	Cryst. Time (day)	Si/Al ratio via ICP	Particle Size (nm)	Crystal Size (nm)	FAU/ (FAU + EMT)	uptake value at p/p_0= 0.01 (cm^3/g)
9	12.00	1.00	4.00	160.00	160.00	AS40	NaAl	static oven	100	3.00	2.669	1570	89.9	0.995	219
10	12.00	1.00	4.00	160.00	160.00	AS40	NaAl	static oven	100	3.00	2.688	1580	85.1	0.993	224
11	12.00	1.00	4.00	160.00	160.00	AS40	Al powder	static oven	100	3.00	2.709	1900	85.4	0.994	213
12	12.00	1.00	4.00	160.00	160.00	AS40	NaAl	static oven	100	3.00	2.679	1560	88.3	0.993	190

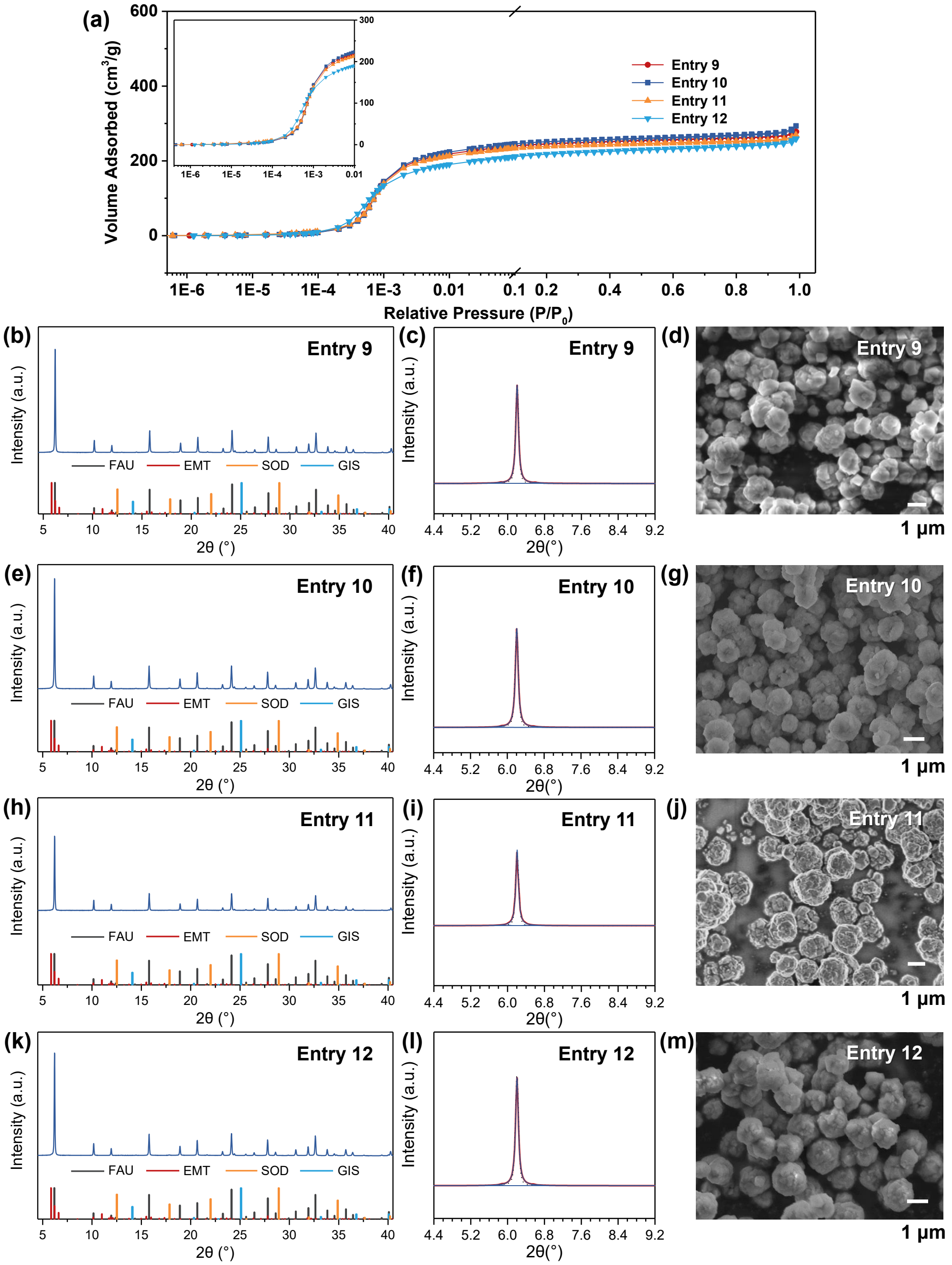

Supplementary Figure 4. Characterization results of faujasite zeolite (FAU and EMT) materials (Entries 9~12). (a) Ar-adsorption isotherms. (b)(e)(h)(k) XRD patterns (plotted for the CuKα wavelength of 1.54059 Å) for Na-FAU zeolites converted from synchrotron XRD patterns. (c)(f)(i)(l) Zoomed-in XRD patterns for the first main diffraction peak used to determine the FAU/(FAU+EMT) ratio. FAU(111) peak is centered at ~ 6.2°, and EMT(100) peak is centered at ~ 5.8°. (d)(g)(j)(m) SEM images of Na-FAU zeolites.

Supplementary Table 6. Synthesis recipes and corresponding physical properties of faujasite zeolite (FAU and EMT) materials (Entries 13~16).

Entry	SiO_2	Al_2O_3	Na_2O	$H_2O_{initial}$	H_2O_{final}	Si source	Al source	Type of oven	Cryst. Temp. (°C)	Cryst. Time (day)	Si/Al ratio via ICP	Particle Size (nm)	Crystal Size (nm)	FAU/ (FAU + EMT)	uptake value at p/p_0= 0.01 (cm^3/g)
13	12.00	1.00	4.00	160.00	160.00	AS40	NaAl	static oven	100	3.00	2.669	1480	86.5	0.994	212
14	12.00	1.00	4.00	160.00	160.00	AS40	NaAl	static oven	100	3.00	2.694	1254	82.1	0.994	198
15	12.00	1.00	4.00	160.00	160.00	AS40	Al powder	static oven	100	3.00	2.720	1477	85.1	0.994	208
16	12.00	1.00	4.00	160.00	160.00	HS30	Al powder	static oven	100	3.00	2.677	1798	86.5	0.994	216

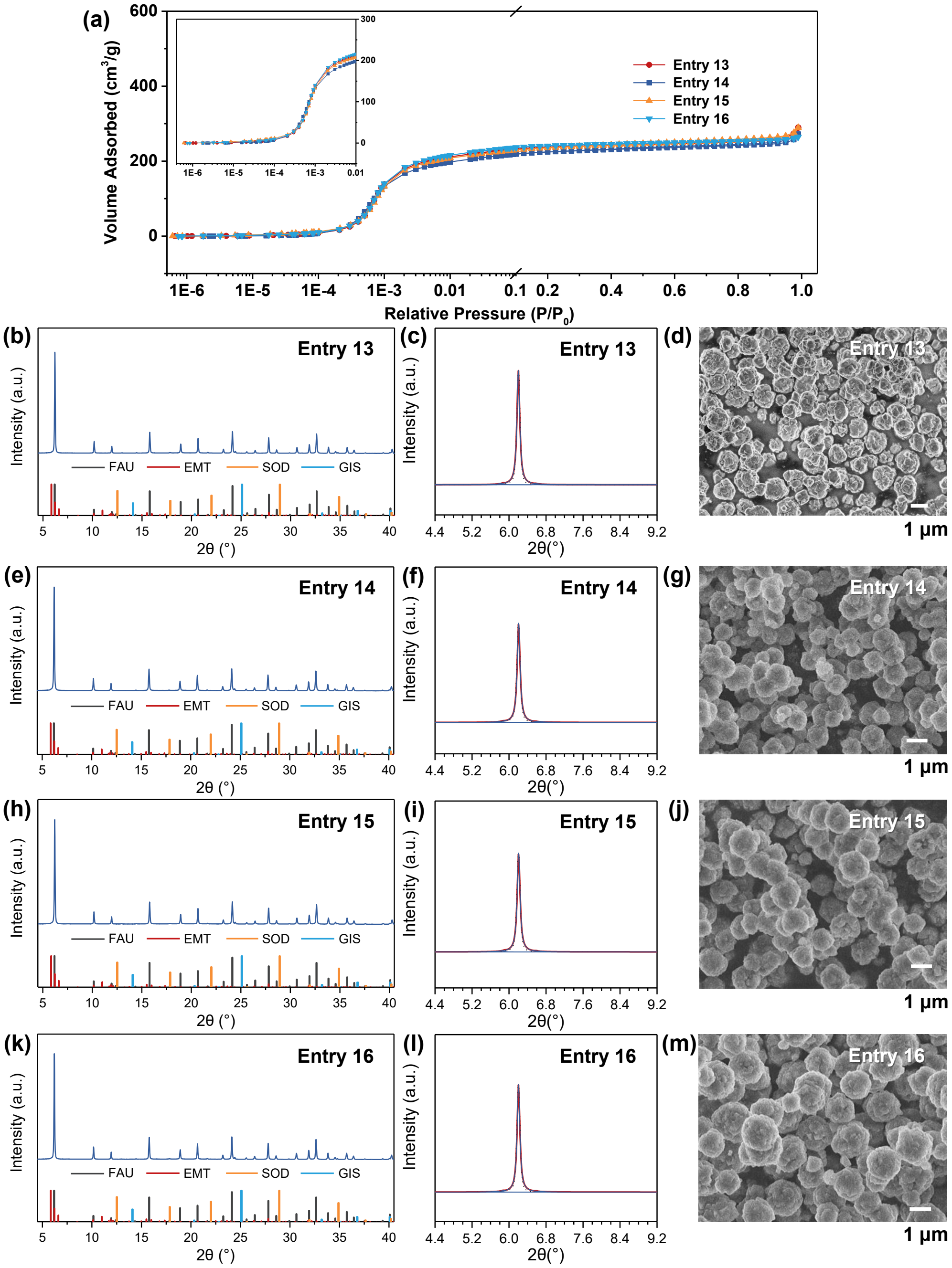

Supplementary Figure 5. Characterization results of faujasite zeolite (FAU and EMT) materials (Entries 13~16). (a) Ar-adsorption isotherms. (b)(e)(h)(k) XRD patterns (plotted for the CuKα wavelength of 1.54059 Å) for Na-FAU zeolites converted from synchrotron XRD patterns. (c)(f)(i)(l) Zoomed-in XRD patterns for the first main diffraction peak used to determine the FAU/(FAU+EMT) ratio. FAU(111) peak is centered at ~ 6.2°, and EMT(100) peak is centered at ~ 5.8°. (d)(g)(j)(m) SEM images of Na-FAU zeolites.

Supplementary Table 7. Synthesis recipes and corresponding physical properties of faujasite zeolite (FAU and EMT) materials (Entries 17~20).

Entry	SiO_2	Al_2O_3	Na_2O	$H_2O_{initial}$	H_2O_{final}	Si source	Al source	Type of oven	Cryst. Temp. (°C)	Cryst. Time (day)	Si/Al ratio via ICP	Particle Size (nm)	Crystal Size (nm)	FAU/ (FAU + EMT)	uptake value at p/p_0= 0.01 (cm^3/g)
17	12.00	1.00	4.00	160.00	160.00	AS40	NaAl	static oven	100	5.00	2.697	1579	84.8	0.999	209
18	12.00	1.00	4.00	160.00	160.00	AS40	NaAl	static oven	100	5.00	2.683	1400	85.0	0.994	234
19	12.00	1.00	4.00	160.00	160.00	AS40	Al powder	static oven	100	5.00	2.712	2160	Not collected	Not collected	226
20	12.00	1.00	4.00	160.00	160.00	AS40	NaAl	static oven	100	5.00	2.695	1293	85.1	0.992	197

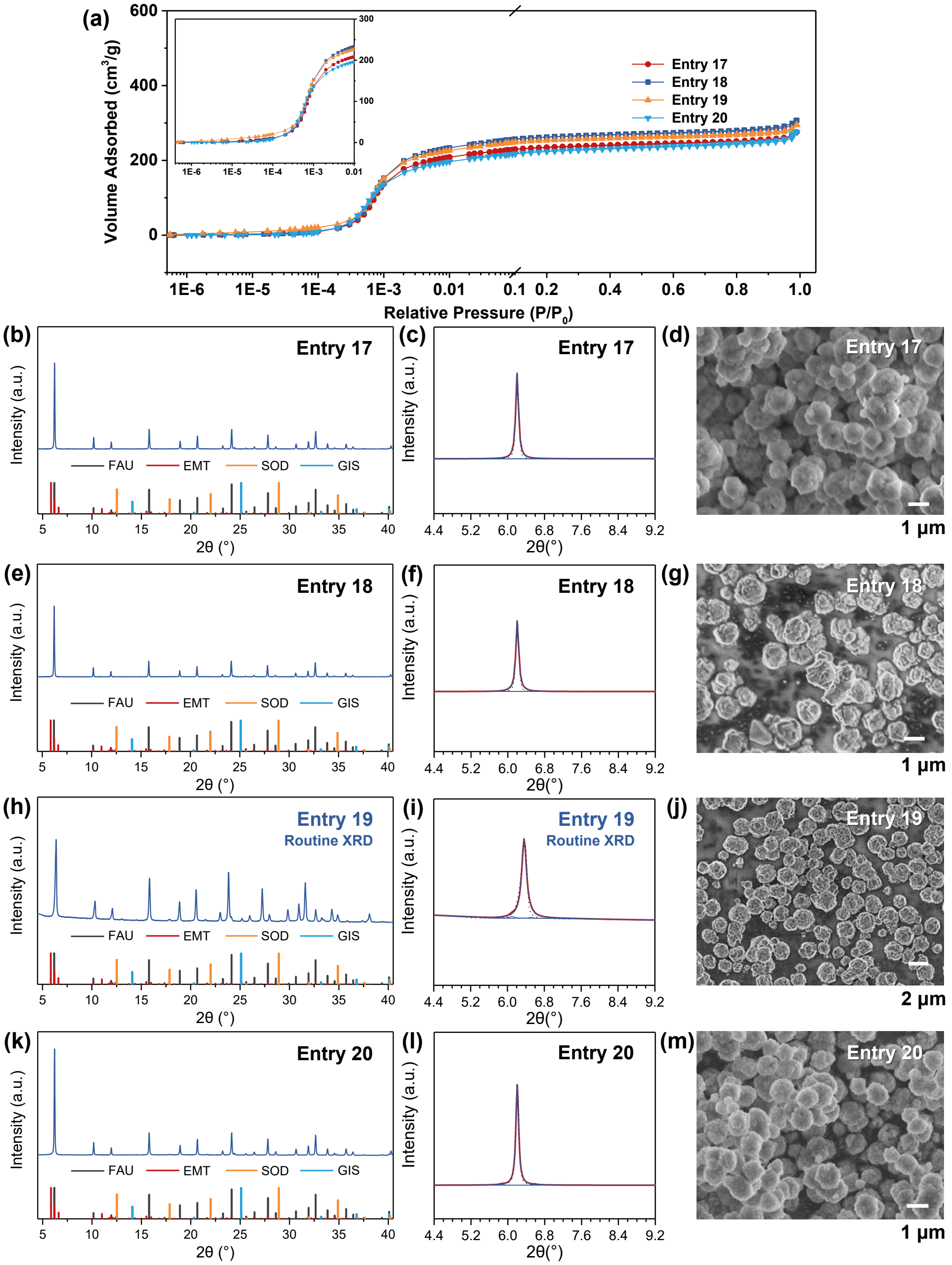

Supplementary Figure 6. Characterization results of faujasite zeolite (FAU and EMT) materials (Entries 17~20). (a) Ar-adsorption isotherms. (b)(e)(h)(k) XRD patterns (plotted for the CuKα wavelength of 1.54059 Å) for Na-FAU zeolites converted from synchrotron XRD patterns. (c)(f)(i)(l) Zoomed-in XRD patterns for the first main diffraction peak used to determine the FAU/(FAU+EMT) ratio. FAU(111) peak is centered at ~ 6.2°, and EMT(100) peak is centered at ~ 5.8°. (d)(g)(j)(m) SEM images of Na-FAU zeolites.

Supplementary Table 8. Synthesis recipes and corresponding physical properties of faujasite zeolite (FAU and EMT) materials (Entries 21~24).

Entry	SiO_2	Al_2O_3	Na_2O	$H_2O_{initial}$	H_2O_{final}	Si source	Al source	Type of oven	Cryst. Temp. (°C)	Cryst. Time (day)	Si/Al ratio via ICP	Particle Size (nm)	Crystal Size (nm)	FAU/ (FAU + EMT)	uptake value at p/p_0= 0.01 (cm^3/g)
21	12.00	1.00	4.00	160.00	160.00	HS30	Al powder	rotation oven	100	5.00	2.770	3300	47.0	0.961	201
22	12.00	1.00	4.00	160.00	160.00	AS40	NaAl	static oven	100	6.00	2.655	1470	Not collected	Not collected	212
23	12.00	1.00	4.00	160.00	160.00	AS40	Al powder	static oven	100	7.00	2.729	1197	80.2	0.998	209
24	12.00	1.00	4.00	160.00	160.00	AS40	NaAl	static oven	100	7.00	2.762	1200	82.4	0.998	196

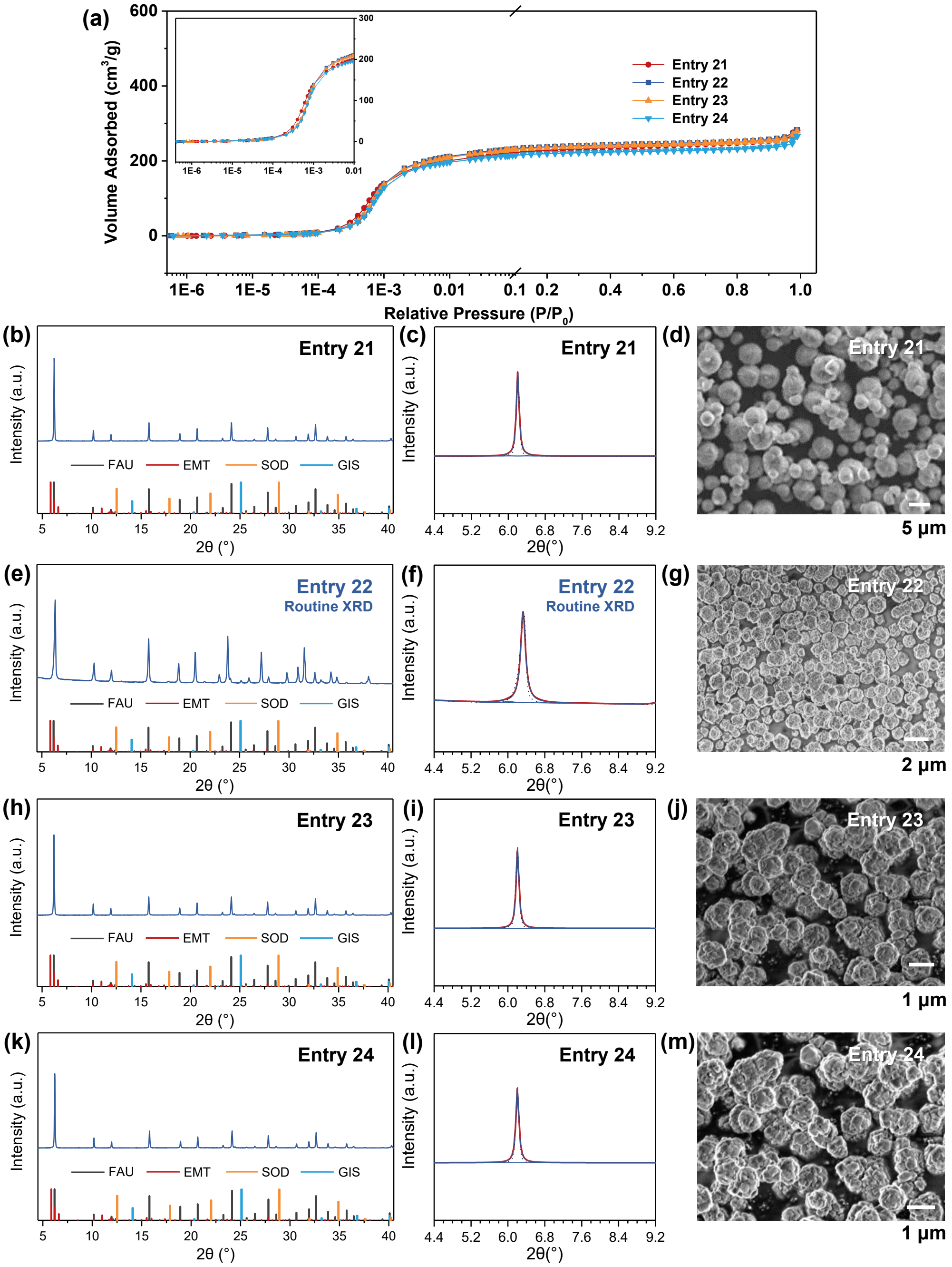

Supplementary Figure 7. Characterization results of faujasite zeolite (FAU and EMT) materials (Entries 21~24). (a) Ar-adsorption isotherms. (b)(e)(h)(k) XRD patterns (plotted for the CuKα wavelength of 1.54059 Å) for Na-FAU zeolites converted from synchrotron XRD patterns. (c)(f)(i)(l) Zoomed-in XRD patterns for the first main diffraction peak used to determine the FAU/(FAU+EMT) ratio. FAU(111) peak is centered at ~ 6.2°, and EMT(100) peak is centered at ~ 5.8°. (d)(g)(j)(m) SEM images of Na-FAU zeolites.

Supplementary Table 9. Synthesis recipes and corresponding physical properties of faujasite zeolite (FAU and EMT) materials (Entries 25~28).

Entry	SiO_2	Al_2O_3	Na_2O	$H_2O_{initial}$	H_2O_{final}	Si source	Al source	Type of oven	Cryst. Temp. (°C)	Cryst. Time (day)	Si/Al ratio via ICP	Particle Size (nm)	Crystal Size (nm)	FAU/ (FAU + EMT)	uptake value at p/p_0= 0.01 (cm^3/g)
25	12.00	1.00	4.00	160.00	160.00	AS40	Al powder	static oven	100	9.00	2.764	1368	80.6	0.993	201
26	12.50	1.00	4.00	160.00	160.00	AS40	Al powder	static oven	100	3.00	2.758	2530	83.4	0.991	168
27	12.50	1.00	4.00	160.00	160.00	AS40	Al powder	static oven	100	8.00	2.635	2170	Not collected	Not collected	212
28	12.50	1.00	4.20	166.00	166.00	AS40	NaAl	static oven	100	3.00	2.514	1657	86.5	0.989	205

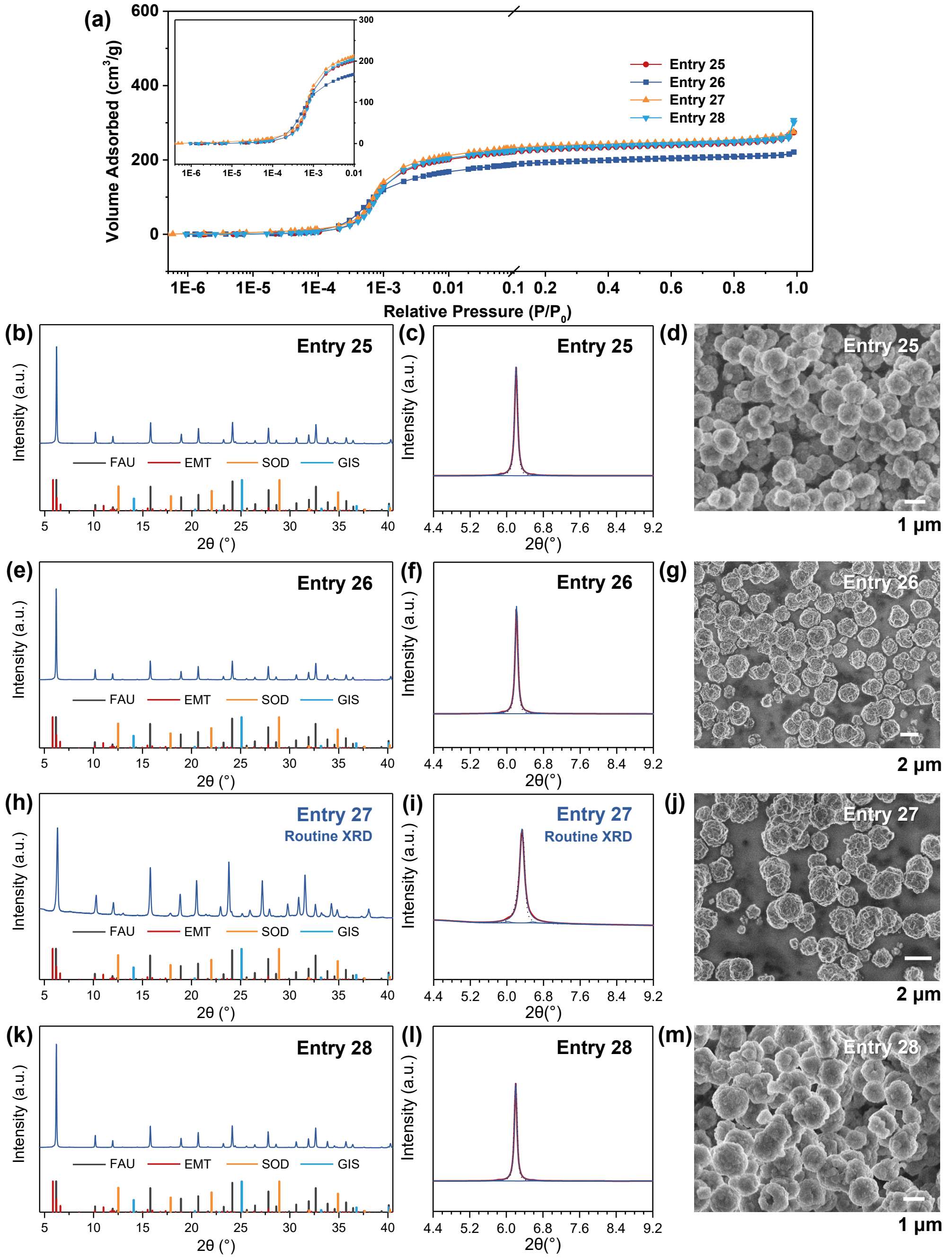

Supplementary Figure 8. Characterization results of faujasite zeolite (FAU and EMT) materials (Entries 25~28). (a) Ar-adsorption isotherms. (b)(e)(h)(k) XRD patterns (plotted for the CuKα wavelength of 1.54059 Å) for Na-FAU zeolites converted from synchrotron XRD patterns. (c)(f)(i)(l) Zoomed-in XRD patterns for the first main diffraction peak used to determine the FAU/(FAU+EMT) ratio. FAU(111) peak is centered at ~ 6.2°, and EMT(100) peak is centered at ~ 5.8°. (d)(g)(j)(m) SEM images of Na-FAU zeolites.

Supplementary Table 10. Synthesis recipes and corresponding physical properties of faujasite zeolite (FAU and EMT) materials (Entries 29~32).

Entry	SiO_2	Al_2O_3	Na_2O	$H_2O_{initial}$	H_2O_{final}	Si source	Al source	Type of oven	Cryst. Temp. (°C)	Cryst. Time (day)	Si/Al ratio via ICP	Particle Size (nm)	Crystal Size (nm)	FAU/ (FAU + EMT)	uptake value at p/p_0= 0.01 (cm^3/g)
29	13.33	1.00	4.44	177.78	177.78	AS40	NaAl	static oven	100	5.00	2.593	1803	16.2	0.992	220
30	12.00	1.00	4.00	140.00	140.00	AS40	NaAl	static oven	100	3.00	2.580	1340	82.6	0.996	222
31	12.00	1.00	4.00	140.00	140.00	AS40	Al powder	static oven	100	3.00	2.577	1604	82.1	0.993	178
32	12.00	1.00	4.00	140.00	140.00	AS40	Al powder	static oven	100	5.00	2.575	1741	85.6	0.991	216

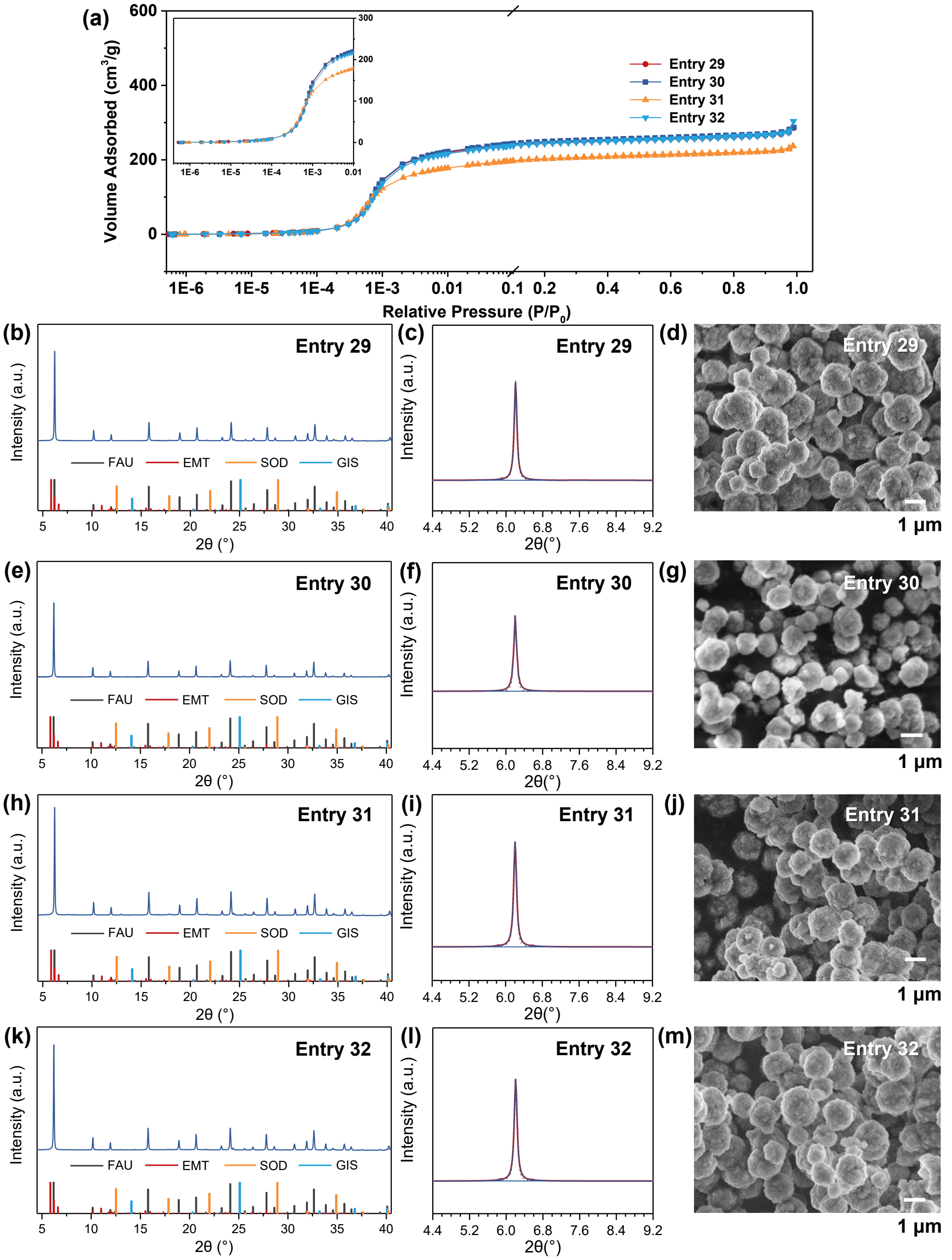

Supplementary Figure 9. Characterization results of faujasite zeolite (FAU and EMT) materials (Entries 29~32). (a) Ar-adsorption isotherms. (b)(e)(h)(k) XRD patterns (plotted for the CuKα wavelength of 1.54059 Å) for Na-FAU zeolites converted from synchrotron XRD patterns. (c)(f)(i)(l) Zoomed-in XRD patterns for the first main diffraction peak used to determine the FAU/(FAU+EMT) ratio. FAU(111) peak is centered at ~ 6.2°, and EMT(100) peak is centered at ~ 5.8°. (d)(g)(j)(m) SEM images of Na-FAU zeolites.

Supplementary Table 11. Synthesis recipes and corresponding physical properties of faujasite zeolite (FAU and EMT) materials (Entries 33~36).

Entry	SiO_2	Al_2O_3	Na_2O	$H_2O_{initial}$	H_2O_{final}	Si source	Al source	Type of oven	Cryst. Temp. (°C)	Cryst. Time (day)	Si/Al ratio via ICP	Particle Size (nm)	Crystal Size (nm)	FAU/ (FAU + EMT)	uptake value at p/p_0= 0.01 (cm^3/g)
33	12.00	1.00	4.00	140.00	140.00	AS40	Al powder	static oven	100	5.00	2.561	1497	79.3	0.992	208
34	12.00	1.00	4.00	140.00	140.00	AS40	Al powder	static oven	100	7.00	2.558	1340	80.8	0.992	208
35	12.00	1.00	4.00	140.00	140.00	AS40	Al powder	static oven	100	7.00	2.683	1764	85.1	0.992	213
36	10.00	1.00	10.00	214.00	214.00	NaSi	NaAl	static oven	50	6.00	1.288	75	13.5	0.499	142

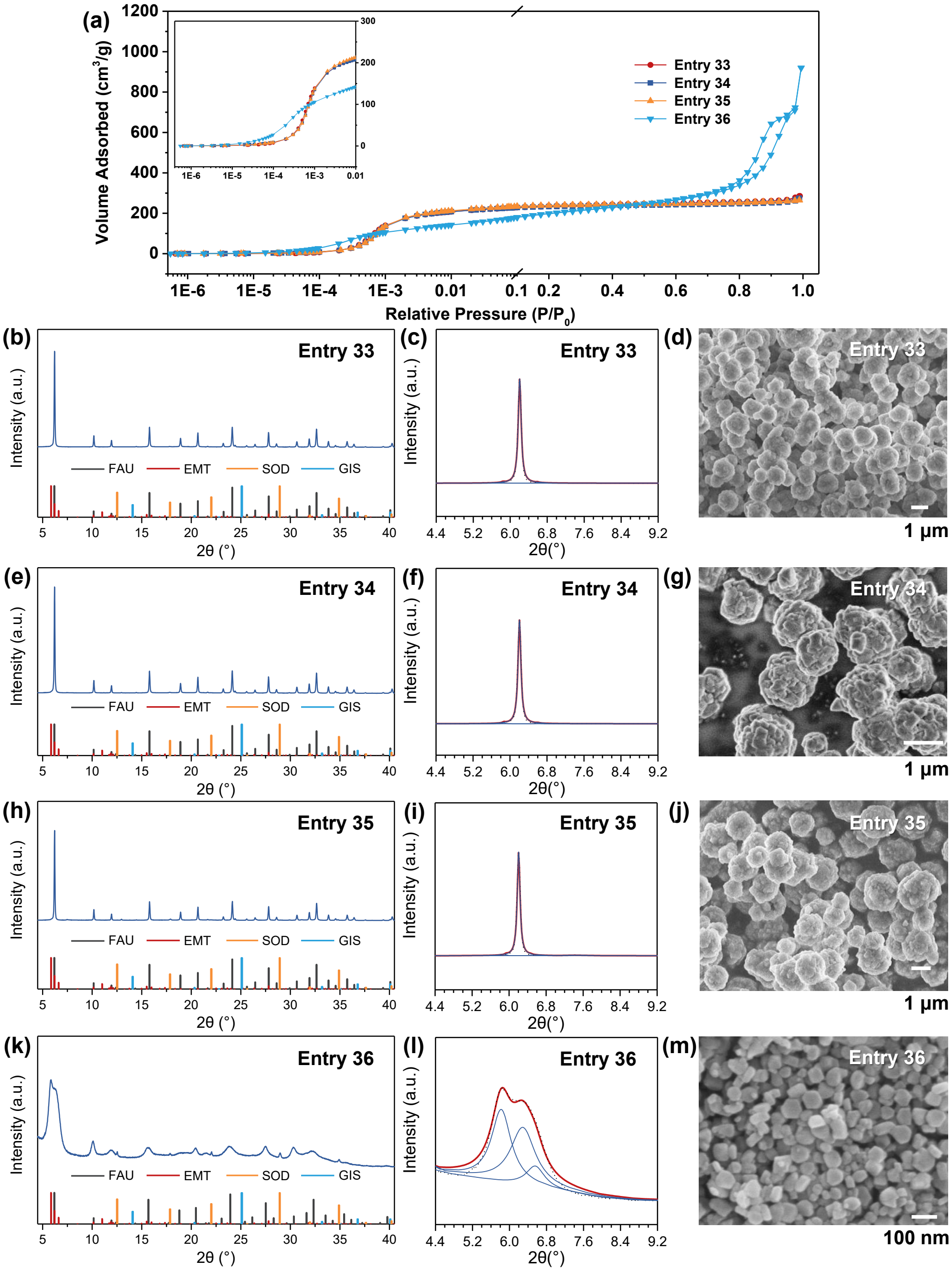

Supplementary Figure 10. Characterization results of faujasite zeolite (FAU and EMT) materials (Entries 33~36). (a) Ar-adsorption isotherms. (b)(e)(h)(k) XRD patterns (plotted for the CuKα wavelength of 1.54059 Å) for Na-FAU zeolites converted from synchrotron XRD patterns. (c)(f)(i)(l) Zoomed-in XRD patterns for the first main diffraction peak used to determine the FAU/(FAU+EMT) ratio. FAU(111) peak is centered at ~ 6.2°, and EMT(100) peak is centered at ~ 5.8°. (d)(g)(j)(m) SEM images of Na-FAU zeolites.

Supplementary Table 12. Synthesis recipes and corresponding physical properties of faujasite zeolite (FAU and EMT) materials (Entries 37~40).

Entry	SiO_2	Al_2O_3	Na_2O	$H_2O_{initial}$	H_2O_{final}	Si source	Al source	Type of oven	Cryst. Temp. (°C)	Cryst. Time (day)	Si/Al ratio via ICP	Particle Size (nm)	Crystal Size (nm)	FAU/ (FAU + EMT)	uptake value at p/p_0= 0.01 (cm^3/g)
37	12.00	1.00	5.00	160.00	160.00	AS40	Al powder	static oven	100	3.00	2.251	876	83.4	0.993	208
38	12.00	1.00	5.15	160.00	160.00	AS40	NaAl	static oven	100	3.00	2.158	870	83.9	0.997	214
39	12.00	1.00	5.15	160.00	160.00	AS40	NaAl	static oven	100	5.00	2.150	880	82.9	0.998	211
40	12.00	1.00	4.00	120.00	120.00	AS40	Al powder	static oven	100	3.00	2.669	1870	80.8	0.993	208

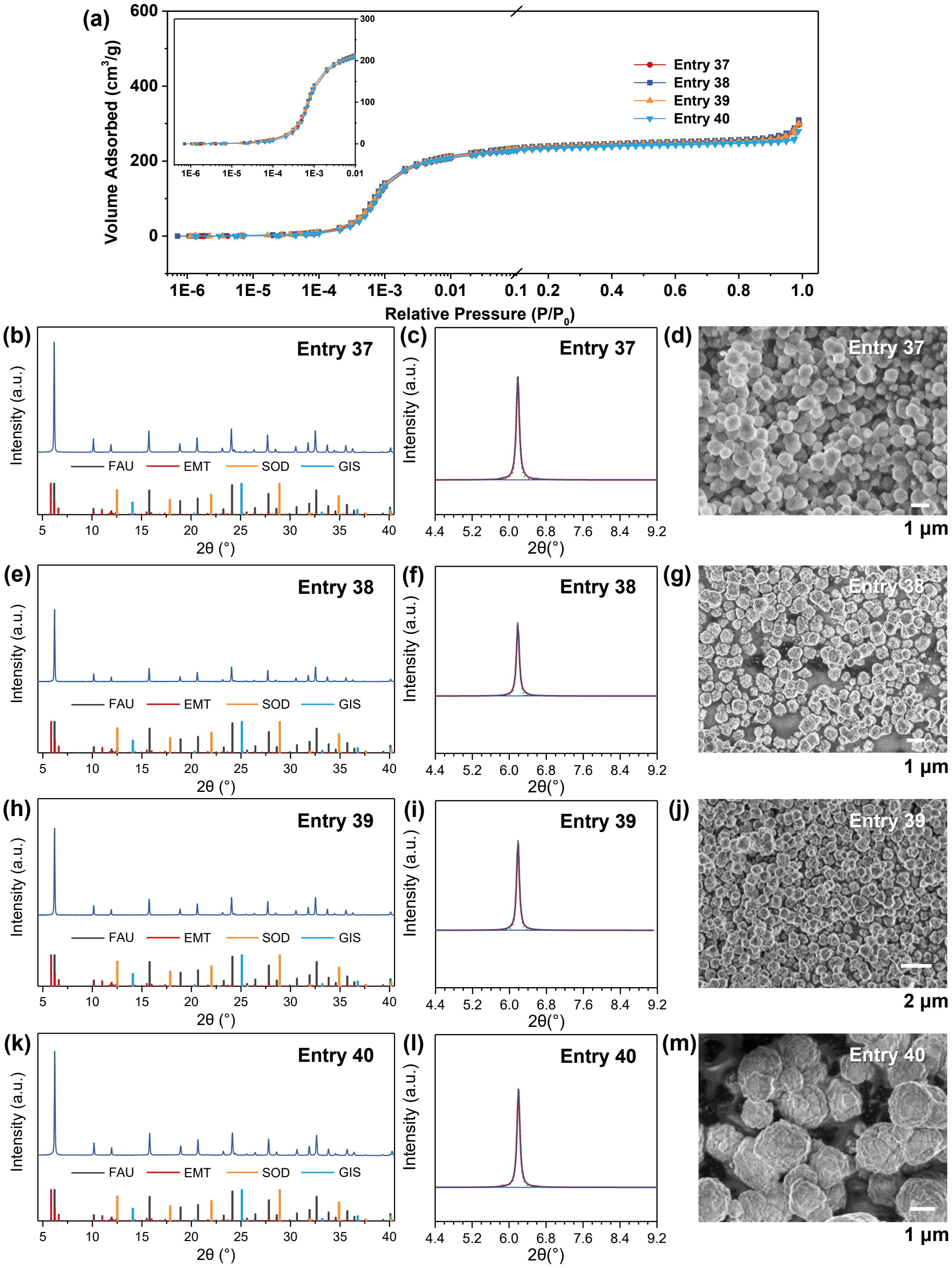

Supplementary Figure 11. Characterization results of faujasite zeolite (FAU and EMT) materials (Entries 37~40). (a) Ar-adsorption isotherms. (b)(e)(h)(k) XRD patterns (plotted for the CuKα wavelength of 1.54059 Å) for Na-FAU zeolites converted from synchrotron XRD patterns. (c)(f)(i)(l) Zoomed-in XRD patterns for the first main diffraction peak used to determine the FAU/(FAU+EMT) ratio. FAU(111) peak is centered at ~ 6.2°, and EMT(100) peak is centered at ~ 5.8°. (d)(g)(j)(m) SEM images of Na-FAU zeolites.

Supplementary Table 13. Synthesis recipes and corresponding physical properties of faujasite zeolite (FAU and EMT) materials (Entries 41~44).

Entry	SiO_2	Al_2O_3	Na_2O	$H_2O_{initial}$	H_2O_{final}	Si source	Al source	Type of oven	Cryst. Temp. (°C)	Cryst. Time (day)	Si/Al ratio via ICP	Particle Size (nm)	Crystal Size (nm)	FAU/ (FAU + EMT)	uptake value at p/p_0= 0.01 (cm^3/g)
41	12.00	1.00	4.00	120.00	120.00	AS40	NaAl	static oven	100	3.00	2.614	1700	78.1	0.992	108
42	12.00	1.00	12.00	214.00	214.00	NaSi	NaAl	static oven	50	6.00	1.266	161	13.5	0.469	110
43	12.00	1.00	6.00	140.00	140.00	HS30	Al powder	rotation oven	90	4.00	1.886	904	82.5	0.989	171
44	14.00	1.00	9.50	214.00	214.00	NaSi	NaAl	static oven	65	2.00	1.814	1200	57.2	0.954	175

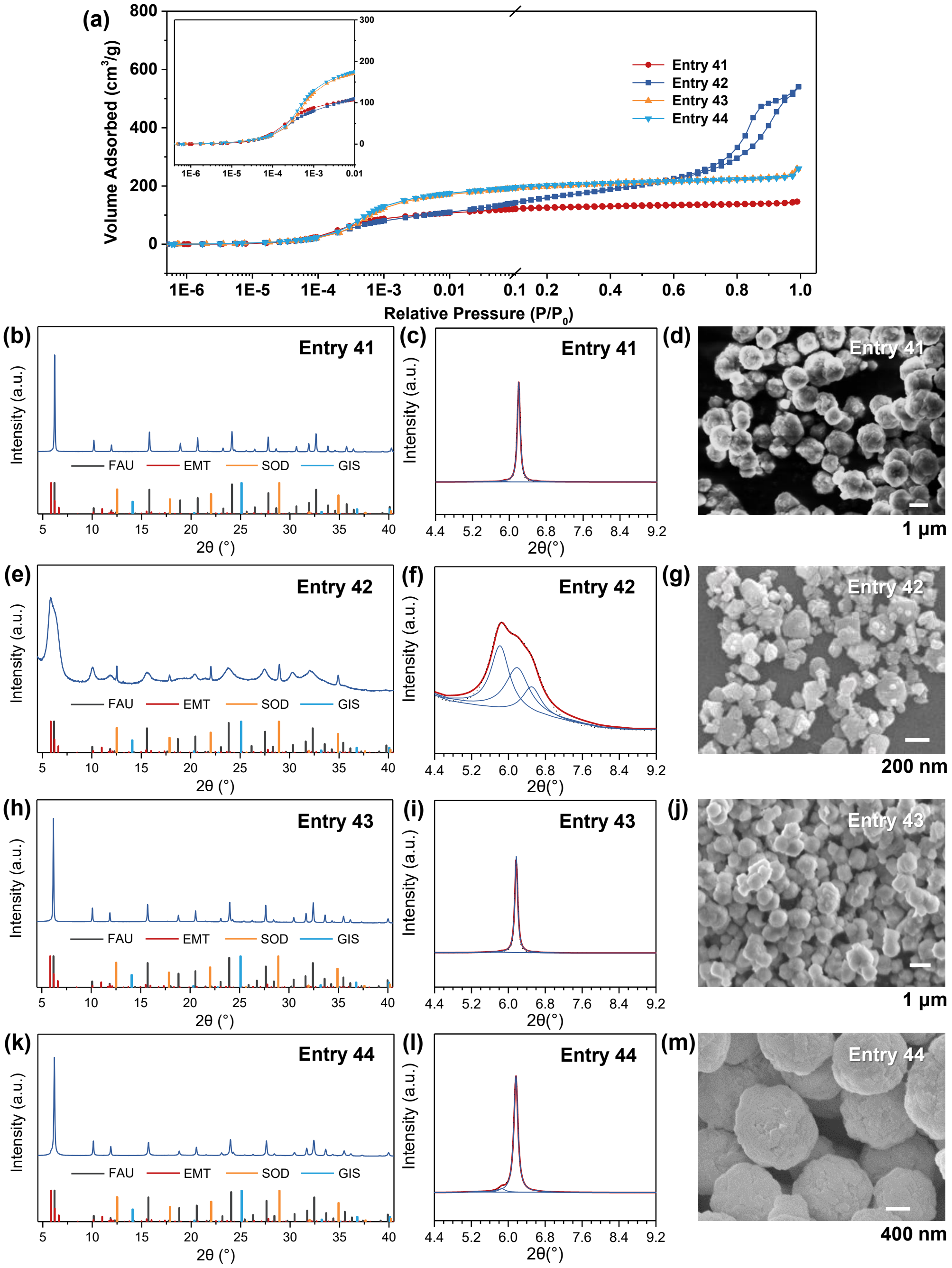

Supplementary Figure 12. Characterization results of faujasite zeolite (FAU and EMT) materials (Entries 41~44). (a) Ar-adsorption isotherms. (b)(e)(h)(k) XRD patterns (plotted for the CuKα wavelength of 1.54059 Å) for Na-FAU zeolites converted from synchrotron XRD patterns. (c)(f)(i)(l) Zoomed-in XRD patterns for the first main diffraction peak used to determine the FAU/(FAU+EMT) ratio. FAU(111) peak is centered at ~ 6.2°, and EMT(100) peak is centered at ~ 5.8°. (d)(g)(j)(m) SEM images of Na-FAU zeolites.

Supplementary Table 14. Synthesis recipes and corresponding physical properties of faujasite zeolite (FAU and EMT) materials (Entries 45~48).

Entry	SiO_2	Al_2O_3	Na_2O	$H_2O_{initial}$	H_2O_{final}	Si source	Al source	Type of oven	Cryst. Temp. (°C)	Cryst. Time (day)	Si/Al ratio via ICP	Particle Size (nm)	Crystal Size (nm)	FAU/ (FAU + EMT)	uptake value at p/p_0= 0.01 (cm^3/g)
45	14.00	1.00	10.00	214.00	214.00	NaSi	NaAl	static oven	65	2.00	1.593	560	43.8	0.892	185
46	14.00	1.00	10.00	214.00	214.00	NaSi	NaAl	static oven	50	6.00	1.709	293	25.2	0.876	177
47	14.00	1.00	10.50	214.00	214.00	NaSi	NaAl	static oven	65	2.00	1.493	239	25.2	0.801	204
48	14.00	1.00	11.00	214.00	214.00	NaSi	NaAl	static oven	65	2.00	1.479	186	25.0	0.804	191

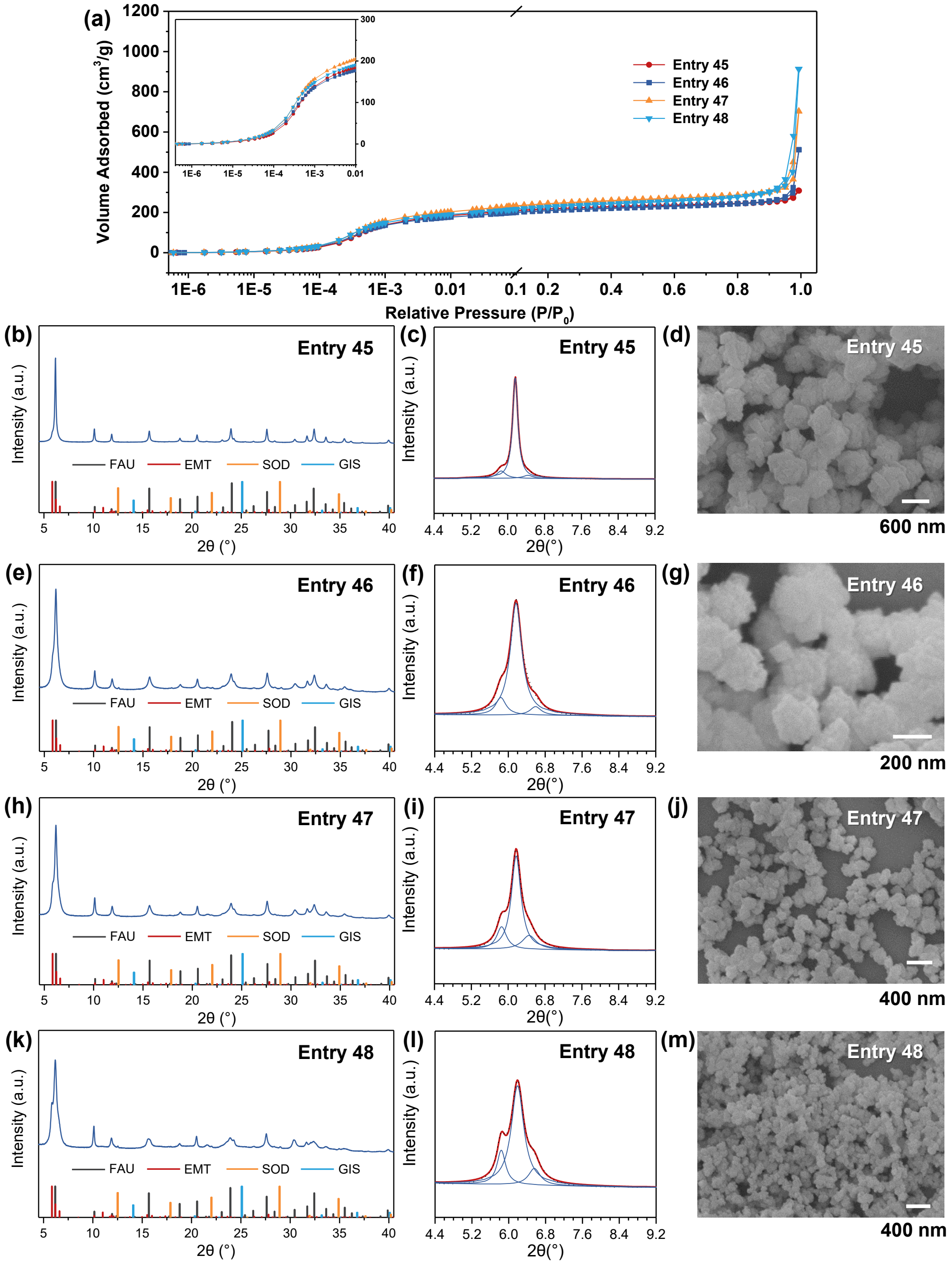

Supplementary Figure 13. Characterization results of faujasite zeolite (FAU and EMT) materials (Entries 45~48). (a) Ar-adsorption isotherms. (b)(e)(h)(k) XRD patterns (plotted for the CuKα wavelength of 1.54059 Å) for Na-FAU zeolites converted from synchrotron XRD patterns. (c)(f)(i)(l) Zoomed-in XRD patterns for the first main diffraction peak used to determine the FAU/(FAU+EMT) ratio. FAU(111) peak is centered at ~ 6.2°, and EMT(100) peak is centered at ~ 5.8°. (d)(g)(j)(m) SEM images of Na-FAU zeolites.

Supplementary Table 15. Synthesis recipes and corresponding physical properties of faujasite zeolite (FAU and EMT) materials (Entries 49~52).

Entry	SiO_2	Al_2O_3	Na_2O	$H_2O_{initial}$	H_2O_{final}	Si source	Al source	Type of oven	Cryst. Temp. (°C)	Cryst. Time (day)	Si/Al ratio via ICP	Particle Size (nm)	Crystal Size (nm)	FAU/ (FAU + EMT)	uptake value at p/p_0= 0.01 (cm^3/g)
49	14.00	1.00	11.50	214.00	214.00	NaSi	NaAl	static oven	65	2.00	1.430	193	23.7	0.783	163
50	14.00	1.00	18.00	214.00	214.00	NaSi	NaAl	static oven	50	6.00	1.088	846	40.6	0.863	170
51	14.00	1.00	12.00	214.00	214.00	NaSi	NaAl	oil bath	50	2.00	1.419	94	16.2	0.712	192
52	14.00	1.00	12.00	214.00	214.00	HS30	NaAl	oil bath	50	2.00	1.516	92	18.9	0.749	114

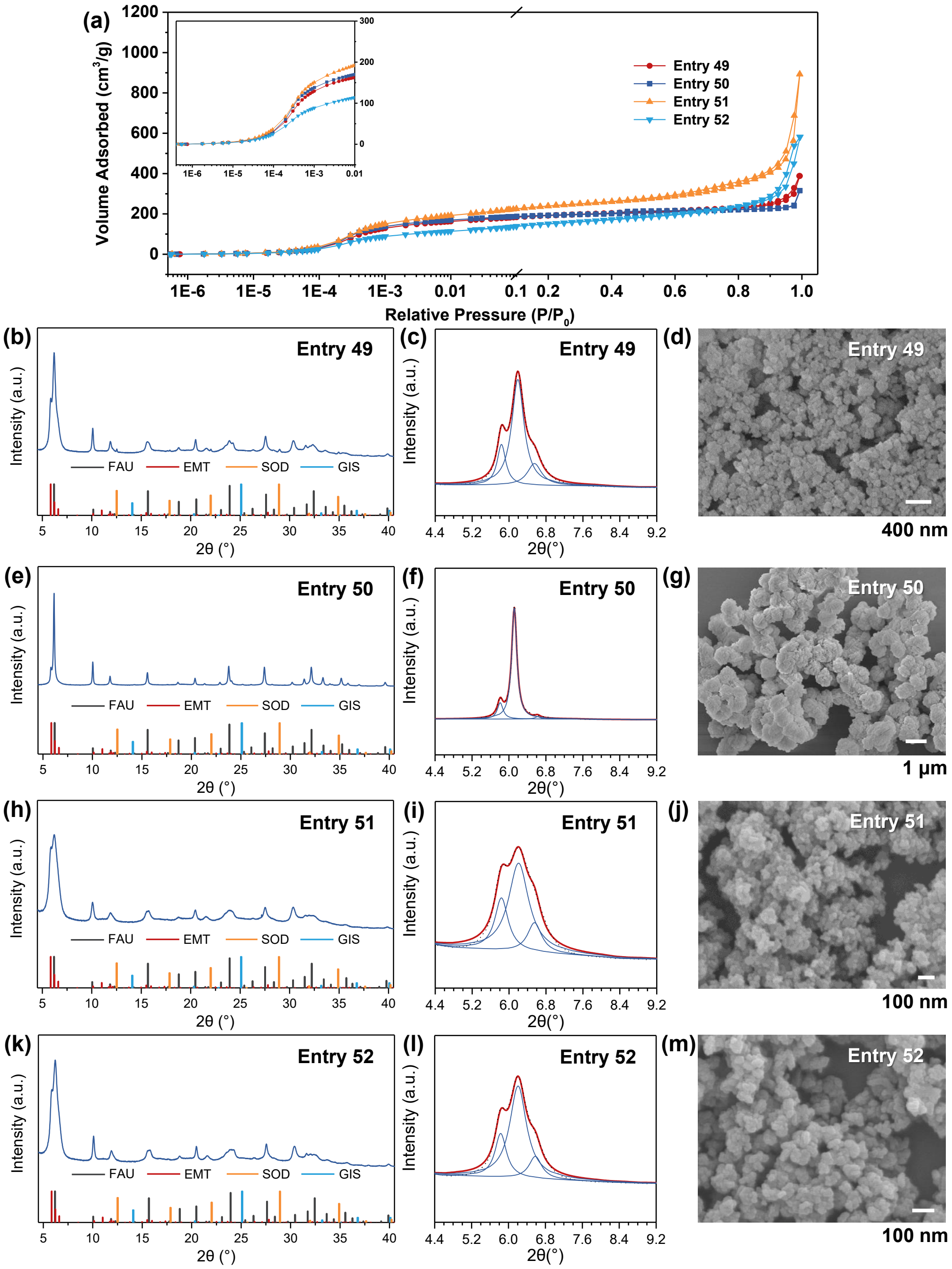

Supplementary Figure 14. Characterization results of faujasite zeolite (FAU and EMT) materials (Entries 49~52). (a) Ar-adsorption isotherms. (b)(e)(h)(k) XRD patterns (plotted for the CuKα wavelength of 1.54059 Å) for Na-FAU zeolites converted from synchrotron XRD patterns. (c)(f)(i)(l) Zoomed-in XRD patterns for the first main diffraction peak used to determine the FAU/(FAU+EMT) ratio. FAU(111) peak is centered at ~ 6.2°, and EMT(100) peak is centered at ~ 5.8°. (d)(g)(j)(m) SEM images of Na-FAU zeolites.

Supplementary Table 16. Synthesis recipes and corresponding physical properties of faujasite zeolite (FAU and EMT) materials (Entries 53~56).

Entry	SiO_2	Al_2O_3	Na_2O	$H_2O_{initial}$	H_2O_{final}	Si source	Al source	Type of oven	Cryst. Temp. (°C)	Cryst. Time (day)	Si/Al ratio via ICP	Particle Size (nm)	Crystal Size (nm)	FAU/ (FAU + EMT)	uptake value at p/p_0= 0.01 (cm^3/g)
53	14.00	1.00	12.00	214.00	214.00	HS30	Al foil1	oil bath	50	2.00	1.398	141	21.0	0.787	113
54	14.00	1.00	12.00	214.00	214.00	NaSi	NaAl	static oven	50	6.00	1.442	481	20.3	0.767	173
55	14.00	1.00	12.00	214.00	214.00	NaSi	NaAl	static oven	65	2.00	1.301	254	32.5	0.863	192
56	14.00	1.00	16.00	214.00	214.00	NaSi	NaAl	static oven	50	6.00	1.167	323	16.8	0.618	175

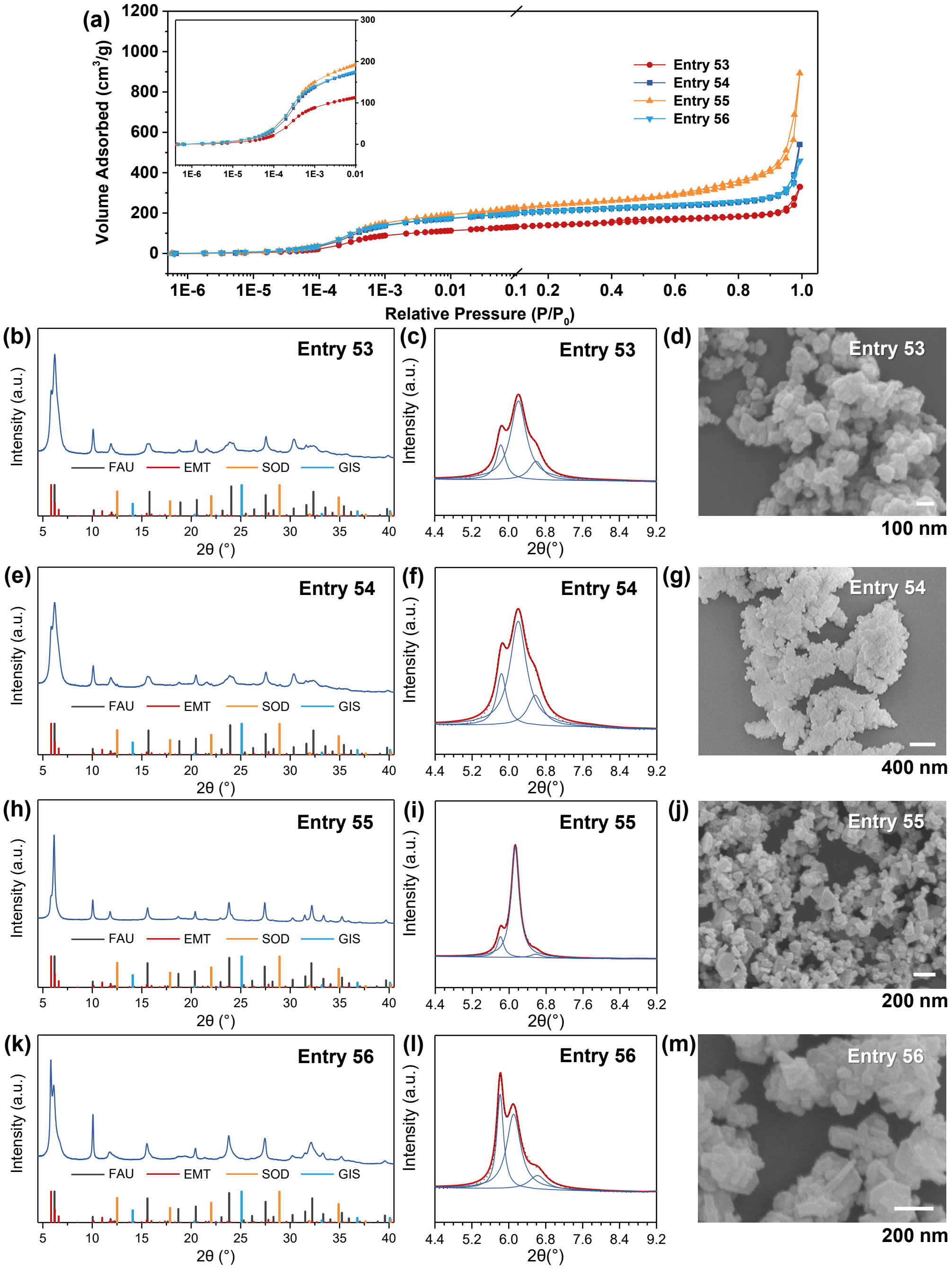

Supplementary Figure 15. Characterization results of faujasite zeolite (FAU and EMT) materials (Entries 53~56). (a) Ar-adsorption isotherms. (b)(e)(h)(k) XRD patterns (plotted for the CuKα wavelength of 1.54059 Å) for Na-FAU zeolites converted from synchrotron XRD patterns. (c)(f)(i)(l) Zoomed-in XRD patterns for the first main diffraction peak used to determine the FAU/(FAU+EMT) ratio. FAU(111) peak is centered at ~ 6.2°, and EMT(100) peak is centered at ~ 5.8°. (d)(g)(j)(m) SEM images of Na-FAU zeolites.

Supplementary Table 17. Synthesis recipes and corresponding physical properties of faujasite zeolite (FAU and EMT) materials (Entries 57~60).

Entry	SiO_2	Al_2O_3	Na_2O	$H_2O_{initial}$	H_2O_{final}	Si source	Al source	Type of oven	Cryst. Temp. (°C)	Cryst. Time (day)	Si/Al ratio via ICP	Particle Size (nm)	Crystal Size (nm)	FAU/ (FAU + EMT)	uptake value at p/p_0= 0.01 (cm^3/g)
57	14.00	1.00	14.00	214.00	214.00	NaSi	NaAl	static oven	50	6.00	1.281	253	18.6	0.679	129
58	14.00	1.00	14.00	214.00	214.00	NaSi	NaAl	static oven	65	2.00	1.281	200	13.0	0.432	125
59	14.00	1.00	14.00	214.00	214.00	NaSi	NaAl	static oven	65	2.00	1.153	193	49.2	0.887	146
60	8.00	1.00	8.00	142.67	66.67	HS30	Al powder	rotation oven	50	6.00	1.202	16	14.4	0.447	38

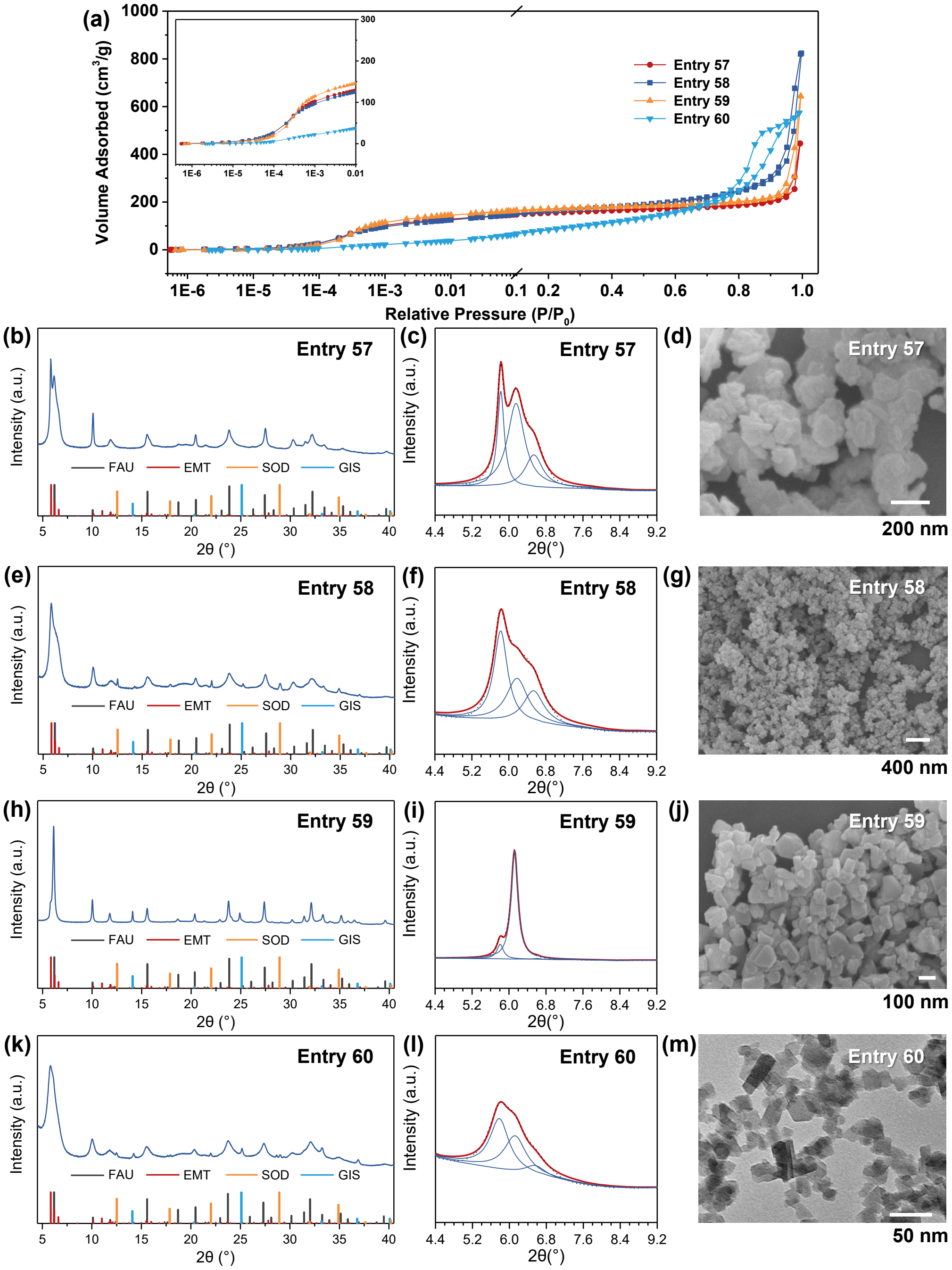

Supplementary Figure 16. Characterization results of faujasite zeolite (FAU and EMT) materials (Entries 57~60). (a) Ar-adsorption isotherms. (b)(e)(h)(k) XRD patterns (plotted for the CuKα wavelength of 1.54059 Å) for Na-FAU zeolites converted from synchrotron XRD patterns. (c)(f)(i)(l) Zoomed-in XRD patterns for the first main diffraction peak used to determine the FAU/(FAU+EMT) ratio. FAU(111) peak is centered at ~ 6.2°, and EMT(100) peak is centered at ~ 5.8°. (d)(g)(j) SEM and (m) TEM images of Na-FAU zeolites.

Supplementary Table 18. Synthesis recipes and corresponding physical properties of faujasite zeolite (FAU and EMT) materials (Entries 61~64).

Entry	SiO_2	Al_2O_3	Na_2O	$H_2O_{initial}$	H_2O_{final}	Si source	Al source	Type of oven	Cryst. Temp. (°C)	Cryst. Time (day)	Si/Al ratio via ICP	Particle Size (nm)	Crystal Size (nm)	FAU/ (FAU + EMT)	uptake value at p/p_0= 0.01 (cm^3/g)
61	10.00	1.00	10.00	214.00	100.00	NaSi	NaAl	static oven	50	6.00	1.160	36	17.0	0.589	170
62	16.00	1.00	16.00	214.00	214.00	NaSi	NaAl	static oven	50	6.00	1.244	660	49.3	0.828	138
63	10.00	1.00	10.00	178.33	83.33	HS30	Al powder	rotation oven	50	6.00	1.150	19	18.1	0.753	170
64	10.91	1.00	10.91	194.55	90.91	HS30	Al powder	rotation oven	50	6.00	1.065	22	17.3	0.850	224

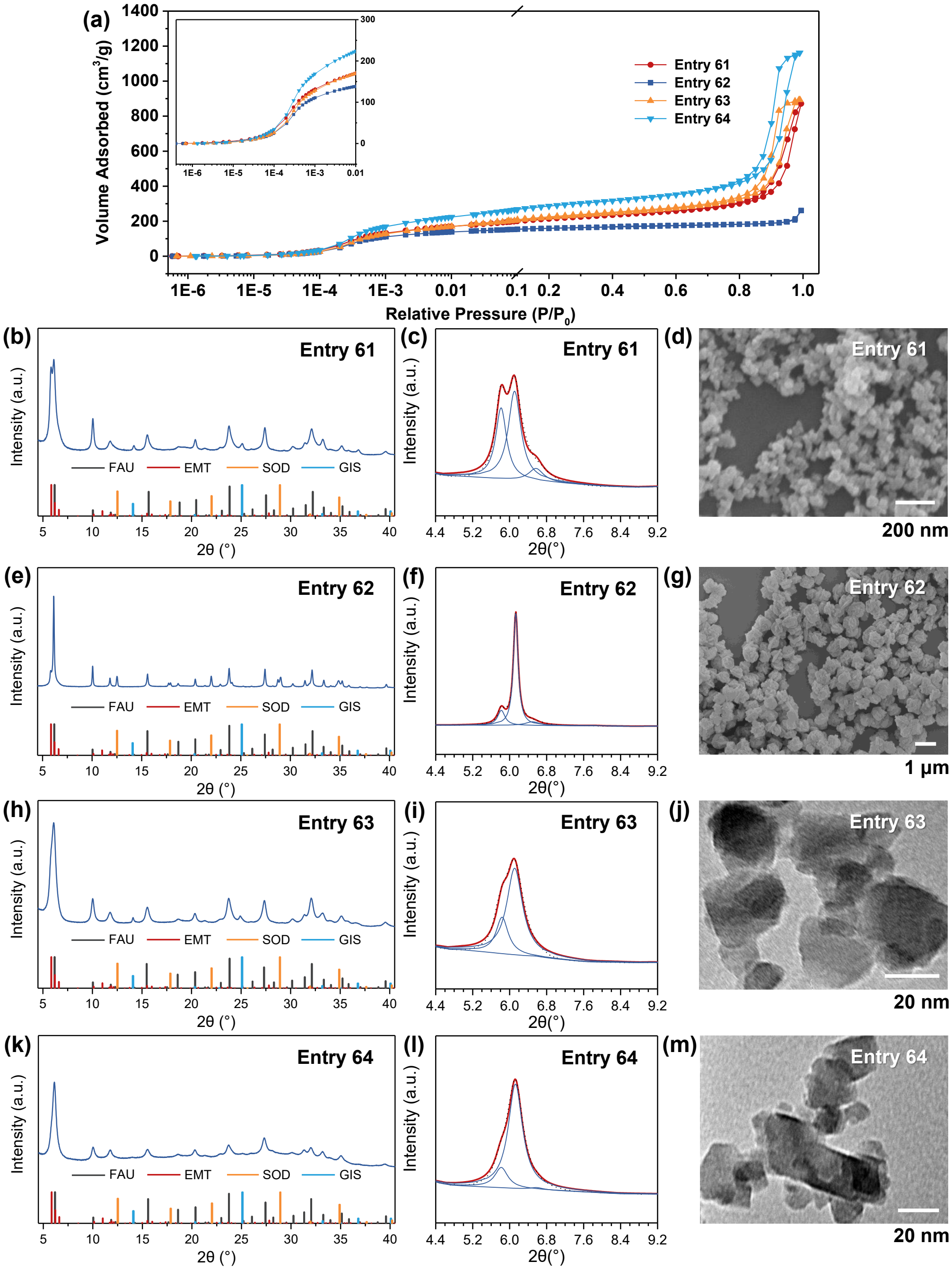

Supplementary Figure 17. Characterization results of faujasite zeolite (FAU and EMT) materials (Entries 61~64). (a) Ar-adsorption isotherms. (b)(e)(h)(k) XRD patterns (plotted for the CuKα wavelength of 1.54059 Å) for Na-FAU zeolites converted from synchrotron XRD patterns. (c)(f)(i)(l) Zoomed-in XRD patterns for the first main diffraction peak used to determine the FAU/(FAU+EMT) ratio. FAU(111) peak is centered at ~ 6.2°, and EMT(100) peak is centered at ~ 5.8°. (d)(g) SEM and (j)(m) TEM images of Na-FAU zeolites.

Supplementary Table 19. Synthesis recipes and corresponding physical properties of faujasite zeolite (FAU and EMT) materials (Entries 65~68).

Entry	SiO_2	Al_2O_3	Na_2O	$H_2O_{initial}$	H_2O_{final}	Si source	Al source	Type of oven	Cryst. Temp. (°C)	Cryst. Time (day)	Si/Al ratio via ICP	Particle Size (nm)	Crystal Size (nm)	FAU/ (FAU + EMT)	uptake value at p/p_0= 0.01 (cm^3/g)
65	12.00	1.00	8.00	214.00	100.00	HS30	Al powder	rotation oven	50	6.00	1.759	161	26.4	0.807	124
66	12.00	1.00	12.00	214.00	100.00	HS30	Al powder	rotation oven	50	5.00	1.190	29	21.5	0.865	220
67	12.00	1.00	12.00	214.00	100.00	NaSi	NaAl	static oven	50	6.00	1.232	32	21.6	0.834	162
68	12.00	1.00	9.00	214.00	100.00	HS30	Al powder	rotation oven	50	6.00	1.513	147	26.0	0.808	130

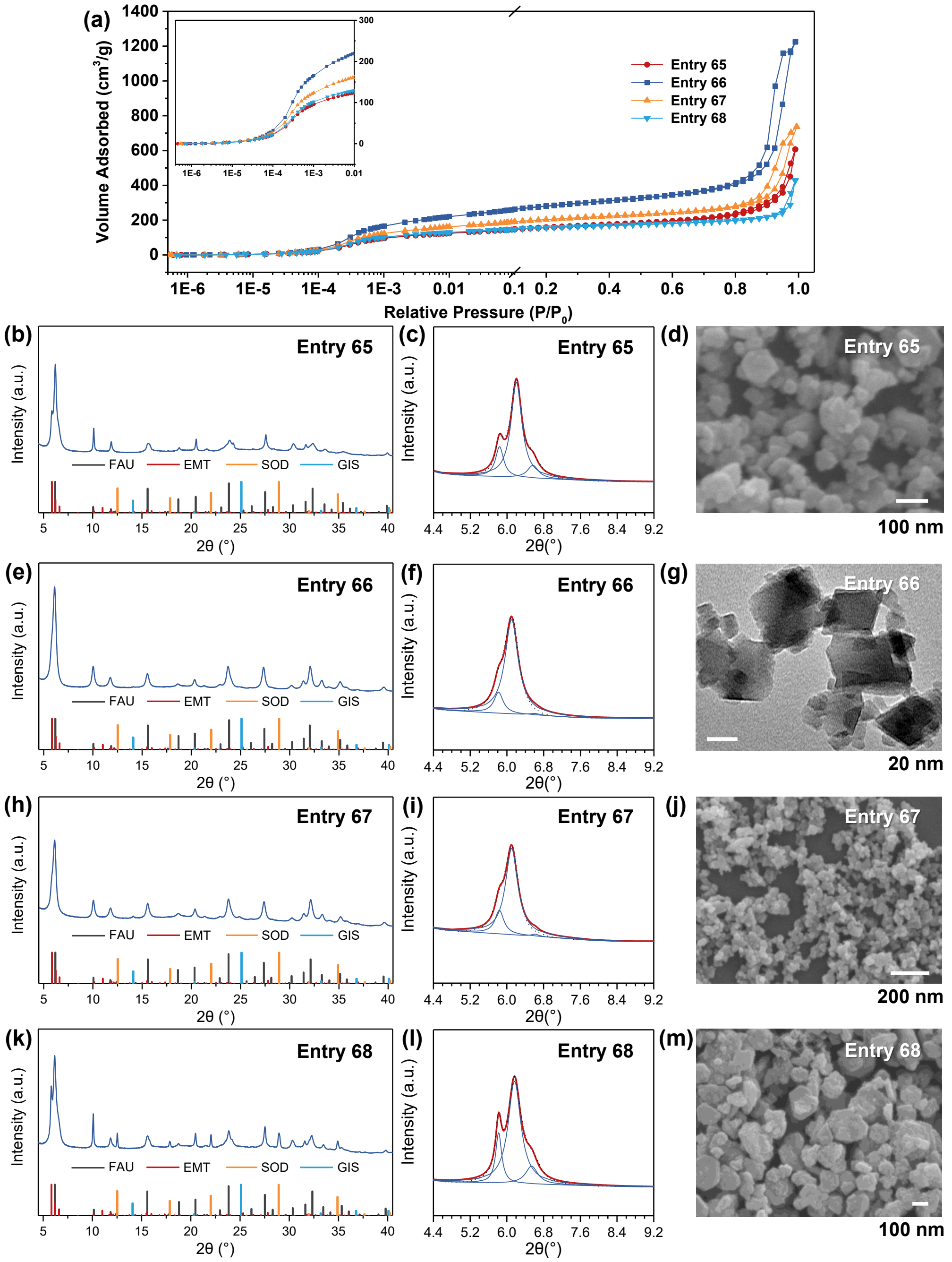

Supplementary Figure 18. Characterization results of faujasite zeolite (FAU and EMT) materials (Entries 65~68). (a) Ar-adsorption isotherms. (b)(e)(h)(k) XRD patterns (plotted for the CuKα wavelength of 1.54059 Å) for Na-FAU zeolites converted from synchrotron XRD patterns. (c)(f)(i)(l) Zoomed-in XRD patterns for the first main diffraction peak used to determine the FAU/(FAU+EMT) ratio. FAU(111) peak is centered at ~ 6.2°, and EMT(100) peak is centered at ~ 5.8°. (d) (j)(m) SEM and (g) TEM images of Na-FAU zeolites.

Supplementary Table 20. Synthesis recipes and corresponding physical properties of faujasite zeolite (FAU and EMT) materials (Entries 69~72).

Entry	SiO_2	Al_2O_3	Na_2O	$H_2O_{initial}$	H_2O_{final}	Si source	Al source	Type of oven	Cryst. Temp. (°C)	Cryst. Time (day)	Si/Al ratio via ICP	Particle Size (nm)	Crystal Size (nm)	FAU/ (FAU + EMT)	uptake value at p/p_0= 0.01 (cm^3/g)
69	12.00	1.00	11.00	214.00	100.00	HS30	Al powder	rotation oven	50	6.00	1.179	72	20.1	0.774	159
70	12.00	1.00	10.00	214.00	100.00	HS30	Al powder	rotation oven	50	6.00	1.291	61	14.7	0.652	118
71	18.00	1.00	18.00	214.00	214.00	NaSi	NaAl	static oven	50	6.00	1.311	605	67.2	0.957	182
72	13.33	1.00	13.33	237.78	111.11	HS30	Al powder	rotation oven	50	6.00	1.103	36	29.0	0.856	192

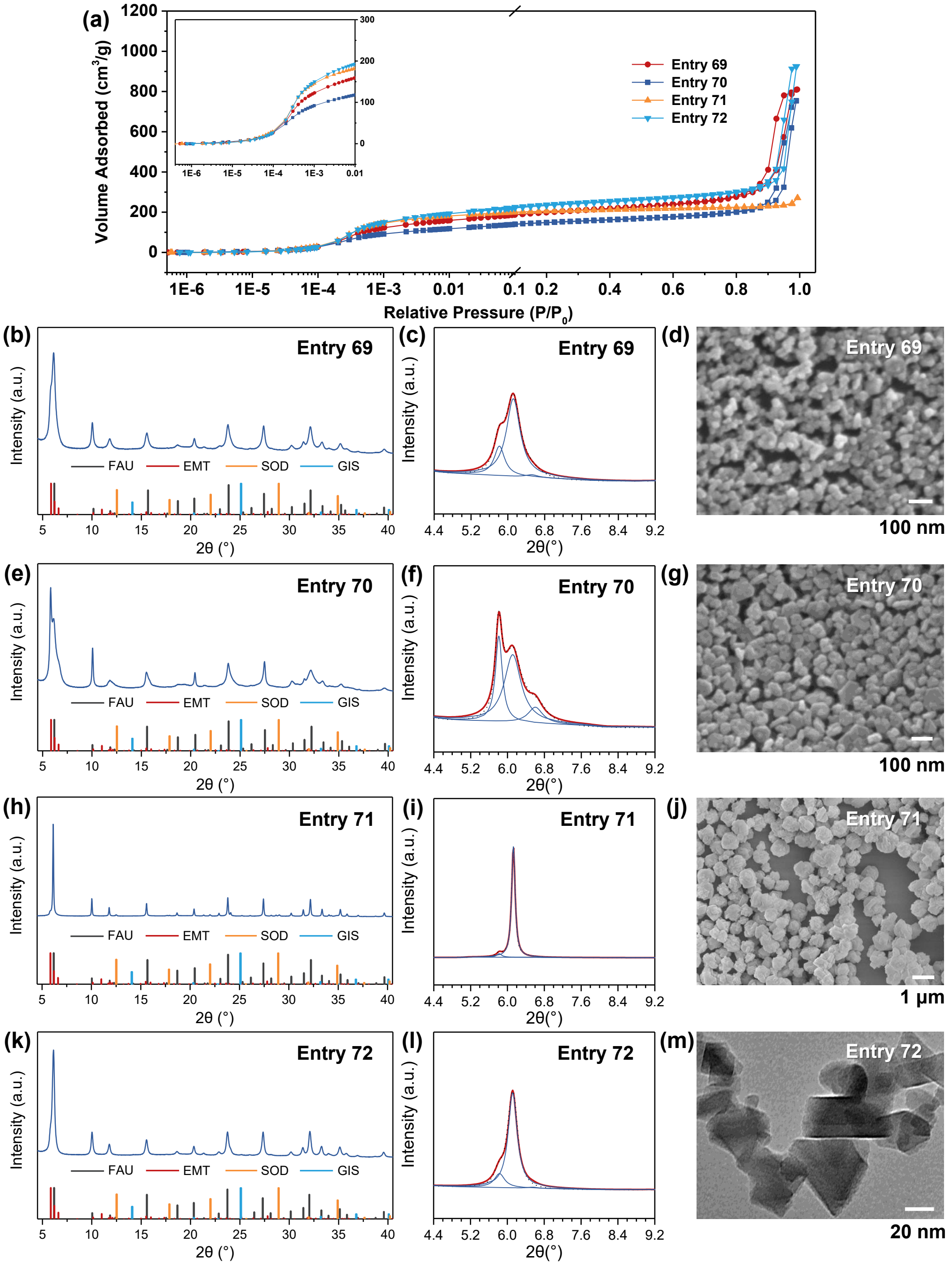

Supplementary Figure 19. Characterization results of faujasite zeolite (FAU and EMT) materials (Entries 69~72). (a) Ar-adsorption isotherms. (b)(e)(h)(k) XRD patterns (plotted for the CuKα wavelength of 1.54059 Å) for Na-FAU zeolites converted from synchrotron XRD patterns. (c)(f)(i)(l) Zoomed-in XRD patterns for the first main diffraction peak used to determine the FAU/(FAU+EMT) ratio. FAU(111) peak is centered at ~ 6.2°, and EMT(100) peak is centered at ~ 5.8°. (d)(g)(j) SEM and (m) TEM images of Na-FAU zeolites.

Supplementary Table 21. Synthesis recipes and corresponding physical properties of faujasite zeolite (FAU and EMT) materials (Entries 73~76).

Entry	SiO_2	Al_2O_3	Na_2O	$H_2O_{initial}$	H_2O_{final}	Si source	Al source	Type of oven	Cryst. Temp. (°C)	Cryst. Time (day)	Si/Al ratio via ICP	Particle Size (nm)	Crystal Size (nm)	FAU/ (FAU + EMT)	uptake value at p/p_0= 0.01 (cm^3/g)
73	12.00	1.00	11.00	196.00	92.00	HS30	Al powder	rotation oven	50	5.00	1.204	91	19.3	0.825	135
74	14.00	1.00	14.00	110.00	110.00	NaSi	NaAl	oil bath	50	2.00	1.196	102	31.3	0.889	181
75	14.00	1.00	14.00	110.00	110.00	NaSi	NaAl	oil bath	50	3.00	1.162	372	56.9	0.982	189
76	14.00	1.00	14.00	110.00	110.00	NaSi	NaAl	oil bath	50	3.00	1.159	204	39.9	0.928	143

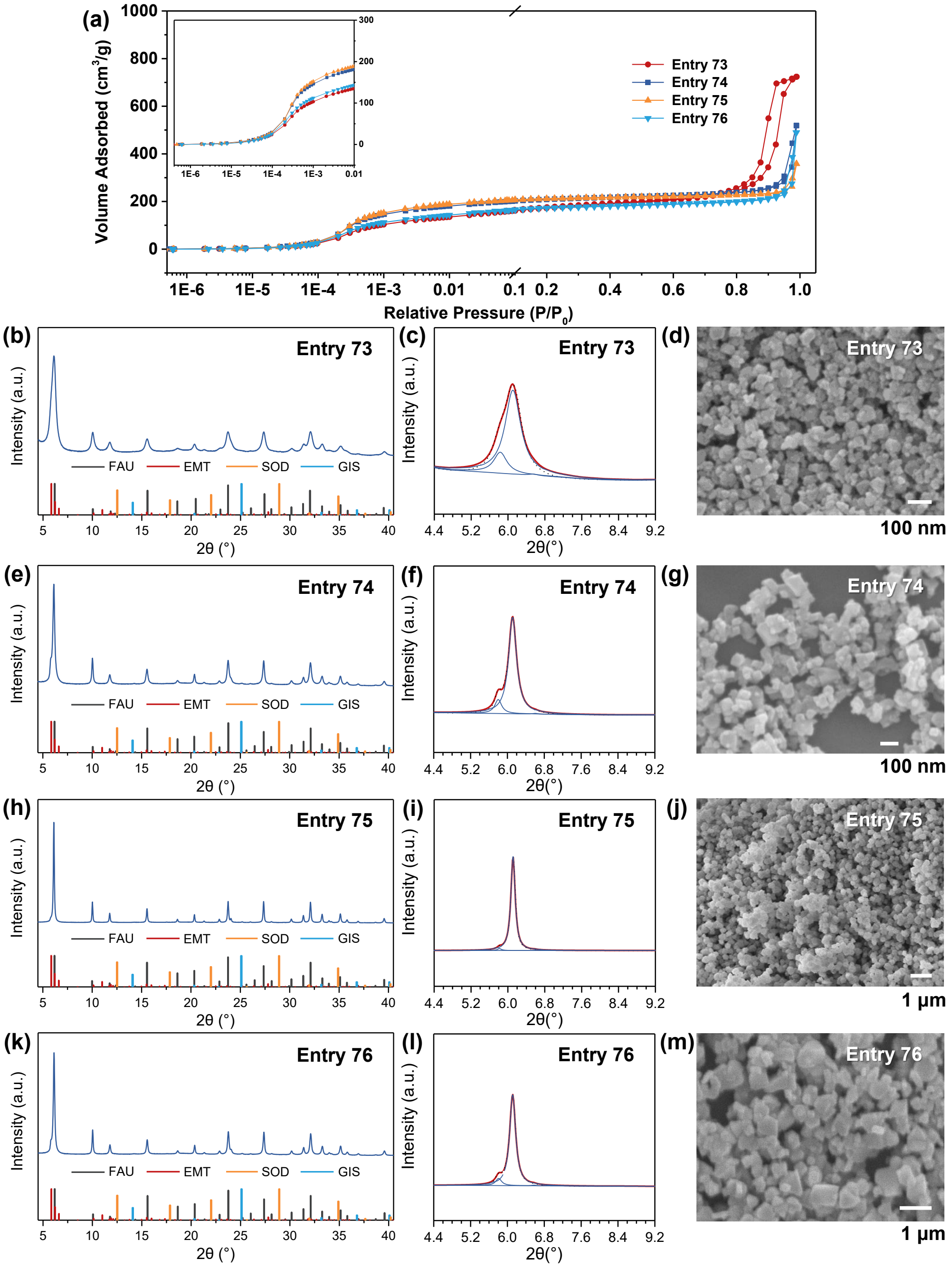

Supplementary Figure 20. Characterization results of faujasite zeolite (FAU and EMT) materials (Entries 73~76). (a) Ar-adsorption isotherms. (b)(e)(h)(k) XRD patterns (plotted for the CuKα wavelength of 1.54059 Å) for Na-FAU zeolites converted from synchrotron XRD patterns. (c)(f)(i)(l) Zoomed-in XRD patterns for the first main diffraction peak used to determine the FAU/(FAU+EMT) ratio. FAU(111) peak is centered at ~ 6.2°, and EMT(100) peak is centered at ~ 5.8°. (d)(g)(j)(m) SEM images of Na-FAU zeolites.

Supplementary Table 22. Synthesis recipes and corresponding physical properties of faujasite zeolite (FAU and EMT) materials (Entries 77~80).

Entry	SiO_2	Al_2O_3	Na_2O	$H_2O_{initial}$	H_2O_{final}	Si source	Al source	Type of oven	Cryst. Temp. (°C)	Cryst. Time (day)	Si/Al ratio via ICP	Particle Size (nm)	Crystal Size (nm)	FAU/ (FAU + EMT)	uptake value at p/p_0= 0.01 (cm^3/g)
77	15.00	1.00	15.00	267.50	125.00	HS30	Al powder	rotation oven	50	6.00	1.174	85	49.4	0.986	209
78	20.00	1.00	20.00	214.00	214.00	NaSi	NaAl	static oven	50	6.00	1.255	475	86.5	0.993	208
79	12.00	1.00	10.00	178.00	83.00	HS30	Al powder	rotation oven	50	5.00	1.245	168	19.6	0.83	114
80	14.00	1.00	14.00	214.00	100.00	NaSi	NaAl	static oven	50	6.00	1.207	150	60.7	0.993	194

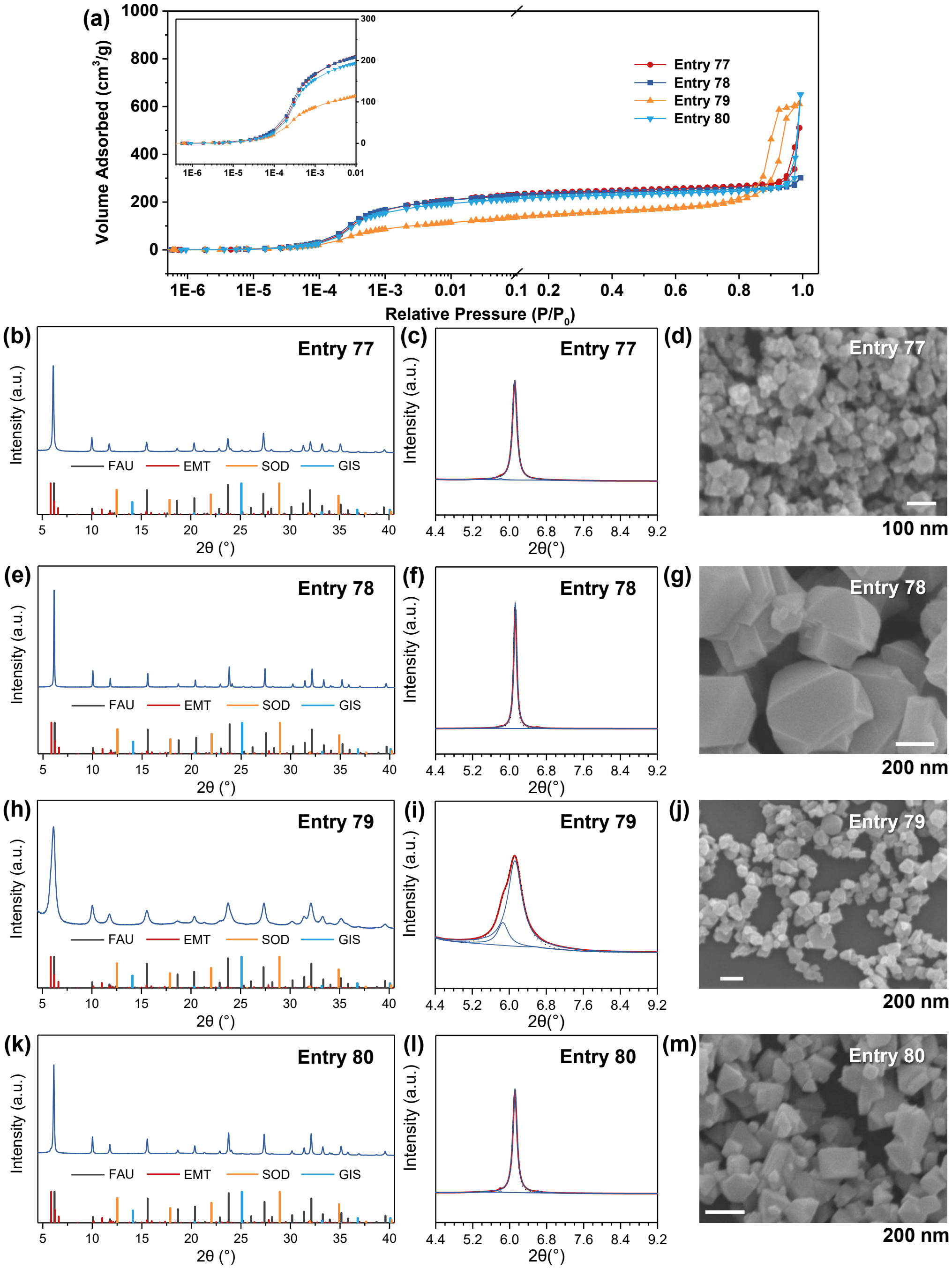

Supplementary Figure 21. Characterization results of faujasite zeolite (FAU and EMT) materials (Entries 77~80). (a) Ar-adsorption isotherms. (b)(e)(h)(k) XRD patterns (plotted for the CuKα wavelength of 1.54059 Å) for Na-FAU zeolites converted from synchrotron XRD patterns. (c)(f)(i)(l) Zoomed-in XRD patterns for the first main diffraction peak used to determine the FAU/(FAU+EMT) ratio. FAU(111) peak is centered at ~ 6.2°, and EMT(100) peak is centered at ~ 5.8°. (d)(g)(j)(m) SEM images of Na-FAU zeolites.

Supplementary Table 23. Synthesis recipes and corresponding physical properties of faujasite zeolite (FAU and EMT) materials (Entries 81~84).

Entry	SiO_2	Al_2O_3	Na_2O	$H_2O_{initial}$	H_2O_{final}	Si source	Al source	Type of oven	Cryst. Temp. (°C)	Cryst. Time (day)	Si/Al ratio via ICP	Particle Size (nm)	Crystal Size (nm)	FAU/ (FAU + EMT)	uptake value at p/p_0= 0.01 (cm^3/g)
81	12.00	1.00	10.00	180.00	80.00	HS30	Al powder	rotation oven	50	4.00	1.308	40	24.5	0.859	196
82	17.14	1.00	17.14	305.71	142.86	HS30	Al powder	rotation oven	50	6.00	1.197	608	53.7	0.916	157
83	14.00	1.00	12.00	214.00	100.00	NaSi	NaAl	oil bath	50	2.00	1.288	103	33.9	0.891	211
84	14.00	1.00	12.00	214.00	100.00	HS30	NaAl	oil bath	50	2.00	1.391	146	50.2	0.955	176

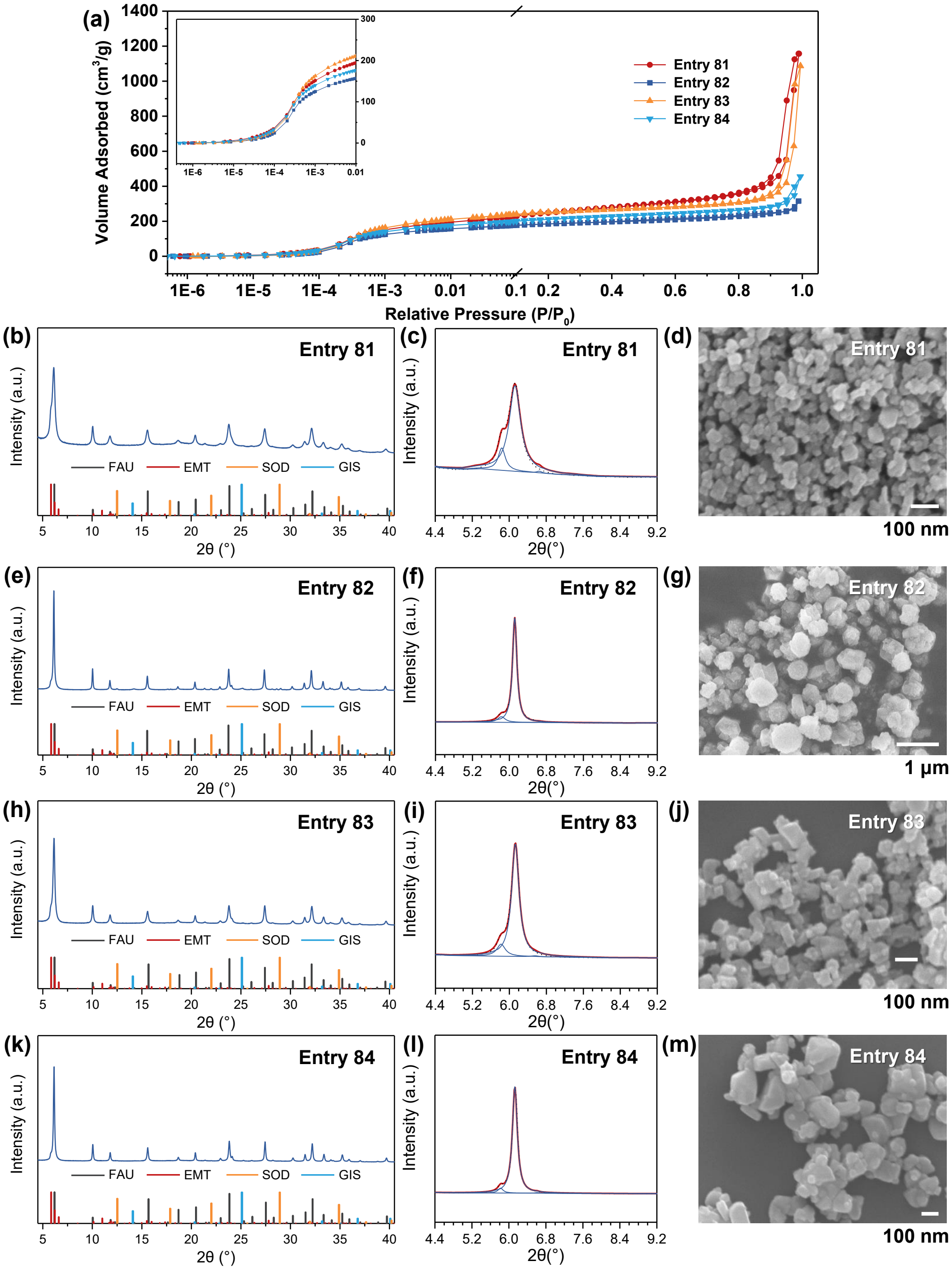

Supplementary Figure 22. Characterization results of faujasite zeolite (FAU and EMT) materials (Entries 81~84). (a) Ar-adsorption isotherms. (b)(e)(h)(k) XRD patterns (plotted for the CuKα wavelength of 1.54059 Å) for Na-FAU zeolites converted from synchrotron XRD patterns. (c)(f)(i)(l) Zoomed-in XRD patterns for the first main diffraction peak used to determine the FAU/(FAU+EMT) ratio. FAU(111) peak is centered at ~ 6.2°, and EMT(100) peak is centered at ~ 5.8°. (d)(g)(j)(m) SEM images of Na-FAU zeolites.

Supplementary Table 24. Synthesis recipes and corresponding physical properties of faujasite zeolite (FAU and EMT) materials (Entries 85~88).

Entry	SiO_2	Al_2O_3	Na_2O	$H_2O_{initial}$	H_2O_{final}	Si source	Al source	Type of oven	Cryst. Temp. (°C)	Cryst. Time (day)	Si/Al ratio via ICP	Particle Size (nm)	Crystal Size (nm)	FAU/ (FAU + EMT)	uptake value at p/p_0= 0.01 (cm^3/g)
85	14.00	1.00	12.00	214.00	100.00	HS30	Al foil1	oil bath	50	2.00	1.345	146	39.6	0.939	163
86	14.00	1.00	12.00	214.00	100.00	NaSi	NaAl	static oven	50	6.00	1.310	70	31.8	0.889	169
87	12.00	1.00	9.00	160.00	75.00	HS30	Al powder	rotation oven	50	5	2.032	392	80.6	0.99	145
88	12.00	1.00	6.00	107.00	50.00	HS30	Al powder	static oven	70	4.00	2.289	300	57.6	0.985	172

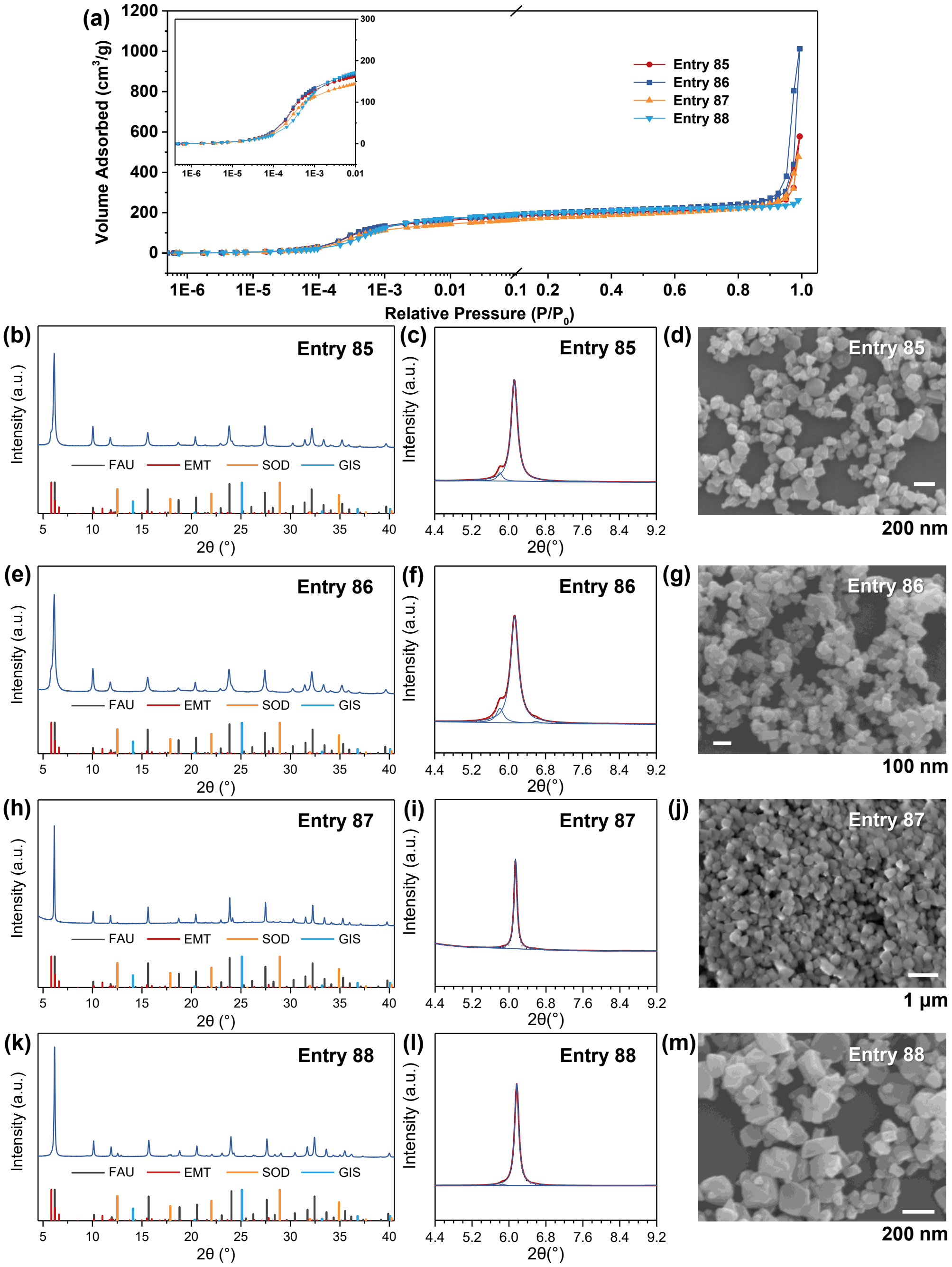

Supplementary Figure 23. Characterization results of faujasite zeolite (FAU and EMT) materials (Entries 85~88). (a) Ar-adsorption isotherms. (b)(e)(h)(k) XRD patterns (plotted for the CuKα wavelength of 1.54059 Å) for Na-FAU zeolites converted from synchrotron XRD patterns. (c)(f)(i)(l) Zoomed-in XRD patterns for the first main diffraction peak used to determine the FAU/(FAU+EMT) ratio. FAU(111) peak is centered at ~ 6.2°, and EMT(100) peak is centered at ~ 5.8°. (d)(g)(j)(m) SEM images of Na-FAU zeolites.

Supplementary Table 25. Synthesis recipes and corresponding physical properties of faujasite zeolite (FAU and EMT) materials (Entries 89~92).

Entry	SiO_2	Al_2O_3	Na_2O	$H_2O_{initial}$	H_2O_{final}	Si source	Al source	Type of oven	Cryst. Temp. (°C)	Cryst. Time (day)	Si/Al ratio via ICP	Particle Size (nm)	Crystal Size (nm)	FAU/ (FAU + EMT)	uptake value at p/p_0= 0.01 (cm^3/g)
89	12.00	1.00	3.50	160.00	160.00	AS40	NaAl	static oven	100	9.00	3.016	1347	91.8	0.993	214
90	12.00	1.00	3.50	160.00	160.00	AS40	NaAl	static oven	100	12.00	3.018	1350	89.9	0.992	216
91	12.00	1.00	3.50	160.00	160.00	AS40	NaAl	static oven	100	13.00	3.528	1808	92.5	0.991	157
92	13.00	1.00	3.00	160.00	160.00	NaSi	NaAl	static oven	100	13.00	3.15	1685	83.9	0.995	179

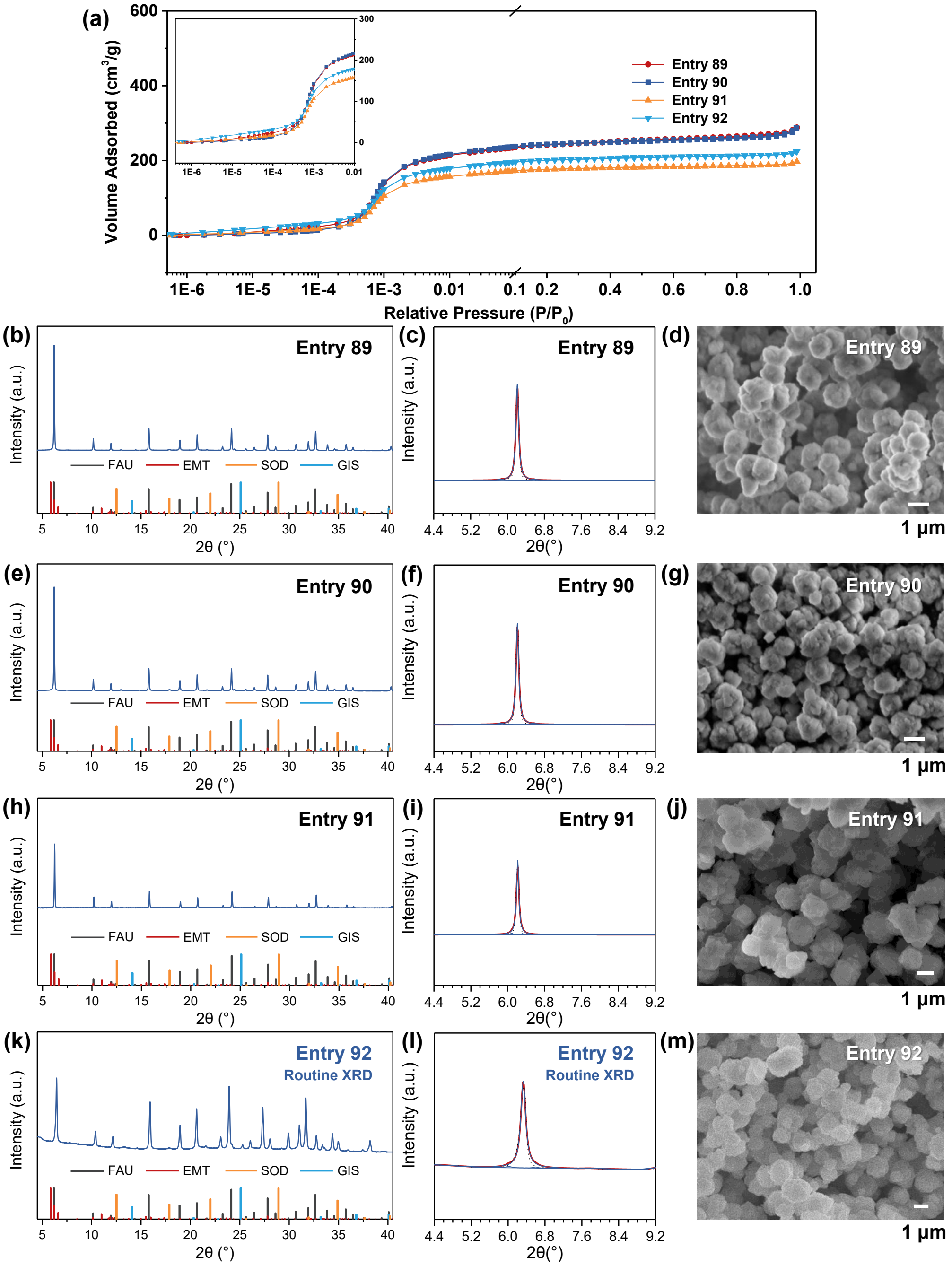

Supplementary Figure 24. Characterization results of faujasite zeolite (FAU and EMT) materials (Entries 89~92). (a) Ar-adsorption isotherms. (b)(e)(h)(k) XRD patterns (plotted for the CuKα wavelength of 1.54059 Å) for Na-FAU zeolites converted from synchrotron XRD patterns. (c)(f)(i)(l) Zoomed-in XRD patterns for the first main diffraction peak used to determine the FAU/(FAU+EMT) ratio. FAU(111) peak is centered at ~ 6.2°, and EMT(100) peak is centered at ~ 5.8°. (d)(g)(j)(m) SEM images of Na-FAU zeolites.

S3. Method Description of Machine Learning

S3.1. Diffusion Maps and Geometric Harmonics

The manifold learning technique Diffusion Maps, can be used in two ways: it reduces the embedding dimensions of a (finite) data set, $\boldsymbol{X}=\{\boldsymbol{x}_i\}_{i=1}^{N}$ where $\boldsymbol{x}_i \in \mathbb{R}^m$ are data points sampled on a manifold, $\mathcal{M}$, and it also extends functions defined on the manifold/data set by building a functional basis termed *Geometric Harmonics*.[1,2] Here we use the latter functionality of Diffusion Maps to build mappings between the inputs (synthesis conditions) and the outputs (structural characteristics).

Given data $\boldsymbol{X}$, sampled from manifold $\mathcal{M}$ and a function f, defined on the data, Geometric Harmonics *extends* f *off-sample,* for $\boldsymbol{x}_{\text{new}} \notin \boldsymbol{X}$. To obtain the basis functions that will allow us to *approximate* any function of interest, the algorithm first computes an affinity matrix $\boldsymbol{A}$:

$$A(\boldsymbol{x}_i, \boldsymbol{x}_j) = e^{\frac{-\left\|x_i - x_j\right\|^2}{2\varepsilon}} \quad (1)$$

where $\|\cdot\|$ denotes the ℓ^2 norm. The scale hyperparameter $\varepsilon > 0$ regulates the rate of decay of the kernel: for small values of ε, only points that are close to each other are considered as connected in $\boldsymbol{A}$, since distant points will have $A_{ij} \approx 0$.

This symmetric and positive semidefinite matrix $\boldsymbol{A}$ has non-negative eigenvalues ($\lambda_1 \geq \lambda_2 \geq \cdots \geq \lambda_N \geq 0$) and a corresponding set of orthonormal eigenvectors ($\psi_1, \psi_2, \ldots, \psi_N$) that form a basis of the functional space in which we can project and subsequently extend the function of interest f. The projection step, described in the Supplementary Equation 2, can be thought of as training or *calibrating* our model by adjusting the parameters of a regression model for f. In the extension step, Supplementary Equation 3, we use the trained model to predict the value of f *for new unseen points*. This is achieved via the Nyström Extension,[3,4] Supplementary Equation 4, in which the extended value of Ψ for an out-of-sample point $\boldsymbol{x}_{new}$ is a weighted average of the corresponding values on the original set $\boldsymbol{X}$. In general, the more eigenfunctions we retain, the better the result; yet numerically, the extension procedure of Supplementary Equation 4 may become ill-conditioned since one divided by singular values

that can become arbitrarily close to zero. Therefore, it is important to judiciously select the set of truncated eigenvectors, for $\delta > 0, S_\delta = \{\alpha : \sigma_\alpha > \delta\sigma_1\}$.

In the general setting, assuming a truncated set of eigenfunctions was retained, we project f evaluated on our training set:

$$f \mapsto P_\delta f = \sum_{\alpha \in S_\delta} \langle f, \psi_\alpha \rangle \psi_\alpha, \ (2)$$

where $\langle \cdot,\cdot \rangle$ denotes the inner product. The extension of f for $\boldsymbol{x}_{new} \notin \boldsymbol{X}$ is defined as:

$$(Ef)(\boldsymbol{x}_{new}) = \sum_{\alpha \in S_\delta} \langle f, \psi_\alpha \rangle \Psi_\alpha(\boldsymbol{x}_{new}) \ (3)$$

where

$$\Psi_\alpha(\boldsymbol{x}_{new}) = \lambda_\alpha^{-1} \sum_{i=1}^{N} A(\boldsymbol{x}_{new}, \boldsymbol{x}_i) \psi_\alpha(\boldsymbol{x}_i) \ (4).$$

Here, N is the number of data points used in the GH computations, $\psi_\alpha(x_i)$ is the ith component of the diffusion map eigenvector ψ_α and Ψ_α is α^{th} extended Geometric Harmonics function and λ_a is the ath eigenvalue.

The aforementioned algorithm, by first projecting and subsequently extending a function, allows us to build a data-driven model of relations between inputs and outputs whenever closed form expressions are not available. A visualizable example is illustrated in Supplementary Figure 25 that shows the ability of GH to learn a function of interest and generalize for new unseen data points. It is worth mentioning that, even though it is possible to use a single basis to learn multiple functions, $\boldsymbol{F} = \{f_1, f_2, \cdots\}$, simultaneously, we chose to build a distinct basis for each function of interest f_i. This allowed us to select the hyperparameters ε and the number of chosen truncated eigenvectors, N_δ, based on the complexity of each function. To the best of our knowledge, a *priori* optimal selection of hyperparameters for any function of interest f does not exist. Common-sense recipes for hyperparameter selection are discussed in the literature without definitive theory. We used cross validation for the selection of those hyperparameters. Our goal through this cross-validation is to select parameters that allow the model to *learn* the function of interest based on our training set but also to generalize as well as possible for unseen data points.

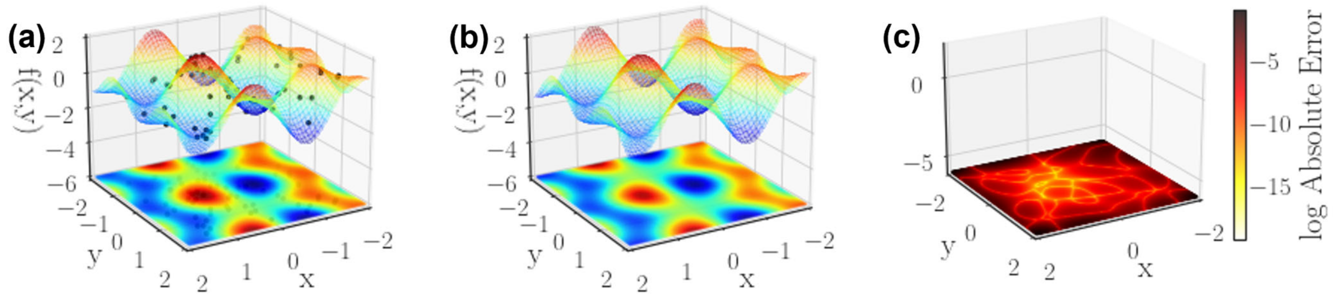

Supplementary Figure 25. (a) True function with observed data (black points). (b) Interpolated Function with Geometric Harmonics. (c) The logarithm of the absolute error.

Beyond extending a function defined on $\boldsymbol{X}$, Geometric Harmonics can also help compute the gradient of the function in terms of the original variables. Symbolic differentiation of Supplementary Equations 2 and 3 gives a closed form expression of the gradient of f in term of the independent variables. Being able to compute the gradient of a data-driven function f allows to perform gradient ascent/descent in the input space. Such a scheme can suggest new experimental synthesis conditions that improve a particular structural characteristic of interest.

Geometric Harmonics can also help compute the gradient of the function in terms of the original variables. Symbolic differentiation of Supplementary Equations 2 and 3 gives a closed-form expression of the gradient of f in terms of the independent variables, Supplementary Equation 5. Apart from symbolic differentiation, the gradient of a function f can be computed with finite differences or automatic differentiation. Being able to compute the gradient of a data-driven function allows performing gradient ascent/descent in the input. Such a scheme can suggest new experimental synthesis conditions that improve a particular structural characteristic of interest.

$$\nabla f(\boldsymbol{x}_{new}) = \sum_{\alpha \in S_\delta} \frac{\langle f, \psi_\alpha \rangle}{\lambda_\alpha} \sum_{i=1}^{M \le N} -\frac{(x_{new} - x_i)}{\varepsilon} A(\boldsymbol{x}_{new}, \boldsymbol{x}_i) \psi_\alpha(\boldsymbol{x}_i) \quad (5)$$

S3.2. Neural Networks

In this paper, we use feed-forward neural networks (multilayer perceptrons) in which the information flows from the input to the output without recurrent connections. The network consists of an assembly of simple processing elements, *nodes* also known as neurons.[5,6] In Supplementary Figure 26 a feed forward neural network with five layers is shown. The layers are categorized as the input, hidden, or output, which in the figure here have two nodes, three nodes, and one node, respectively.[6]

The output of a single neuron is given by computing a non-linear function (g) of the weighted sum of outputs of previous layers and adding a bias term:

$$\alpha_i^{out} = g\left(\sum_j W_{ij}\alpha_j^{in} + b_i\right), (6)$$

where α_j^{in} is the output of the jth node of the previous layer, and W_{ij} and b_i are the parameters for this neuron. Selection of parameter values is accomplished by *training* the network, here with (stochastic) gradient descent, minimizing a loss function, with derivatives evaluated through automatic differentiation. Given a trained neural network, we generalize to unseen values of the inputs by *evaluating* the corresponding outputs.

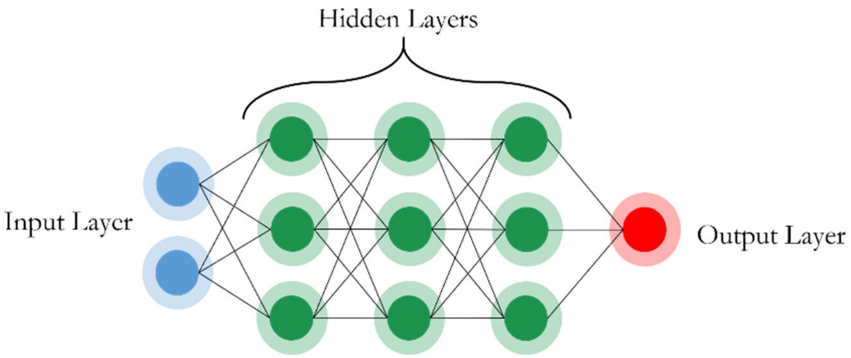

Supplementary Figure 26. A feed forward Neural Network.

S3.3. Gaussian Process Regression

Another established method of constructing surrogate models for fitting experimental data is Gaussian process regression (GPR). Gaussian processes model a function as a collection of jointly normal random variables that describe one's knowledge about $f(\boldsymbol{x})$ at each point in the function's domain. These variables are correlated according to a kernel function,

$$k(\boldsymbol{x}, \boldsymbol{x}') = \mathrm{Cov}[f(\boldsymbol{x}), f(\boldsymbol{x}')] \quad (7)$$

which is chosen by the user so that key properties of the model (*e.g.*, differentiability, periodicity) match those expected of the true function.[7] The most common family of kernels is the squared exponential, which takes the form

$$k(\boldsymbol{x}, \boldsymbol{x}') = \sigma^2 \exp\left[\frac{-\|x - x'\|^2}{2\ell^2}\right] \quad (8)$$

where the hyperparameters $\sigma^2, \ell > 0$ represent signal variance and length scale, respectively. If we observe paired input-output data $\mathcal{D} = \{\boldsymbol{x}_i, \boldsymbol{y}_i\}_{i=1}^{N}$, GPR uses conditional probability to predict the function value $y' = f(\boldsymbol{x}')$ at a new input $\boldsymbol{x}$'. Such predictions are expressed as Gaussian distributions[7] with

$$\mathbb{E}[y' \mid \boldsymbol{x}', \mathcal{D}] = \sum_{i=1}^{N} \sum_{j=1}^{N} k(\boldsymbol{x}', \boldsymbol{x}_i) K_{ij} y_j \quad (9)$$

$$\mathbb{V}[y' | \boldsymbol{x}', \mathcal{D}] = k(\boldsymbol{x}', \boldsymbol{x}') - \sum_{i=1}^{N} \sum_{j=1}^{N} k(\boldsymbol{x}', \boldsymbol{x}_i) K_{ij} k(\boldsymbol{x}_j, \boldsymbol{x}') \quad (10)$$

where the Gram matrix $K \in \mathbb{R}^{n \times n}$ has entries $K_{ij} = k(\boldsymbol{x}_i, \boldsymbol{x}_j)$. The mean given in (Supplementary Equation 9) can be used as the value predicted by the surrogate model, with the variance in (Supplementary Equation 10) serving as a measure of the prediction accuracy.[8] Supplementary Figure 27 illustrates a simple example in one dimension.

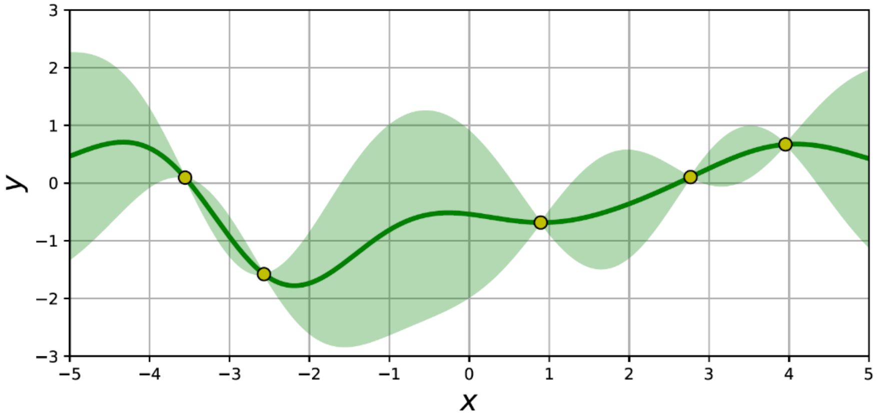

Supplementary Figure 27. Given the observed input-output pairs (yellow circles), GPR computes a predicted distribution for each point in the domain. The curve and shaded region represent the mean and 95% CI as functions of $x \in [-5,5]$. The variance is smaller near observed x_i because the squared exponential kernel used here stipulates high correlation between points located at a close distance.

One advantage of Gaussian process regression is that the variance estimate (Supplementary Equation 10) provides a measure of uncertainty in the model's prediction. For new inputs $\boldsymbol{x}_{\text{new}}$ that are close to training points, the model can be reasonably confident that the predicted mean (Supplementary Equation 9) is close to the true value $y_{\text{new}} = f(\boldsymbol{x}_{\text{new}})$. As we move further away from points in the training set, the information that these observations provide becomes less helpful for prediction, and the model accounts for this by assigning greater variance to predictions made in such regions of the domain. Supplementary Figure 38 illustrates this phenomenon for two different "slices" through our data.

Hyperparameter values are determined by maximizing the log-likelihood of the observed output vector. This takes advantage of the model assumption that output values are normally distributed. Since all relevant distributions are assumed to be Gaussian, the log-likelihood is given by

$$\begin{gathered} \log p(\boldsymbol{y} \mid X, \boldsymbol{\theta}) = -\tfrac{1}{2}\left[\boldsymbol{y}^{\top} K_{\boldsymbol{\theta}}^{-1} \boldsymbol{y} + \log|K_{\boldsymbol{\theta}}| + n \log 2\pi\right] \\ K_{\boldsymbol{\theta}} = K(X, X; \boldsymbol{\theta}) + \sigma_n^2 I \end{gathered} \tag{11}$$

where $K(X, X; \boldsymbol{\theta})$ is the Gram matrix $K_{ij} = k(\boldsymbol{x}_i, \boldsymbol{x}_j; \boldsymbol{\theta})$, implicitly dependent on hyperparameters $\boldsymbol{\theta}$, and $\sigma_n^2 \geq 0$ is an (optional) additional hyperparameter used when the observed outputs are assumed to be corrupted by independent $\mathcal{N}(0, \sigma_n^2)$ noise.[7] Even in a case without noise, it may still prove helpful to use a small positive value for σ_n^2 (say, 10^{-10}) to ensure that $K_{\boldsymbol{\theta}}$ is numerically positive definite and improve the stability of computing its inverse.

More involved approaches exist for cases where the measurement noise is not of uniform variance.[9] Here, one can use a diagonal matrix instead of $\sigma_n^2 I$ when updating $K_{\boldsymbol{\theta}}$. In this case, the diagonal entries are true or estimated values of the noise variance by which corresponding output observations are corrupted. Suppose, however, that this noise variance is a function, $\sigma_n^2(\boldsymbol{x})$, and we additionally have estimates $s_i^2 \approx \sigma_n^2(\boldsymbol{x})$ for each observation. One approach to this heteroscedastic case, described by Kersting et al.,[10] first fits an ordinary (homoscedastic) GP model for predicting $h(\boldsymbol{x}) = \log \sigma_n^2(\boldsymbol{x})$, then uses predictions from this model to update the Gram matrix. This requires using two kernels, one to learn the noise level and the other for (mean) value prediction.

S4. Correlations of Experimental and Predicted Physical Properties of Faujasite Zeolites

We characterize the outputs of each synthesis experiment as functions of nine input quantities:

- Four for mixture composition (Na_2O/Al_2O_3, SiO_2/Al_2O_3, H_2O_{final}/Na_2O, and $H_2O_{final}/H_2O_{initial}$), expressed as a molar ratio of silica, alumina, sodium hydroxide, and (initial and final) water;
- Crystallization time;
- Crystallization temperature;
- Source of silica (NaSi,HS30 and AS40);
- Source of alumina (NaAl, Al powder, and Al foil);

- Oven type used for the synthesis (static oven, rotation oven, and oil bath).

Six of these variables (the four for mixture composition, time, and temperature) are numerical, whereas the other three are categorical. Each of the categorical variables has three levels, so we represent them quantitatively by mapping each level to one vertex of an equilateral triangle in the plane, i.e., they are embedded in a 2D space. Consequently, the input for each experiment is represented as a vector in $6 + 2 \times 3 = 12$-dimensional space.

S4.1. Input Preprocessing Schemes. For regression tasks, scaling or otherwise transforming the data can improve performance.[11] Therefore, we tested and compared two different preprocessing strategies for our inputs. Our first approach is to normalize the input data: the training and test data are normalized by subtracting the empirical mean and dividing by the empirical standard deviation of the training data (we refer to this as the "center" approach). Our second approach is to rescale the data such that the coordinates corresponding to numerical inputs fall in the range $0 \leq x_i \leq 1$ (we refer to this as the "rescaled" approach). The output quantities were also normalized, after some additional transformations that we found to improve performance. Particle size, crystal size, and uptake value varied over multiple orders of magnitude, so we first applied a logarithm to these outputs and exponentiated the resulting predictions to recover physically meaningful values. Since the FAU fraction is constrained to the range $0 \leq y \leq 1$, we first applied the inverse sigmoid

$$y \mapsto \ln\left(\frac{y}{1-y}\right) \quad (12)$$

before training, and recovered true predictions via

$$z \mapsto \frac{1}{1+e^{-z}} \quad (13)$$

Using this transformation ensures that all predicted values of FAU fraction are physically meaningful. Lastly, for each output we employed cross-validation when selecting hyperparameter values for our surrogate function model, so as to avoid overfitting and achieve better generalization to new inputs.[11]

S4.2. Geometric Harmonic Models

We select the hyperparameters for Geometric Harmonics by taking into consideration a *good bias-variance trade off*. *Bias* refers to error that is introduced by approximating a complicated function with a much simpler one.

Variance refers to the amount by which a surrogate function f^* will change if we estimate it by using a different training set. As a rule of thumb, as more complexity is added to the model variance increases, bias decreases, and the data set is overfitted. Conversely, as complexity decreases bias increases, and variance decreases. A model with good test set performance requires a trade-off between low bias and low variance.

We first separated our data into a training set and a test set. The same training and test points were used for all the different outputs (structural properties) if they were available. The test data points were selected to be different than any of the training set in order to ensure that the generalization performance of the model is not inflated. In addition, the training data set was further partitioned to be used for cross-validation.[11]

Cross-validation is employed as an estimation of the ability of a model to perform predictions for new unseen information. In *K-fold* cross validation the training set is randomly partitioned into K subsets and each of those subsets is being used as validation sets of the model trained on the remaining *K-1* subsets.[11] For each of these subsets the mean squared error (MSE) is computed. By averaging over the K different values of MSE we obtain an estimate of how well our model generalizes for unseen data points. The hyperparameters that give the lower value of this averaged MSE are selected as the "optimal" hyperparameters for the entire training set. The ability of our model to perform predictions on the test set can now be evaluated. For this *K-fold* cross validation scheme, several K values were tried. We tested our model for values of $K = 5, K = 10$ and $K = N - 1$ (leave one out) where N is the number of data points in our training set. As K becomes smaller, the number of subsets decrease; this means that the total number of entries used in the K-1 subsets decrease too (since the number of entries in the subset that is used for validation increases). The model learns based on fewer observations and therefore it might *overestimate* the MSE error. This *overestimation* might lead in increase of the bias. Letting $K = N - 1$ we get an almost unbiased estimation of the error, since almost all the training points are being used in each estimation of the MSE. The leave one out approach, however, tends to have higher variance than the *K-fold* cross validation.[12] Empirically, the values of $K = 5$ and $K = 10$ are considered as values that balance excessively high bias and very high variance.[12]

<u>*Nine GH models were used for Si/Al ratio prediction.*</u> They include the two different preprocessing techniques (normalized and rescaled) and the three Cross Validations Schemes (six models: normalized 5-CV, normalized 10-

CV, normalized LOOCV (for leave-one-out cross validation), rescaled 5-CV, rescaled 10-CV, rescaled LOOCV). In addition to that, for the Si/Al ratio GH three more models were trained also without the categorical variables by using the rescaling preprocessing and the three cross-validation schemes (three models: rescaled 5-CV, 10-CV, LOOCV (w/o categorical)). Several additional models were trained to predict the five system outputs for an overall total of 44 models, and details regarding these 44 models are listed as follows:

(1) Results developed from the model "rescaled 10-CV" are reported at Supplementary Figure 28 (a)-(f), and results developed from the model "rescaled LOOCV (w/o categorical)" are reported at Supplementary Figure 36 (a)-(f).

(2) For Si/Al we trained 5-CV, 10-CV, LOOCV by normalizing and rescaling the inputs, the results are reported at Supplementary Figure 29 (a)-(f).

(3) For Si/Al we trained 5-CV, 10-CV, LOOCV by rescaling the inputs. The categorical variables for the Si and Al sources and the type of the oven are not included as features in this case. The results are reported at Supplementary Figure 35 (a)-(c).

(4) For the logarithm of the Particles Size, we trained 5-CV, 10-CV, LOOCV by normalizing and rescaling the inputs, the results are reported at Supplementary Figure 30 (a)-(f).

(5) For the logarithm of the Crystal Size, we trained 5-CV, 10-CV, LOOCV by normalizing and rescaling the inputs, the results are reported at Supplementary Figure 31 (a)-(f). Please note that in this case data points with Crystal Size smaller than 60 were used to train and test the models.

(6) For the FAU/(FAU+EMT) we trained 5-CV, 10-CV, LOOCV by normalizing and rescaling the inputs, the results are reported at Supplementary Figure 32 (a)-(f).

(7) For the Uptake Value we trained 5-CV, 10-CV, LOOCV by normalizing and rescaling the inputs, the results are reported at Supplementary Figure 33 (a)-(f).

(8) For the logarithm of Particle Size to Crystal Size we trained 5-CV, 10-CV, LOOCV by normalizing and rescaling the inputs, the results are reported at Supplementary Figure 34 (a)-(f). In this case also data points with Crystal Size smaller than 60 were used to train and test the models.

S4.3. Neural Network Models

Two NN models were trained to predict Si/Al (by using the 12-dimensional input space), and the two **preprocessing schemes (two models: neural network center, and neural network rescaled).**

S4.4. Gaussian Process Regression Models

For the Gaussian Process Regression, normalization was used as preprocessing, and the hyperparameters were selected based on the log-likelihood estimation. Since for the ICP data for Si/Al ratio are subject to a relative error of ca. 1.5% (see Supplementary Table 27) of the observed value, this "noise" seems to be relative to the measurement, and not a constant across measurements. Therefore, a standard GPR model may not be fully appropriate for our analysis. To address this issue, we also performed heteroscedastic GPR and, for each experiment in our training set, took 1.5% of the corresponding Si/Al value as the estimated noise standard deviation. The heteroscedastic GPR incorporates this information into its predictive distribution for any given input conditions.

Five different GPR models were trained using: (a) diagonal training covariance with Radial Basis Function (RBF) kernel (b) diagonal training covariance with Matérn kernel (c) heteroscedastic GPR with two Matérn kernels (d) heteroscedastic GPR with two RBF kernels (e) heteroscedastic GPR with a Matérn kernel for the mean and a RBF kernel for the noise.

The Matérn(0.5) kernel is given by the following equation:

$$k_M(\boldsymbol{x}, \boldsymbol{x}') = \sigma^2 \exp\left[-\frac{\|\boldsymbol{x}-\boldsymbol{x}'\|}{\ell}\right] \quad (14)$$

The RBF is a limiting case of the Matérn kernel family and represents functions that are infinitely differentiable. The Matérn(0.5) kernel, on the other hand, represents functions that are continuous but not differentiable.

S4.5. Presentation of Results

For each output of interest, we report the obtained predictions of the model (for the training set (blue points), testing set (red points), and prediction set after optimization (green points)). The optimum correlations generated from the GH algorithm "rescaled 10-CV" are provided in Fig. 2 in the main text, and the details with entry numbers are provided in Supplementary Figure 28. Details of comparison for the five outputs (Si/Al ratio, particle size, crystal size, FAU/(FAU + EMT) ratio, Ar-sorption) and the ratio of particle size over crystal size among six different

Geometric Harmonics algorithms ((i) normalized 5-CV, (ii) normalized 10-CV, (iii) normalized LOOCV, (iv) rescaled 5-CV, (v) rescaled 10-CV, (vi) rescaled LOOCV) are given in Supplementary Figures 29 to 34.

Additional algorithms are also used to investigate predicted Si/Al ratio: Supplementary Figure 35 includes Si/Al for (i) rescaled 5-CV (w/o categorical), (ii) rescaled 10-CV (w/o categorical), (iii) rescaled LOOCV (w/o categorical), (iv) neural networks center, (v) neural networks rescaled. Supplementary Figure 36 includes the results developed from the model "rescaled LOOCV (w/o categorical)".

Supplementary Figure 37 compares GPR predictions to the true Si/Al values for five variations of GPR model: (a) Diagonal Gram matrix update, RBF, (b) Diagonal Gram matrix update, Matérn(0.5), (c) Fully heteroscedastic, RBF for mean, RBF for noise level, (d) Fully heteroscedastic, Matérn(0.5) for mean, RBF for noise level, (e) Fully heteroscedastic, Matérn(0.5) for mean, Matérn(0.5) for noise level. In each case, we plot both the mean prediction and error bars representing a 95% confidence interval, as computed from the predicted variance.

Supplementary Figure 38 uses two different "slices" through our data to illustrate that as we move further away from points in the training set, the information that these observations provide becomes less helpful for prediction, and the model accounts for this by assigning greater variance to predictions made in such regions of the domain.

Supplementary Figure 39 presents supplementary predictions for unseen experiments to exceed Si/Al = 3 using GPR with Matérn (0.5) kernel and with GH ("the rescaled LOOCV (w/o categorical)" model).

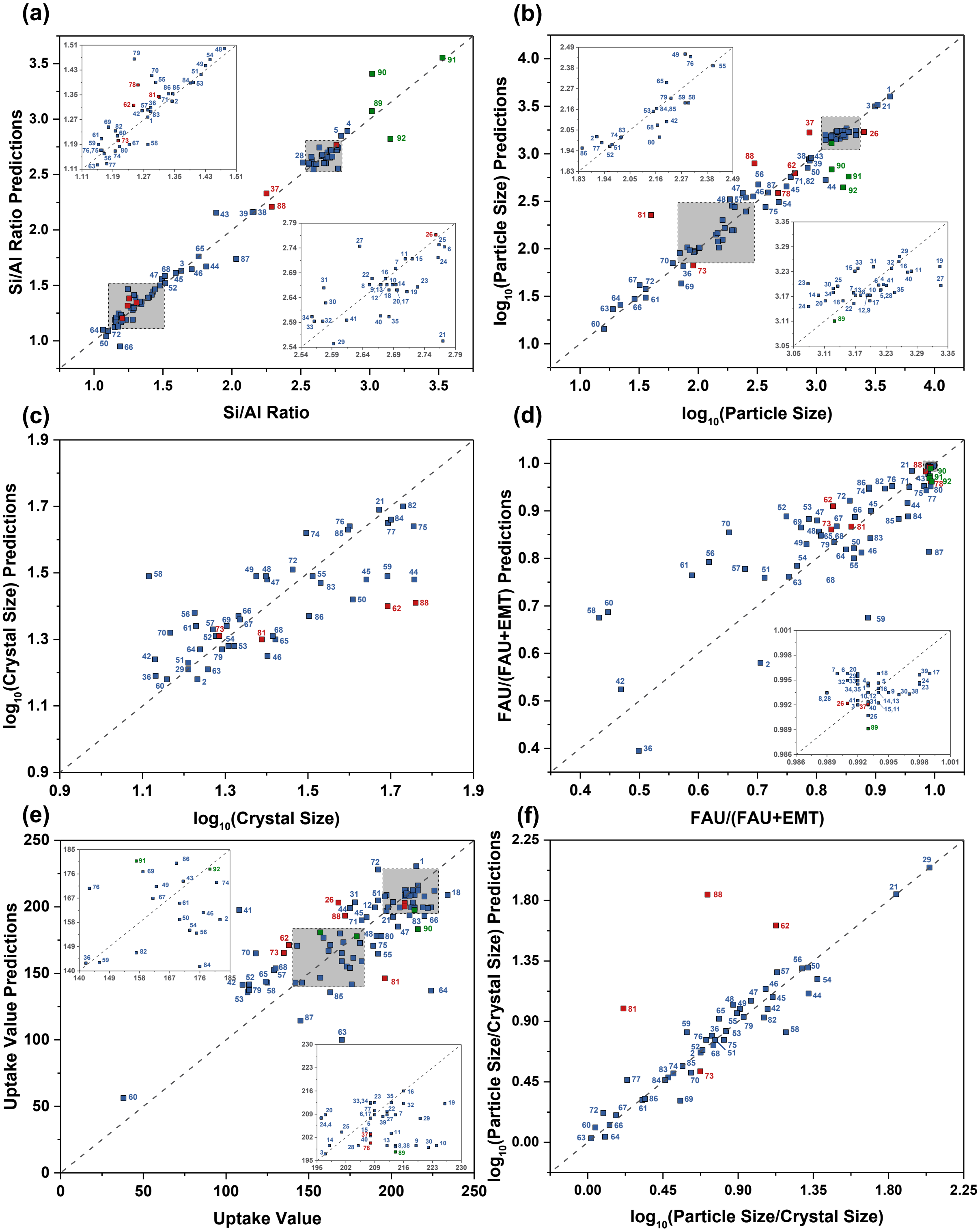

Supplementary Figure 28. Details of Fig. 2 in the main text regarding the correlation of experimental with predicted physical properties developed from the machine learning algorithm rescaled 10-CV. (a) Si/Al ratio, (b) $\log_{10}$(Particle Size), (c) $\log_{10}$(Crystal Size), (d) FAU/(FAU+EMT), (e) uptake value, (f) $\log_{10}$(Particle Size/Crystal Size). Blue dots represent training points to build up a model, red dots represent testing points to examine the model, and green dots represent prediction points to get high-silica FAU zeolites.

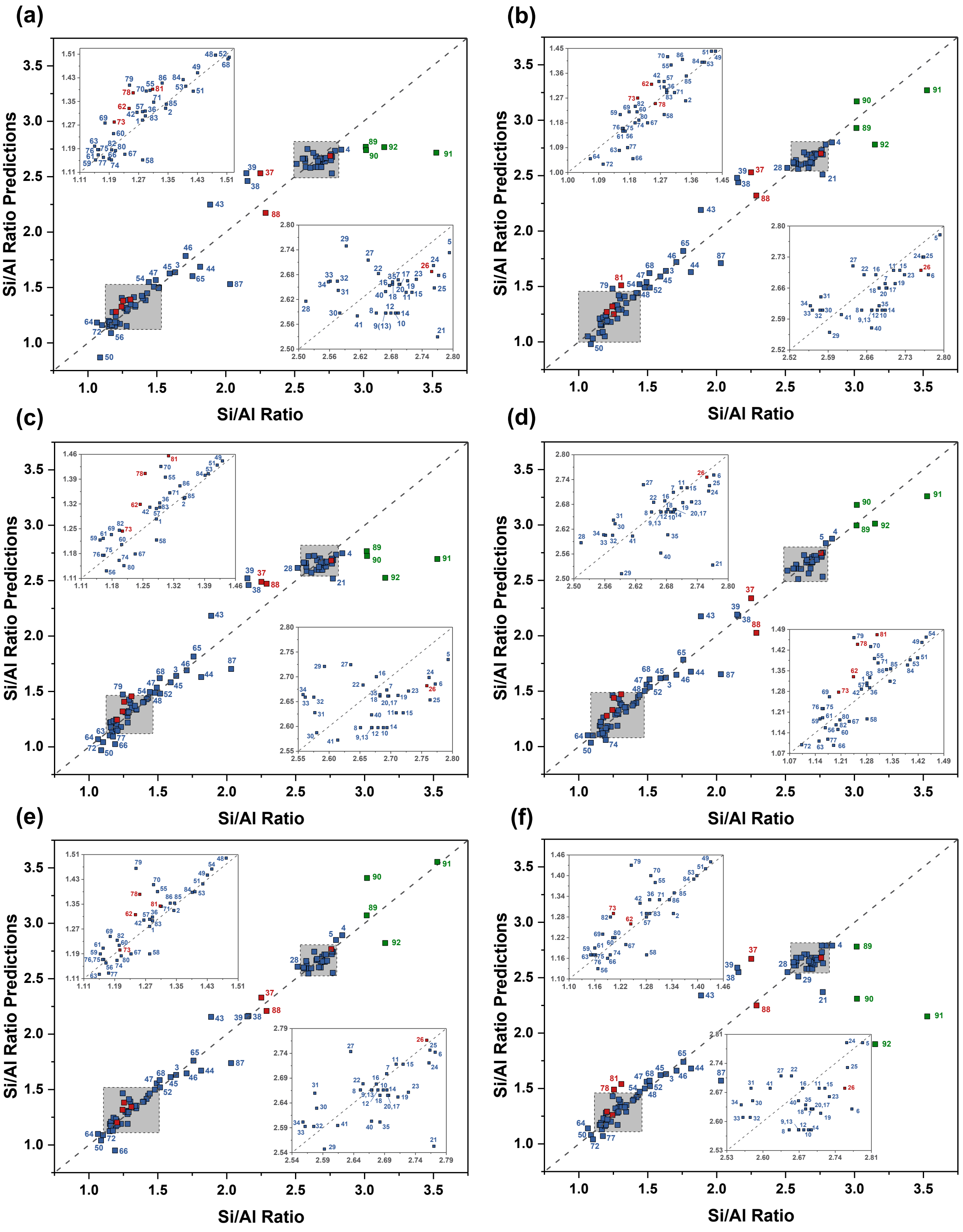

Supplementary Figure 29. Correlation of experimental Si/Al ratio with predicted Si/Al ratio developed from different machine learning algorithms. (a) Normalized 5-CV, (b) normalized 10-CV, (c) normalized LOOCV, (d) rescaled 5-CV, (e) rescaled 10-CV, (f) rescaled LOOCV. Blue dots represent training points to build up a model, red dots represent testing points to examine the model, and green dots represent prediction points to get high-silica FAU zeolites.

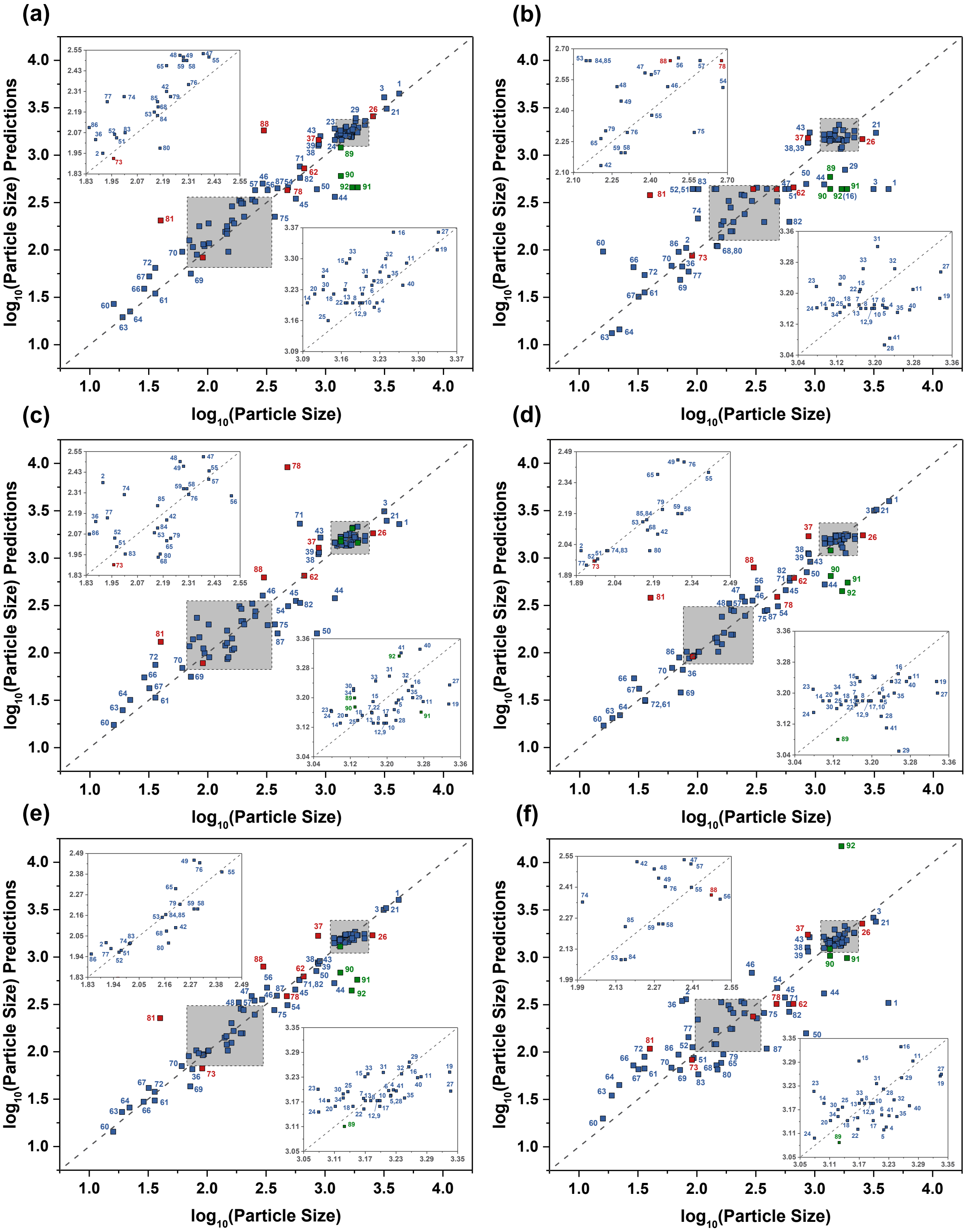

Supplementary Figure 30. Correlation of experimental log10(Particle Size) with predicted log10(Particle Size) developed from different machine learning algorithms. (a) Normalized 5-CV, (b) normalized 10-CV, (c) normalized LOOCV, (d) rescaled 5-CV, (e) rescaled 10-CV, (f) rescaled LOOCV. Blue dots represent training points to build up a model, red dots represent testing points to examine the model, and green dots represent prediction points to get high-silica FAU zeolites.

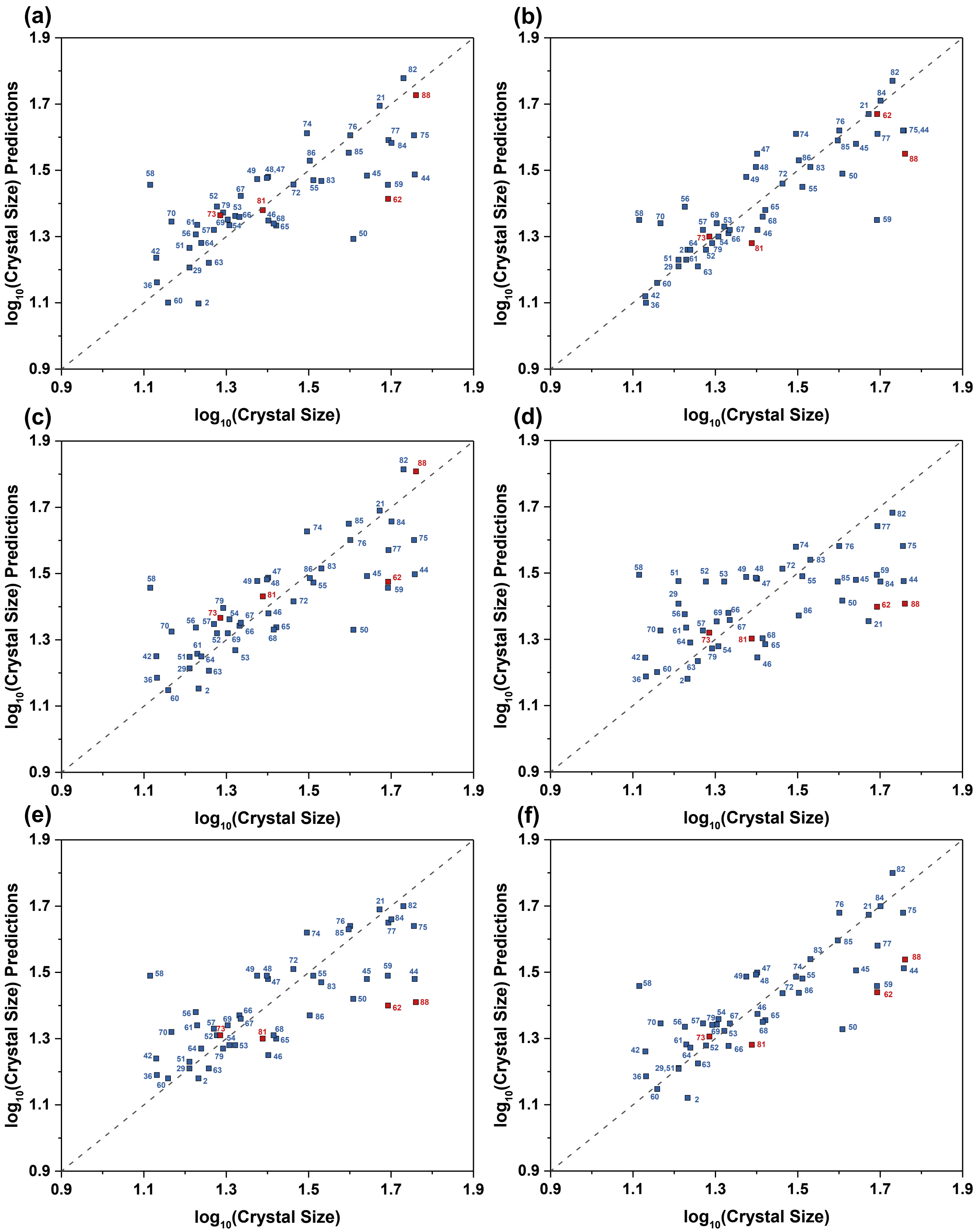

Supplementary Figure 31. Correlation of experimental log10(Crystal Size) with predicted log10(Crystal Size) developed from different machine learning algorithms. (a) Normalized 5-CV, (b) normalized 10-CV, (c) normalized LOOCV, (d) rescaled 5-CV, (e) rescaled 10-CV, (f) rescaled LOOCV. Blue dots represent training points to build up a model, red dots represent testing points to examine the model.

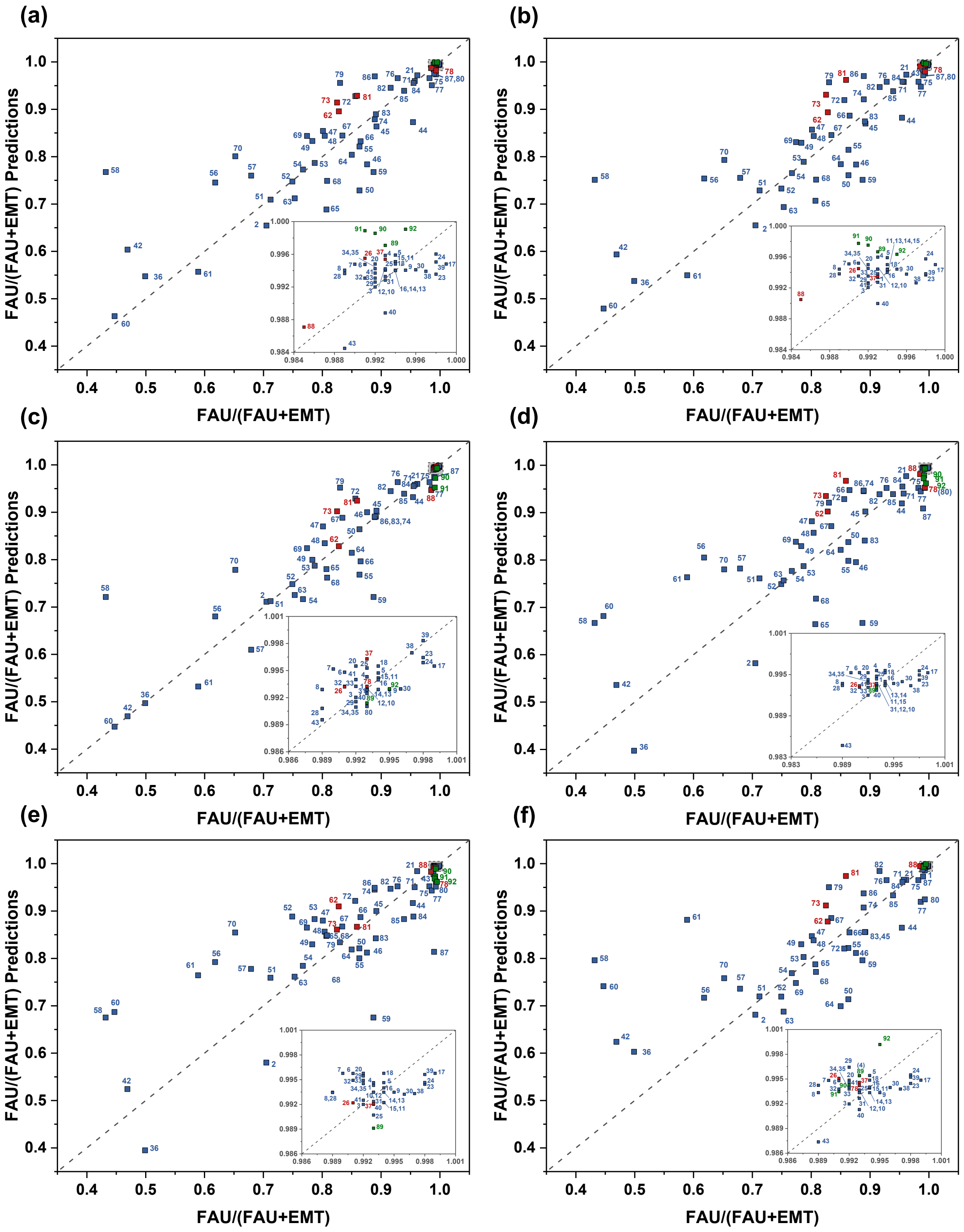

Supplementary Figure 32. Correlation of experimental FAU/(FAU+EMT) ratio with predicted FAU/(FAU+EMT) ratio developed from different machine learning algorithms. (a) Normalized 5-CV, (b) normalized 10-CV, (c) normalized LOOCV, (d) rescaled 5-CV, (e) rescaled 10-CV, (f) rescaled LOOCV. Blue dots represent training points to build up a model, red dots represent testing points to examine the model, and green dots represent prediction points to get high-silica FAU zeolites.

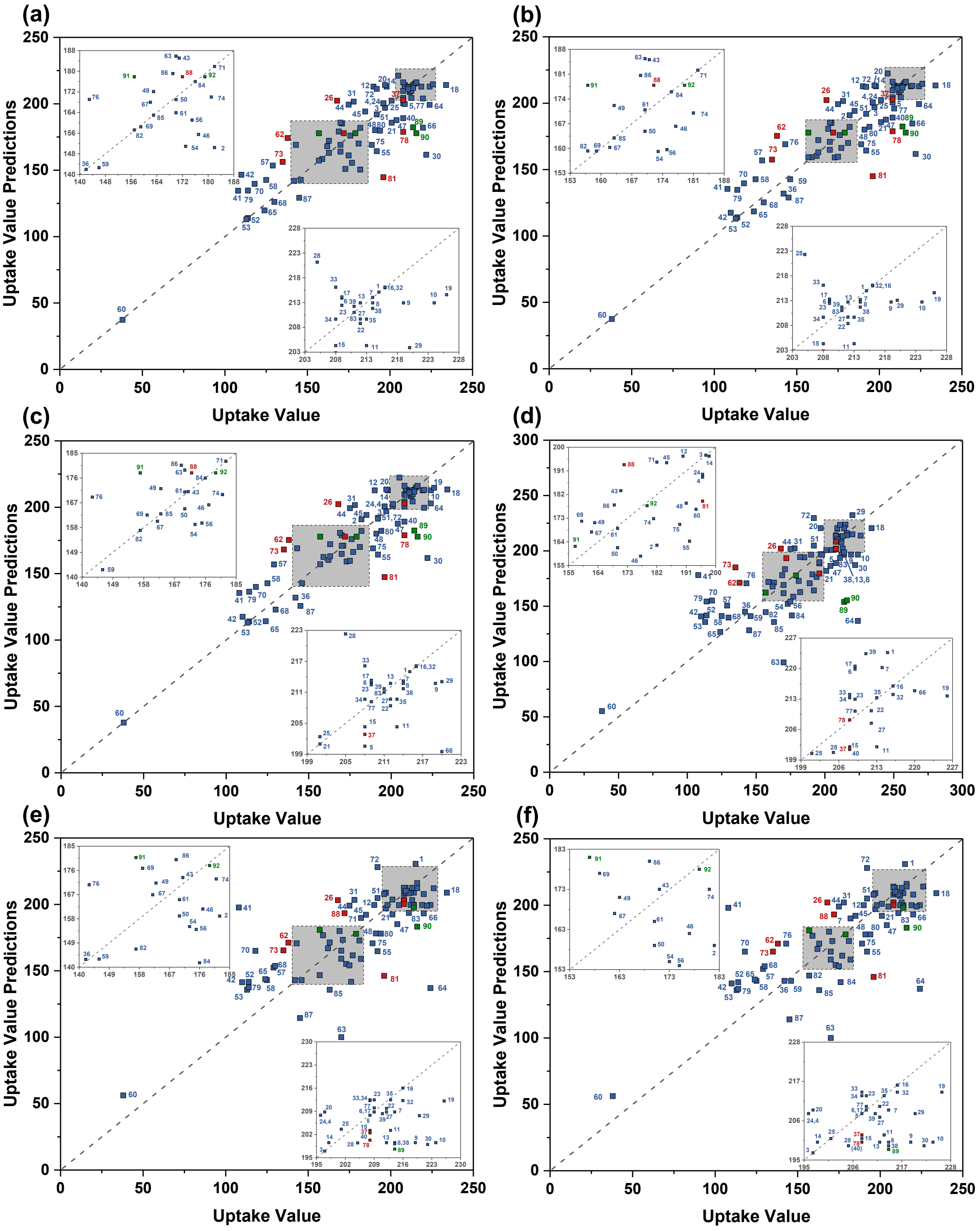

Supplementary Figure 33. Correlation of experimental uptake value (cm³/g) with predicted uptake value (cm³/g) developed from different machine learning algorithms. (a) Normalized 5-CV, (b) normalized 10-CV, (c) normalized LOOCV, (d) rescaled 5-CV, (e) rescaled 10-CV, (f) rescaled LOOCV. Blue dots represent training points to build up a model, red dots represent testing points to examine the model, and green dots represent prediction points to get high-silica FAU zeolites.

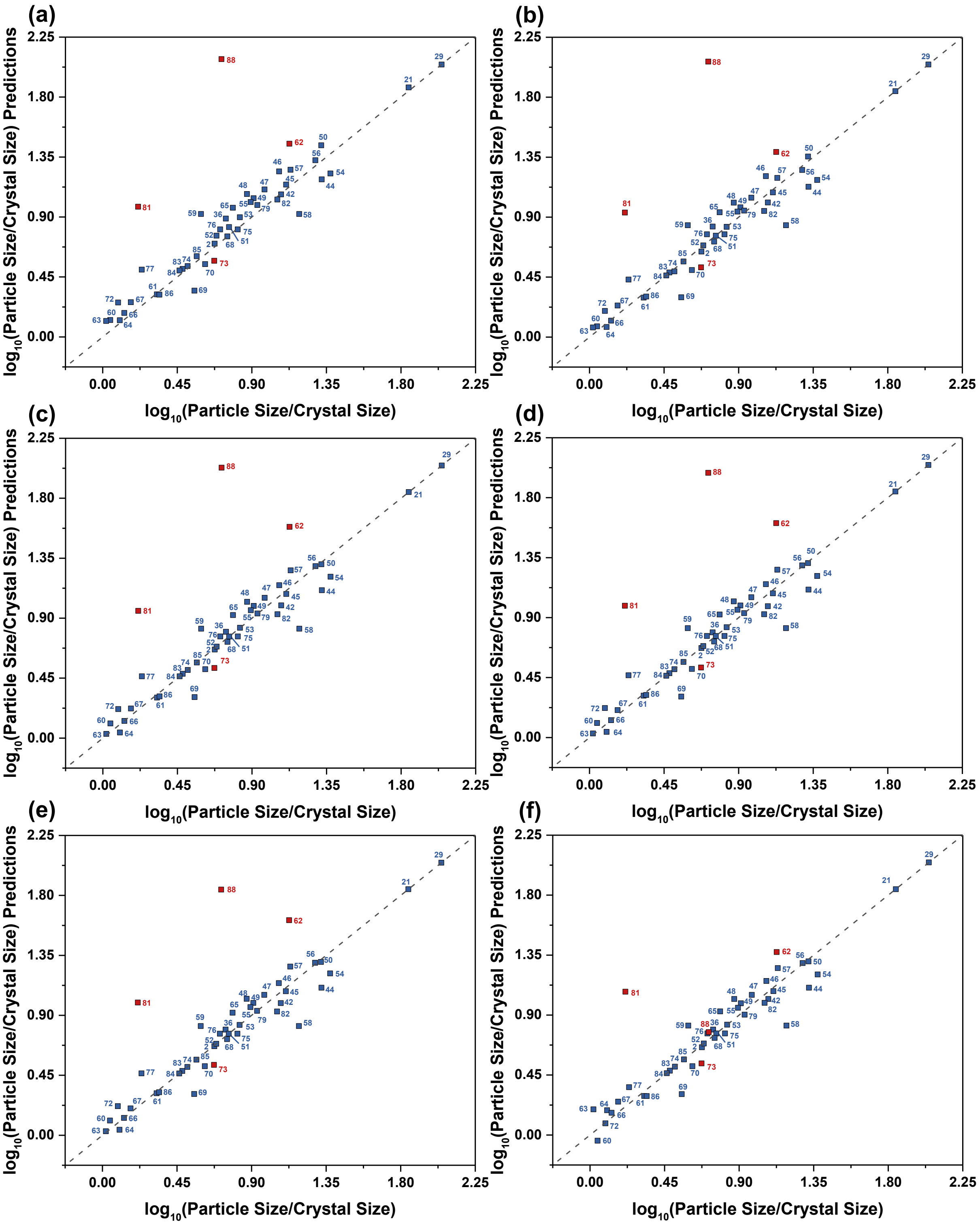

Supplementary Figure 34. Correlation of experimental $\log_{10}$(Particle Size/Crystal Size) with predicted $\log_{10}$(Particle Size/Crystal Size) developed from different machine learning algorithms. (a) Normalized 5-CV, (b) normalized 10-CV, (c) normalized LOOCV, (d) rescaled 5-CV, (e) rescaled 10-CV, (f) rescaled LOOCV. Blue dots represent training points to build up a model, red dots represent testing points to examine the model.

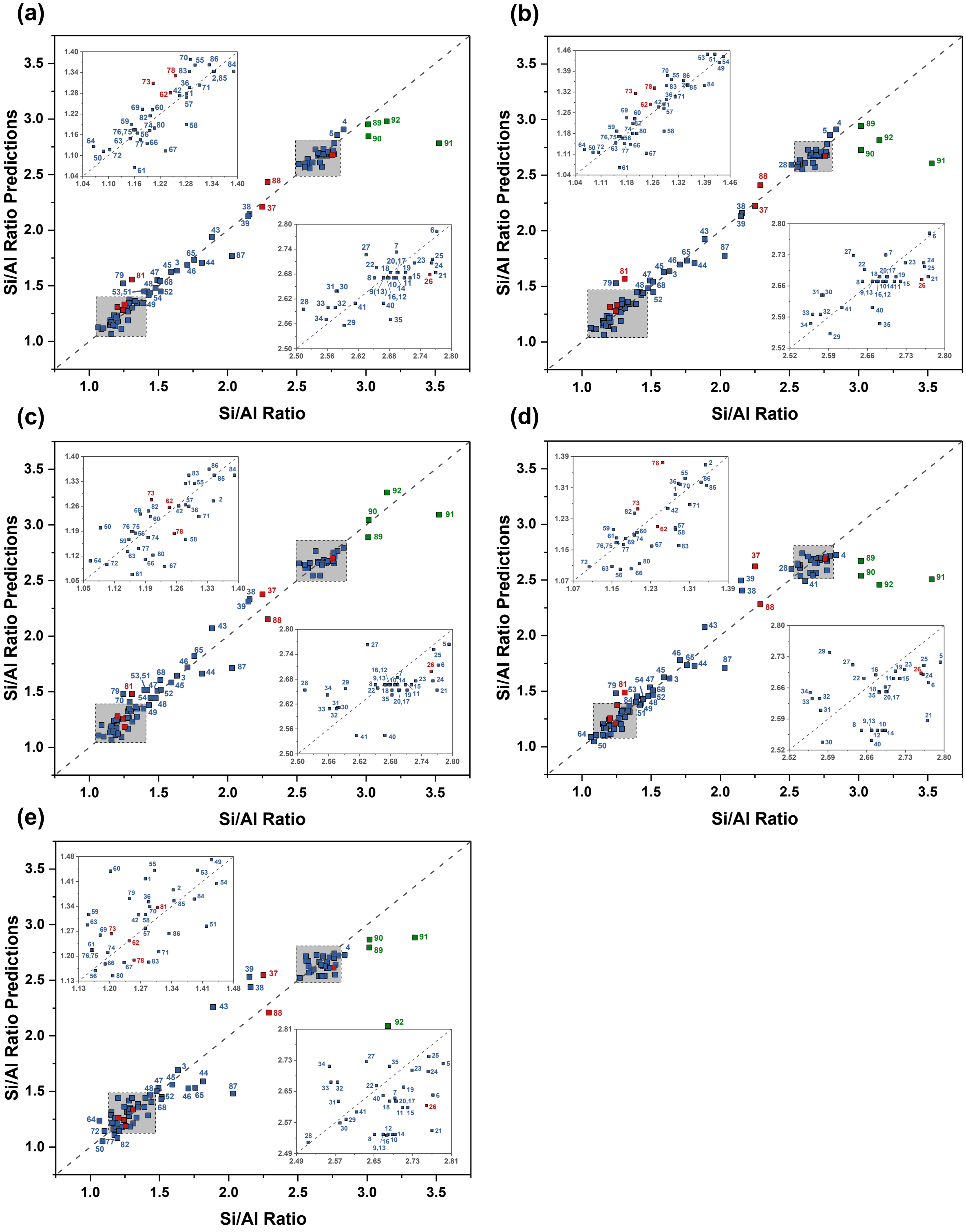

Supplementary Figure 35. Correlation of experimental Si/Al ratio with predicted Si/Al ratio developed from different machine learning algorithms. (a) Rescaled 5-CV (w/o categorical), (b) rescaled 10-CV (w/o categorical), (c) rescaled LOOCV (w/o categorical), (d) neural networks center, (e) neural networks rescaled. Blue dots represent training points to build up a model, red dots represent testing points to examine the model, and green dots represent prediction points to get high-silica FAU zeolites.

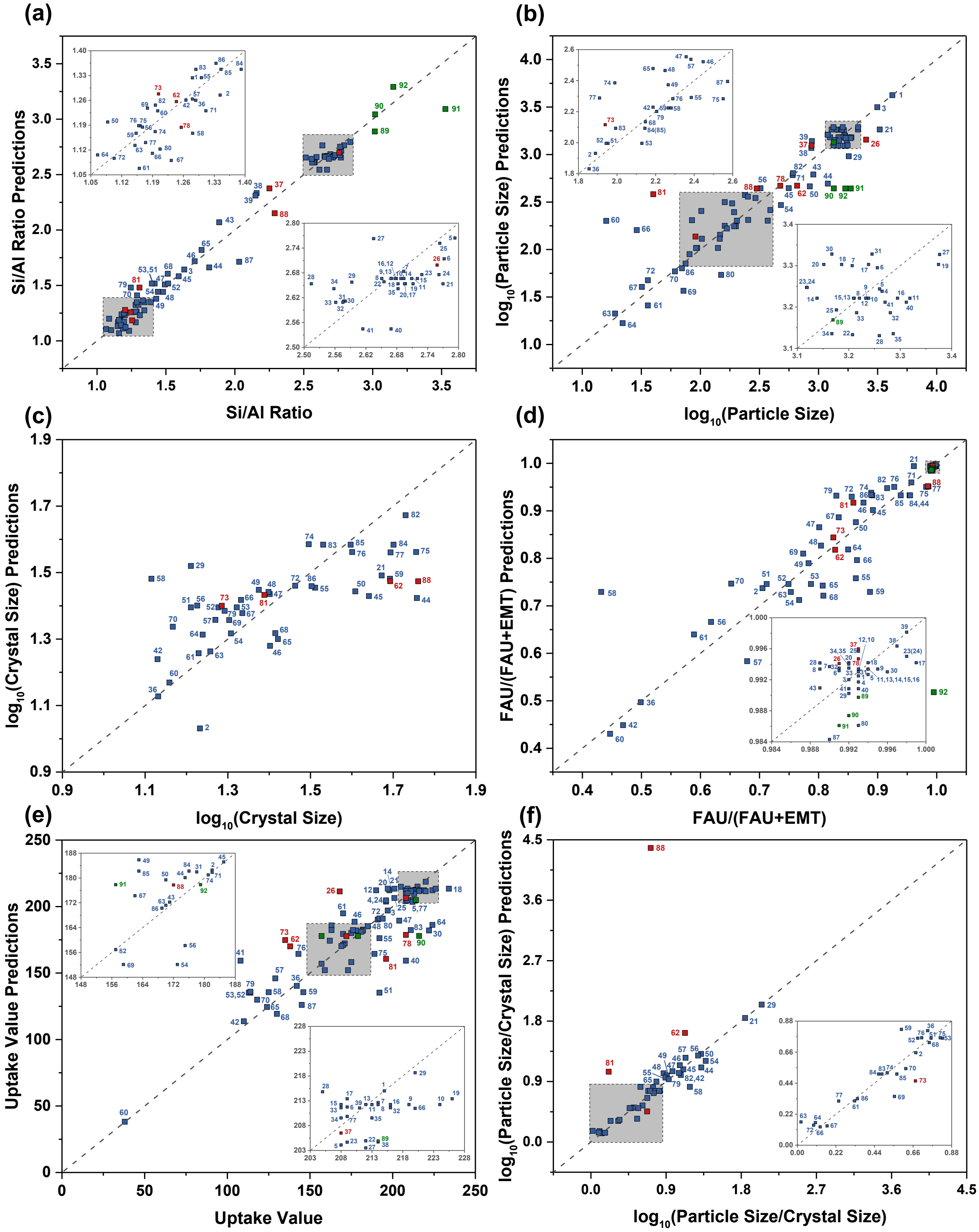

Supplementary Figure 36. Correlations of experimental with predicted physical properties developed from the machine learning algorithm "rescaled LOOCV (w/o categorical)". (a) Si/Al ratio, (b) $\log_{10}$(Particle Size), (c) $\log_{10}$(Crystal Size), (d) FAU/(FAU+EMT), (e) uptake value, (f) $\log_{10}$(Particle Size/Crystal Size). Blue dots represent training points to build up a model, red dots represent validation points to examine the model's predictive capability, and green dots represent prediction points to get high-silica FAU zeolites.

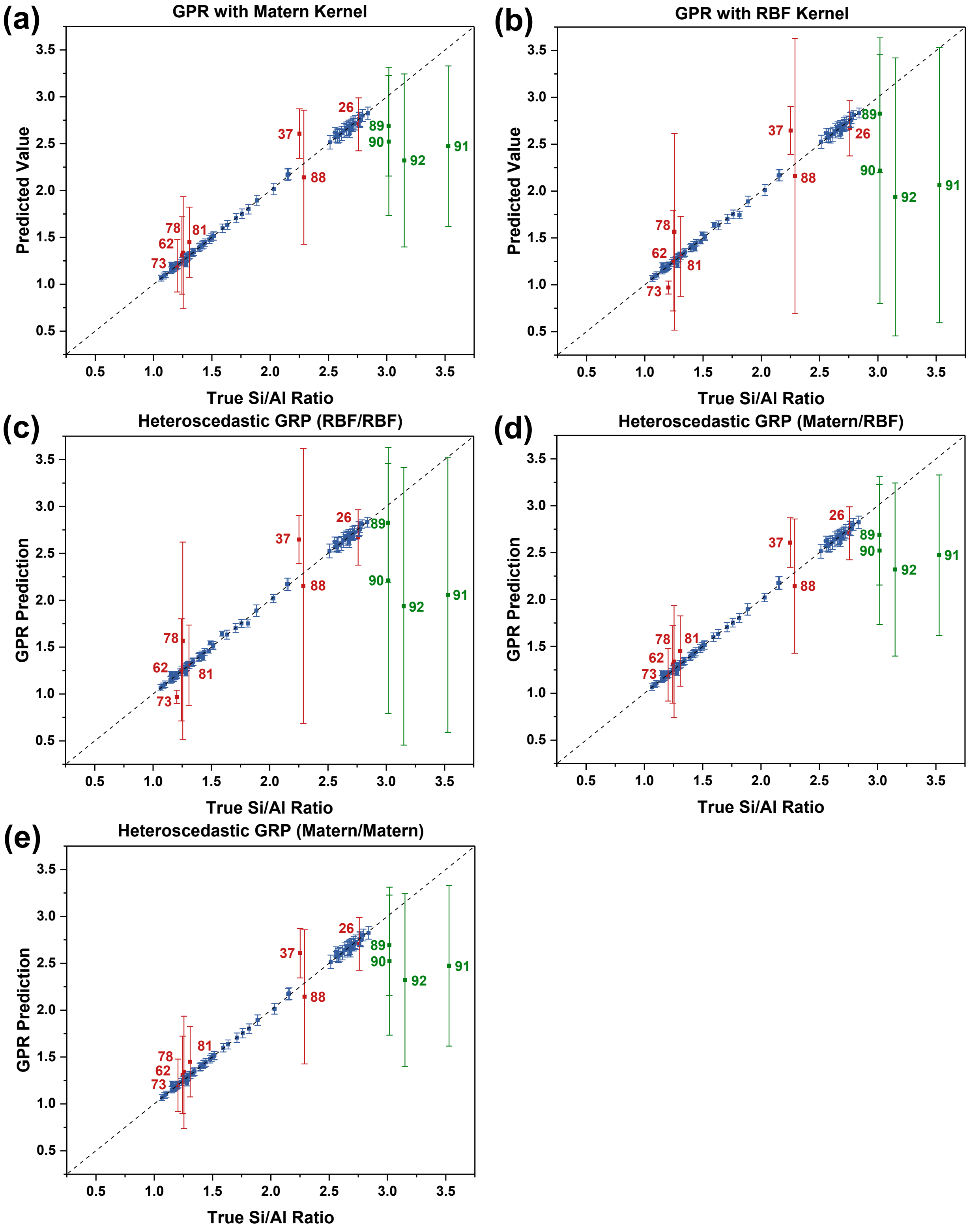

Supplementary Figure 37. Correlation for GPR predictions to the true Si/Al values for five variations of GPR model. We compare (a) a Matérn(0.5) kernel to (b) an RBF kernel, with "diagonal training noise" specified as 1.5% relative deviation. We also compare the fully heteroscedastic model with three combinations of mean kernel and uncertainty kernel, (c) RBF for mean, RBF for noise level, (d) Matérn(0.5) for mean, RBF for noise level, and (e) Matérn(0.5) for mean, Matérn(0.5) for noise level. A 1.5% relative deviation is applied as the uncertainty estimate for each training point. Blue entries represent training points, red entries represent testing points, and green entries represent prediction points. Training points (blue) are not labelled because they all fall very close to the x = y line. Here error bars represent 95% confidence intervals.

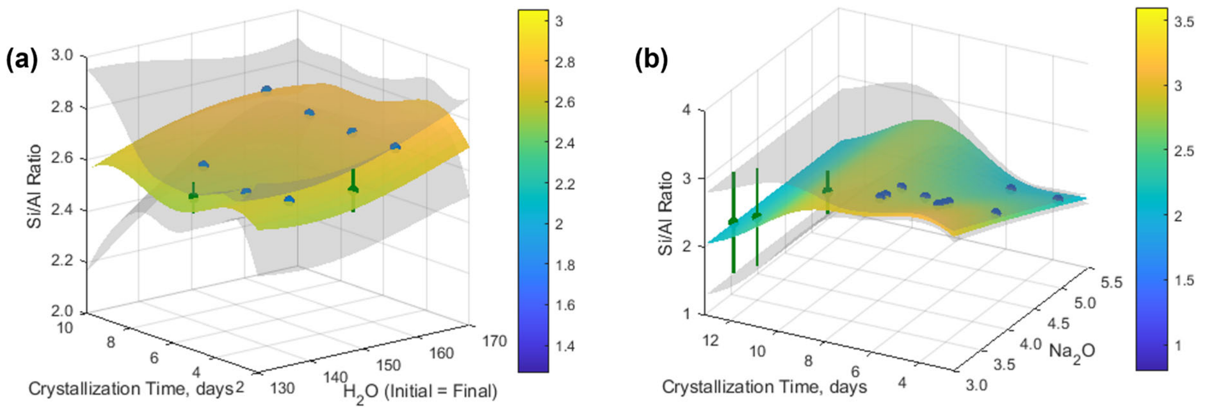

Supplementary Figure 38. Surface plots showing GPR's predicted mean (colored surfaces) and uncertainty (gray surfaces). In both plots (a)(b), the blue points were part of the training set, and the green points, with error bars, represent predictions by the trained model. The model is defined as a function of twelve variables, but here we illustrate two-dimensional slices through the domain for which all unseen coordinates are held constant.

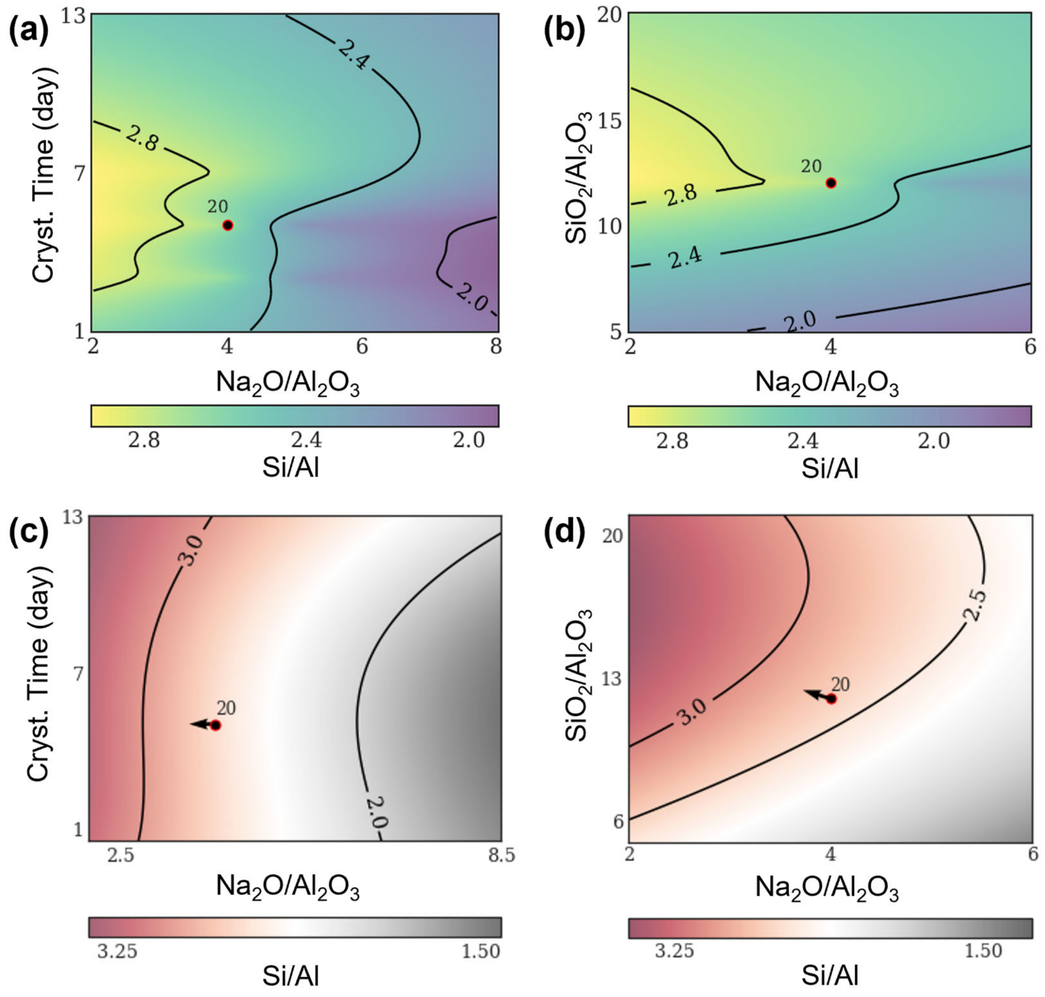

Supplementary Figure 39. Supplementary predictions for unseen experiments to exceed Si/Al = 3. Mean predictions via GPR with Matérn (0.5) kernel and uncertainty specified for each training point as 1.5% relative deviation, including (a) as crystallization time and Na_2O change, and (b) as SiO_2 and Na_2O change. (c) GH prediction ("rescaled LOOCV (w/o categorical)") as crystallization time and Na_2O change, (d) GH prediction ("rescaled LOOCV (w/o categorical)") as SiO_2 and Na_2O change.

S5. Input/Output Correlations from Raw Data and SHAP (Shapley Value Based) Analyses.

S5.1. Correlations Among Outputs

In order to evaluate the synthesis performance of faujasite zeolite, six outputs are selected to reflect the physical properties, including (1) Si/Al ratio; (2) FAU/(FAU+EMT); (3) Uptake Value; (4) Particle Size; (5) Crystal Size; (6) Particle Size / Crystal Size. If each two out of six outputs are correlated with each other, there exist $C_6^2 = 15$ combinations, corresponding 15 plots are listed as follows (Supplementary Figure 40). Several findings can be explored from Supplementary Figure 40:

(1) Once Si/Al ratio becomes higher than 2, FAU fraction is approaching unity, which means that intergrowth phenomenon is avoided and hence EMT formation is minimized during crystallization period (Supplementary Figure 40(a)).

(2) Generally, recipes leading to high silica content (Si/Al ratio) faujasite zeolites are accompanied with large particle sizes (Supplementary Figure 40(d)).

(3) Recipes leading to high purity (FAU fraction) faujasite zeolites are accompanied with large crystal sizes (Supplementary Figure 40(h)).

(4) Particle size can be positively correlated with crystal size (Supplementary Figure 40(j)). Subsequently, particle size can be positively correlated with the ratio of particle size / crystal size (Supplementary Figure 40(n)), and the latter is regarded as a descriptor of dispersion status.

(5) The ratio of particle size / crystal size (Particle / Crystal) can be positively correlated with Si/Al ratio (Supplementary Figure 40(k)).

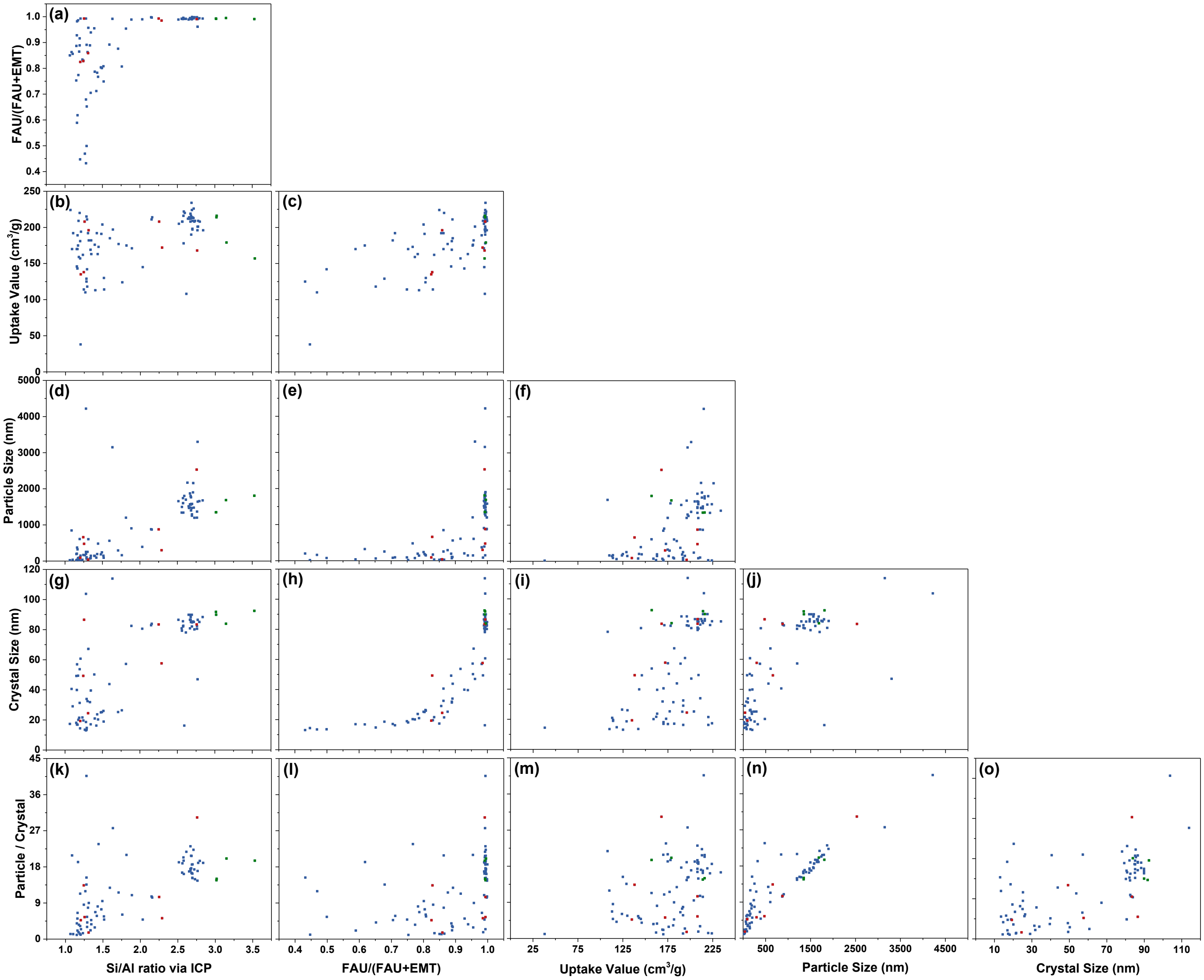

Supplementary Figure 40. Correlations among six selected outputs, including Si/Al ratio, FAU/(FAU+EMT), Uptake Value, Particle Size, Crystal Size, and Particle / Crystal. Note: only crystal sizes smaller than 60 nm are included to train the machine learning models. Blue/red/green dots represent training/testing/prediction points, respectively.

S5.2. Correlations Between Outputs and Inputs

As mentioned above, six outputs are selected to reflect the physical properties, including (1) Si/Al ratio; (2) FAU/(FAU+EMT); (3) Uptake Value; (4) Particle Size; (5) Crystal Size; (6) Particle / Crystal. Accordingly, seven inputs are selected from recipes to build up correlations with outputs respectively. Seven inputs based on recipes consist of (1) SiO_2/Al_2O_3; (2) Na_2O/SiO_2; (3) Na_2O/Al_2O_3; (4) Na_2O/H_2O_{final}; (5) $Na_2O/H_2O_{initial}$; (6) crystallization temperature; and (7) crystallization time. As shown in Supplementary Figure 41, reducing relative Na_2O amount (e.g., Na_2O/SiO_2, Na_2O/Al_2O_3, Na_2O/H_2O_{final}, $Na_2O/H_2O_{initial}$) will lead to the increase of Si/Al ratio, particle size, and the ratio of particle size / crystal size. Simultaneously, we deem that multiple factors contribute to affect

variations of crystal size, FAU fraction, and uptake value, due to the lack of monotonic trends for these outputs in Supplementary Figures 41 ~ 43.

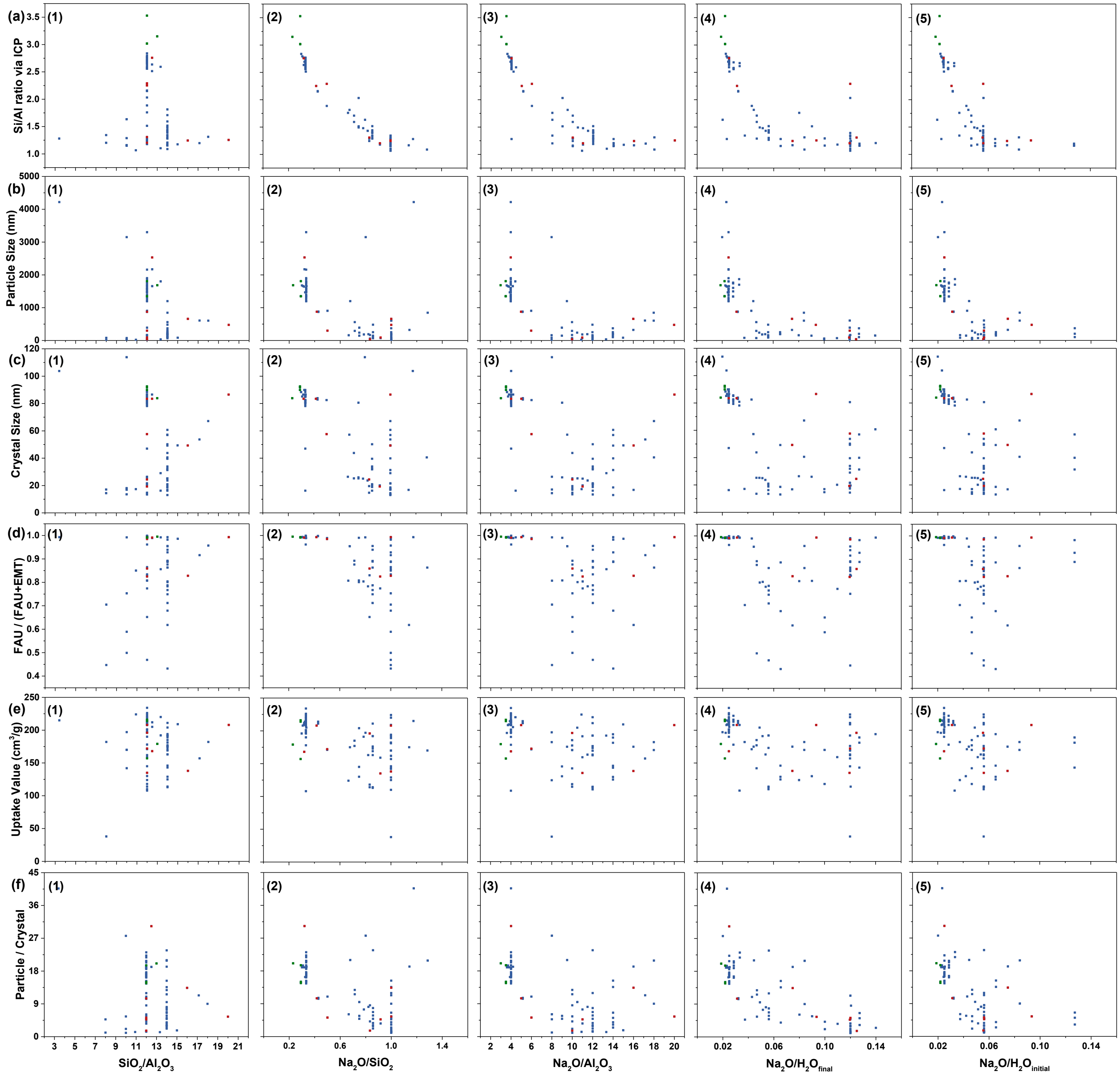

Supplementary Figure 41. Correlations between six selected outputs and five selected inputs based on synthesis compositions. Outputs consist of Si/Al ratio, FAU/(FAU+EMT), Uptake Value, Particle Size, Crystal Size, and Particle / Crystal. Inputs consist of SiO_2/Al_2O_3, Na_2O/SiO_2, Na_2O/Al_2O_3, Na_2O/H_2O_{final}, $Na_2O/H_2O_{initial}$. Note: only crystal sizes smaller than 60 nm are included to train the machine learning models. Blue/red/green dots represent training/testing/prediction points, respectively.

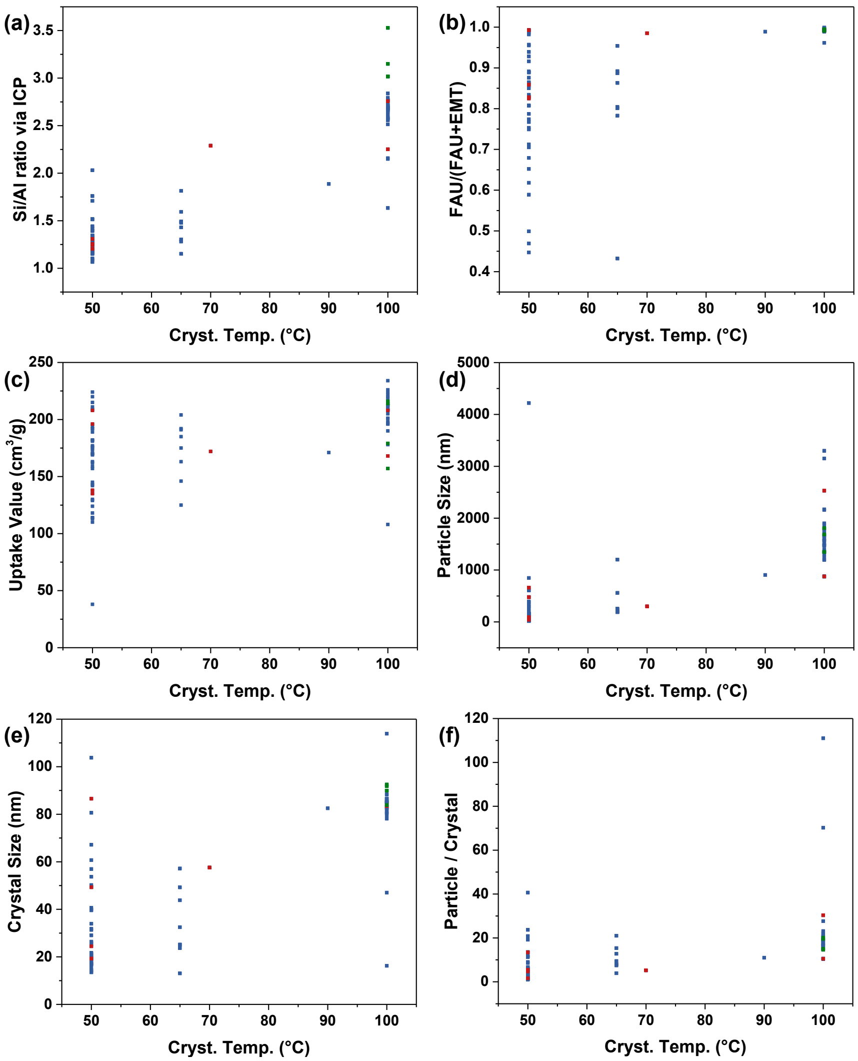

Supplementary Figure 42. Correlations between six selected outputs and the selected input crystallization temperature. Outputs consist of Si/Al ratio, FAU/(FAU+EMT), Uptake Value, Particle Size, Crystal Size, and Particle / Crystal. Note: only crystal sizes smaller than 60 nm are included to train the machine learning models. Blue/red/green dots represent training/testing/prediction points, respectively.

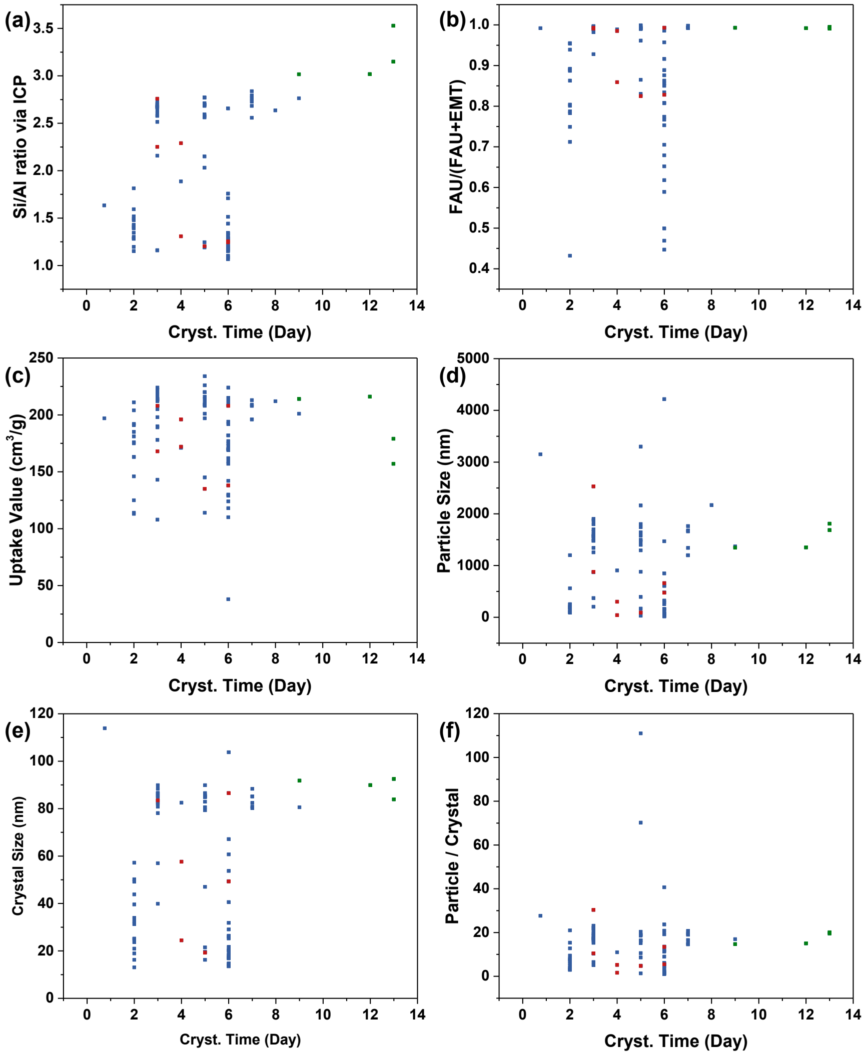

Supplementary Figure 43. Correlations between six selected outputs and the selected input crystallization time. Outputs consist of Si/Al ratio, FAU/(FAU+EMT), Uptake Value, Particle Size, Crystal Size, and Particle / Crystal. Note: only crystal sizes smaller than 60 nm are included to train the machine learning models. Blue/red/green dots represent training/testing/prediction points, respectively.

S5.3. SHAP Analysis

In this section, Supplementary Figure 44 shows the waterfall plots generated by SHAP analysis on the model "rescaled LOOCV (w/o categorical)" for the entries 89,90,92. Supplementary Figure 45 shows the corresponding plots for the model "rescaled 10-CV".

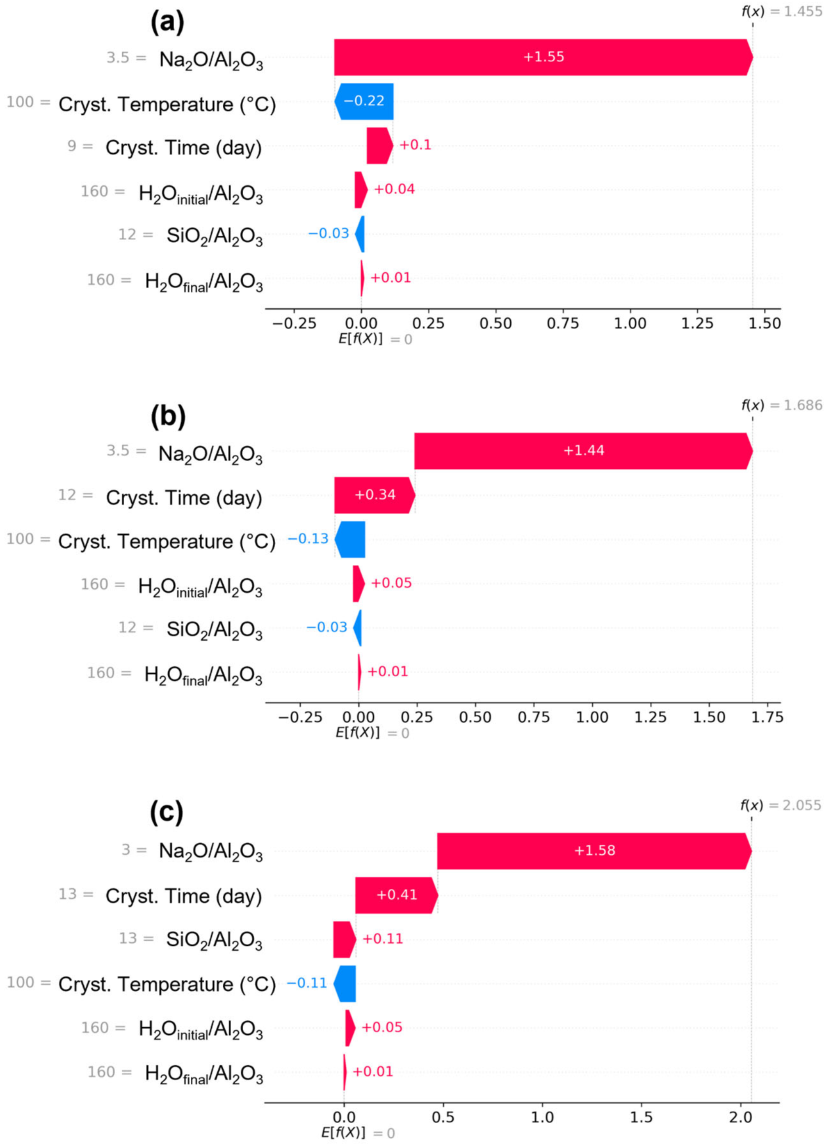

Supplementary Figure 44. The waterfall plots on the model "rescaled LOOCV (w/o categorical)" for entries (a) 89, (b) 90, and (c) 92 are shown respectively. The contribution of each input variable (synthesis condition) to the predicted normalized output $f(x)$ is shown. To compute the true value of the output, "denormalization" in the value of $f(x)$ needs to be applied: $f_{True}(x) = f(x)\sigma_{Si/Al} + \mu_{Si/Al}$, where $\sigma_{Si/Al}$ corresponds to the standard deviation and $\mu_{Si/Al}$ corresponds to the mean of the training set.

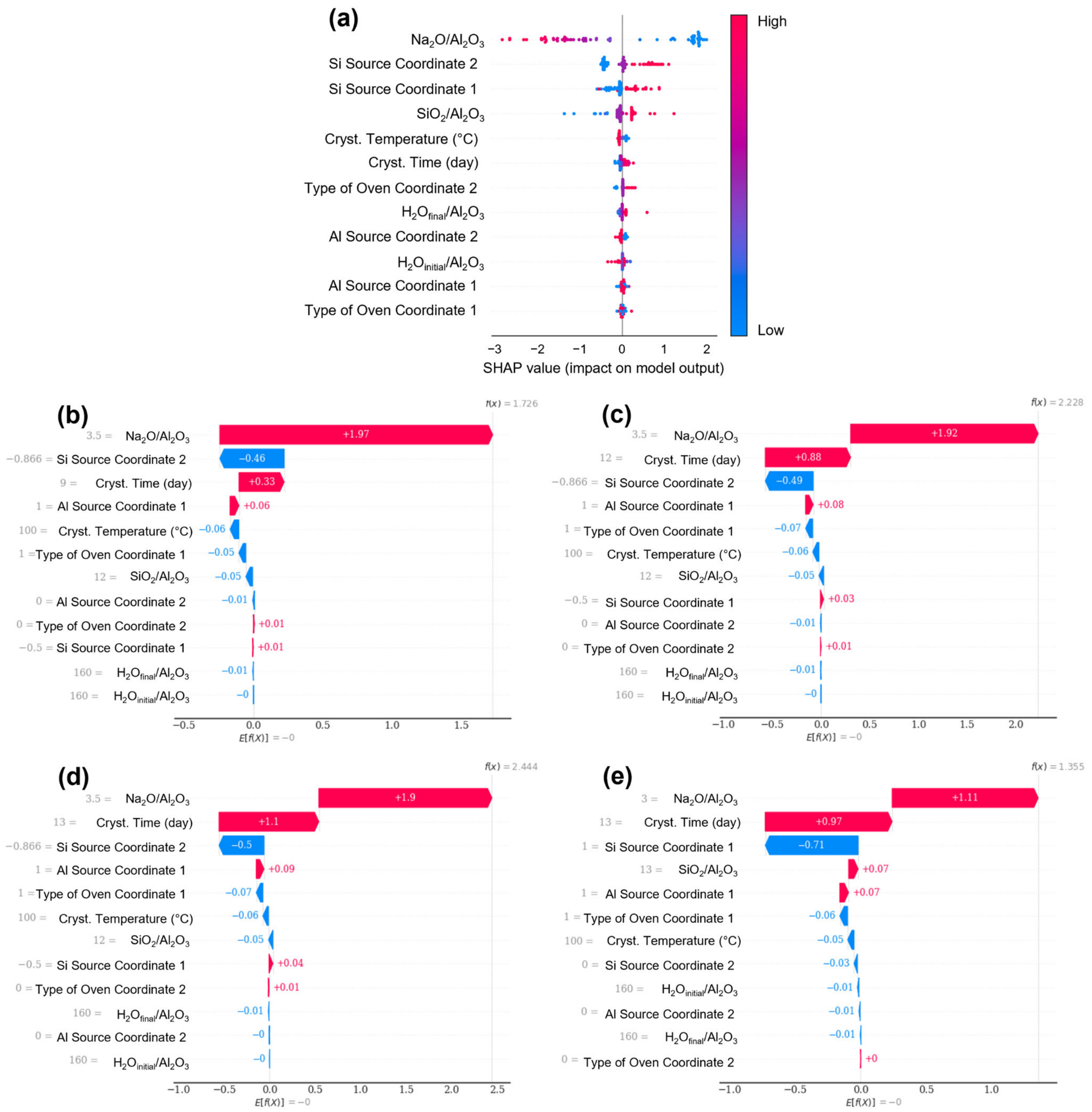

Supplementary Figure 45 The SHAP analyses for the trained Si/Al model "rescaled 10-CV". (a) The constructed SHAP summary plot. The waterfall plots for entries (b) 89, (c) 90, (d) 91, and (e) 92 are shown respectively. The contribution of each input variable (synthesis condition) to the predicted normalized output $f(x)$ is shown. To compute the true value of the output, "denormalization" in the value of $f(x)$ needs to be applied: $f_{True}(x) = f(x)\sigma_{Si/Al} + \mu_{Si/Al}$, where $\sigma_{Si/Al}$ corresponds to the standard deviation and $\mu_{Si/Al}$ corresponds to the mean of the training set.

S5.4. Quantitative Values of the Errors

For each of the two preprocessing schemes (normalizing and rescaling) and for each k-fold CV scheme (5-fold, 10-fold and LOOCV) we set a grid of values for the two hyperparameters (epsilon and number of eigenpairs) and we selected the optimum hyperparameters as follows.

Given a k-fold CV scheme, for each pair of hyperparameters we train (fit) the model for every split of that k-fold scheme, and compute the total error across the corresponding splits. For each k-fold scheme we pick the hyperparameter values that gave the smallest error.

Given these sets of optimum hyperparameters, we train once more the GH model using the entire training set. We then check the ability of the model to make predictions (generalize) on the test set and compute the MSE. We did not use the test points for the selection of hyperparameters or to optimize the test error. Our model "saw" the test points only after the k-fold CV scheme was performed, the optimum hyperparameters were selected, and a model was trained on those.

The procedure described above is repeated across the three k-fold CV schemes and the two preprocessing schemes leading to 6 models for each output, including (a) Normalized 5-CV, (b) normalized 10-CV, (c) normalized LOOCV, (d) rescaled 5-CV, (e) rescaled 10-CV, (f) rescaled LOOCV (Supplementary Figures 29~34). We then compare the MSE test error across those 6 different models (additionally including the model "rescaled LOOCV (w/o categorical)" for reference) to select the best overall model.

Supplementary Table 26. Quantitative values of the errors for each model

**Output**	**Model**	**R^2 Training**	**R^2 Test**	**MSE Training**	**MSE Test**	**Number of Eigenpairs ($\|S_\delta\|$)**	**Kernel bandwidth (ε)**
Si/Al	Normalized 5-CV	0.9697	0.9613	0.0136	0.0188	21	50.96
	Normalized 10-CV	0.9790	0.9674	0.0094	0.0190	28	2442.69
	Normalized LOOCV	0.9757	0.9743	0.0108	0.0211	26	50.33
	Rescaled 5-CV	0.9850	0.9568	0.0067	0.0215	27	12.90
	Rescaled 10-CV	0.9870	0.9907	0.0058	0.0050	28	12.90
	Rescaled LOOCV	0.9661	0.9251	0.0151	0.0428	29	0.13
	Rescaled 5-CV (w/o categorical)	0.9913	0.9768	0.0039	0.0154	25	0.80
	Rescaled 10-CV (w/o categorical)	0.9914	0.9777	0.0039	0.0157	27	0.80
	Rescaled LOOCV (w/o categorical)	0.9849	0.9713	0.0067	0.0112	20	0.80
	Normalized NN	0.9807	0.9474	0.0088	0.0274	N.A.	N.A.
	Rescaled NN	0.9774	0.9650	0.0109	0.0158	N.A.	N.A.

Output	Model	R^2 Training	R^2 Test	MSE Training	MSE Test	Number of Eigenpairs ($\lvert S_\delta \rvert$)	Kernel bandwidth (ε)
Si/Al GPR	Matern Diagonal 1.5%	0.9994	0.9401	0.0003	0.0260	N.A.	N.A.
	RBF Diagonal 1.5%	0.9990	0.8886	0.0004	0.0473	N.A.	N.A.
	Heteroscedastic Mat/Mat 1.5%	0.9994	0.9401	0.0003	0.0260	N.A.	N.A.
	Heteroscedastic Mat/RBF 1.5%	0.9994	0.9401	0.0003	0.0261	N.A.	N.A.
	Heteroscedastic RBF/RBF 1.5%	0.9990	0.8869	0.0004	0.0480	N.A.	N.A.

Output	Model	R^2 Training	R^2 Test	MSE Training	MSE Test	Number of Eigenpairs ($\lvert S_\delta \rvert$)	Kernel bandwidth (ε)
log(Particle Size)	Normalized 5-CV	0.9565	0.6604	0.0217	0.1667	28	50.96
	Normalized 10-CV	0.7937	0.5568	0.0804	0.1595	22	0.49
	Normalized LOOCV	0.9088	0.5257	0.0355	0.2949	22	50.96
	Rescaled 5-CV	0.9716	0.5649	0.0111	0.1790	37	0.13
	Rescaled 10-CV	0.9783	0.6722	0.0085	0.1258	40	0.13
	Rescaled LOOCV	0.8299	0.8140	0.0662	0.0592	20	625.93
	Rescaled LOOCV (w/o categorical)	0.8893	0.6200	0.0433	0.1605	23	0.0078

Output	Model	R^2 Training	R^2 Test	MSE Training	MSE Test	Number of Eigenpairs ($\lvert S_\delta \rvert$)	Kernel bandwidth (ε)
log(Crystal Size)	Normalized 5-CV	0.6114	0.5580	0.0144	0.0213	13	1273.78
	Normalized 10-CV	0.7564	0.8171	0.0090	0.0142	22	51.43
	Normalized LOOCV	0.6508	0.6426	0.0129	0.0145	15	407.95
	Rescaled 5-CV	0.4192	0.8981	0.0215	0.0550	8	0.13
	Rescaled 10-CV	0.6462	0.9344	0.0131	0.0542	13	0.13
	Rescaled LOOCV	0.6764	0.8846	0.0120	0.0314	20	13.33
	Rescaled LOOCV (w/o categorical)	0.4663	0.9477	0.0197	0.0363	6	0.97

Output	Model	R^2 Training	R^2 Test	MSE Training	MSE Test	Number of Eigenpairs ($\lvert S_\delta \rvert$)	Kernel bandwidth (ε)
FAU /(FAU+EMT)	Normalized 5-CV	0.8183	0.9661	0.0038	0.0025	33	51.52
	Normalized 10-CV	0.8307	0.7973	0.0036	0.0038	31	51.52
	Normalized LOOCV	0.8797	0.7740	0.0025	0.0017	40	1837.10
	Rescaled 5-CV	0.7672	0.6024	0.0049	0.0044	26	0.11
	Rescaled 10-CV	0.7443	0.8614	0.0055	0.0013	21	0.13
	Rescaled LOOCV	0.7122	0.7656	0.0062	0.0033	26	51.52
	Rescaled LOOCV (w/o categorical)	0.8714	0.8872	0.0027	0.0007	26	51.52

**Output**	**Model**	R^2 **Training**	R^2 **Test**	**MSE Training**	**MSE Test**	**Number of Eigenpairs** ($\lvert S_\delta \rvert$)	**Kernel bandwidth** (ε)
log(Particle to Crystal Size)	Normalized 5-CV	0.9393	0.1191	0.0151	0.6423	23	51.43
	Normalized 10-CV	0.9414	0.1087	0.0121	0.6088	23	51.43
	Normalized LOOCV	0.9398	0.1850	0.0124	0.6261	25	713.54
	Rescaled 5-CV	0.9395	0.1861	0.0124	0.6171	25	13.33
	Rescaled 10-CV	0.9399	0.2111	0.0124	0.5355	25	647.04
	Rescaled LOOCV	0.9421	0.0967	0.0119	0.2063	25	0.13
	Rescaled LOOCV (w/o categorical)	0.9478	0.0373	0.0107	3.599	25	0.13
**Output**	**Model**	R^2 **Training**	R^2 **Test**	**MSE Training**	**MSE Test**	**Number of Eigenpairs** ($\lvert S_\delta \rvert$)	**Kernel bandwidth** (ε)
log(Uptake Ratio)	Normalized 5-CV	0.8729	0.0574	0.0016	0.0061	34	0.49
	Normalized 10-CV	0.8957	0.0484	0.0014	0.0062	38	0.49
	Normalized LOOCV	0.9025	0.0164	0.0013	0.0066	40	0.49
	Rescaled 5-CV	0.6815	0.4016	0.0041	0.0054	24	0.13
	Rescaled 10-CV	0.6538	0.1025	0.0045	0.0061	20	0.13
	Rescaled LOOCV	0.6539	0.1025	0.0045	0.0060	21	0.13
	Rescaled LOOCV (w/o categorical)	0.8563	0.0539	0.0018	0.0061	37	0.0078

Note: We omit the hyperparameters ($\lvert S_\delta \rvert$) and ε) for the models trained with GPR and NN since cross-validation was not used for them.

S6. Supporting Information for Framework Si/Al ratio, Infrared Spectra and Reactivity of Faujasite Zeolites

The framework Si/Al ratios (Table 1, columns 4&5, Supplementary Table 27) could be estimated from the ^{29}Si-NMR spectra (Fig. 4(f)) based on "Loewenstein's rule" (the equation below),[13] which stipulates the absence of Al-O-Al linkages in the zeolite framework.

$$\frac{\text{Si}}{\text{Al}} = \sum_{x=0}^{4} I_{\text{Si(OAl)}_x} \bigg/ 0.25 \sum_{x=0}^{4} x I_{\text{Si(OAl)}_x} \qquad (15)$$

These framework Si/Al ratios (Table 1, columns 4&5) over H-FAU materials can be combined with Na/Al ratios via ICP analysis (Table 1, column 3) to calculate chemical formulae (Table 1, column 6), and determine H^+ site densities over these two H-FAU materials.

Supplementary Table 27. Distribution of Q^4(nAl) Si species on Na-FAU and H-FAU zeolites.

Zeolite[a]	Percentage of Q^4(nAl) species				Si/Al
	3Al	2Al	1Al	0Al	
Na-FAU3.5	3.7	25.6	52.5	18.2	3.48
H-FAU3.5	4.6	21.1	54.2	20.0	3.63
Na-FAU2.8	7.5	37.6	48.5	6.3	2.73
H-FAU2.8	8.3	38.6	41.3	11.9	2.79

Note: [a]. 2.8 and 3.5 were measured from ICP analysis for corresponding Na-FAU zeolites. Na-FAU3.5 was prepared from entry 91 of Supplementary Table 1, and Na-FAU2.8 was prepared from entry 24 of Supplementary Table 1.

Samples of Entry 86 and Entry 91 were independently tested to measure Si/Al ratio via ICP analysis for 5 times. Supplementary Table 28 shows the reproducibility for results of ICP analysis, which also provides a 1.5% relative deviation used to estimate uncertainty of the Machine Learning algorithms.

Supplementary Table 28. Distribution of Q^4(nAl) Si species on Na-FAU and H-FAU zeolites.

Zeolite	Si/Al ratio		Average	Standard Deviation	Ratio of Standard Deviation / Average
	test 1	1.318			
	test 2	1.311			
Entry 86	test 3	1.308	1.310	0.006	0.42 %
	test 4	1.303			
	test 5	1.310			
	test 1	3.474			
	test 2	3.592			
Entry 91	test 3	3.515	3.528	0.043	1.21 %
	test 4	3.524			
	test 5	3.535			

Supplementary Figure 46 reflects the fact that protons could be introduced into both supercages (ascribed to the band at ~ 3640 cm^{-1}) and solidate cages (ascribed to the band at ~ 3550 cm^{-1}) over both H-FAU3.5 and H-FAU2.8 zeolites. Subtraction infrared spectra between before (thick lines) and after (thin lines) pyridine adsorption (Supplementary Figure 46(c)) reflect that pyridine molecules predominantly titrate protons within supercages while protons within sodalite cages are rarely titrated. Since protons within solidate cages are able to be fully titrated only when significant framework collapse take place (e.g., harsh steaming process to prepare ultra-stable Y),[14] both H-FAU3.5 and H-FAU2.8 zeolites are deemed to still sustain framework stable after ion exchange with 1 M of NH_4NO_3 solution.

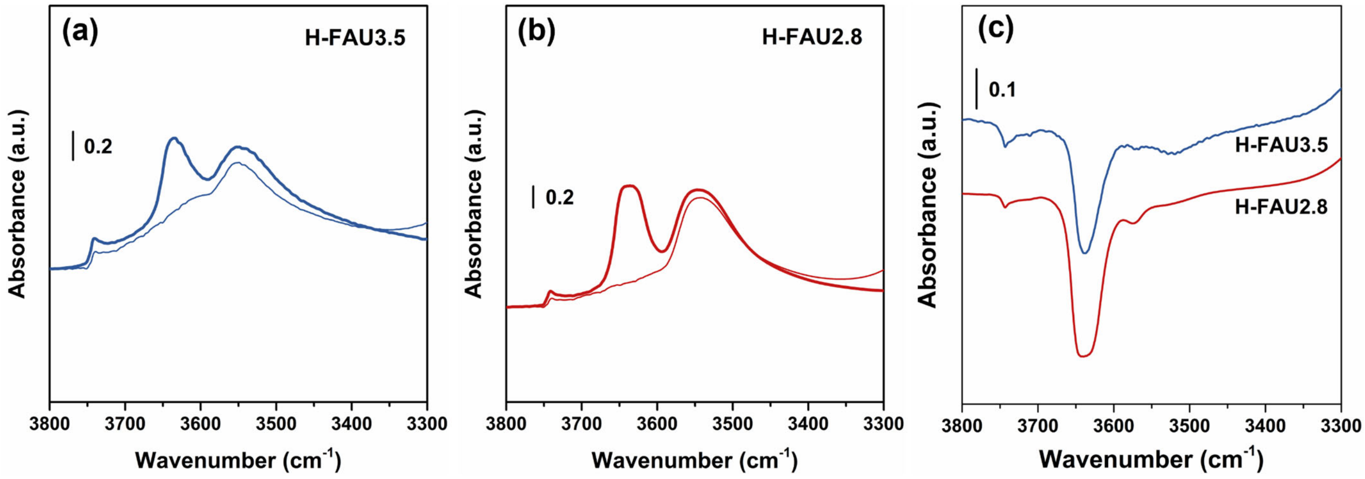

Supplementary Figure 46. Infrared spectra for before (thick lines) and after (thin lines) pyridine adsorption until saturation over dehydrated (a) H-FAU3.5 and (b) H-FAU2.8 zeolites at 498 K. (c) Subtraction infrared spectra between before and after pyridine adsorption until saturation over dehydrated H-FAU zeolites at 498 K.

Supplementary Figure 47 exhibits propane dehydrogenation and cracking rate constants per gram for H-FAU3.5 and H-FAU2.8 zeolites. By comparison, H-FAU3.5 exhibits higher propane dehydrogenation and cracking rate constants per overall H^+ site than H-FAU2.8 (Fig. 4). Despite the lower proton density (Table 1, column 7), H-FAU3.5 also exhibits higher rate constants per gram (Supplementary Figure 47).

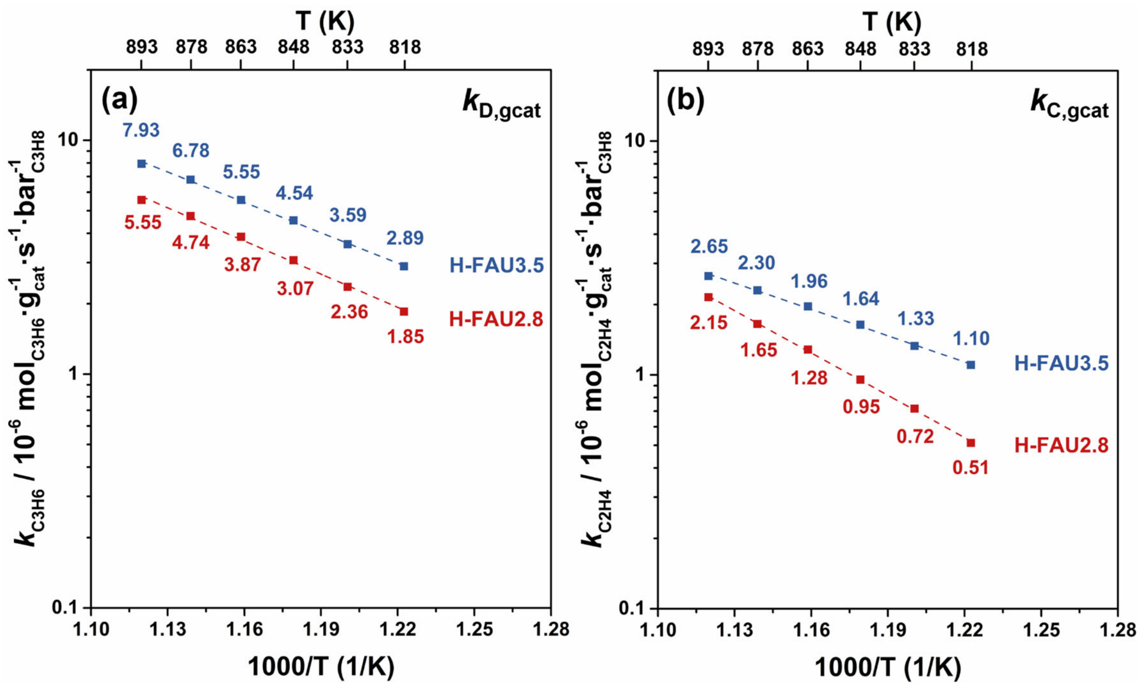

Supplementary Figure 47. Temperature dependence of rate constants on a per gram basis for (a) dehydrogenation and (b) cracking over H-FAU3.5 and H-FAU2.8 zeolites. Reaction conditions: $H_2/C_3H_8/Ar/He$ = 3/3/1.5/60, with a total pressure of 120 kPa and a total flow rate of 67.5 sccm, and space velocity = 3600 $cm^3_{C3H8}\cdot g_{cat}^{-1}\cdot h^{-1}$, time-on-stream = 60 seconds. Propane conversions are <1%.

Supplementary References

(1) Lafon, S. S. *Diffusion maps and geometric harmonics*; Yale University (2004).

(2) Coifman, R. R. & Lafon, S. Geometric harmonics: a novel tool for multiscale out-of-sample extension of empirical functions. *Appl. Comput. Harmon. Anal.* **21**, 31-52 (2006).

(3) Nyström, E. J. *Über die praktische auflösung von linearen integralgleichungen mit anwendungen auf randwertaufgaben der potentialtheorie*; Akademische Buchhandlung (1929).

(4) Fowlkes, C.; Belongie, S. & Malik, J. Proceedings of the 2001 IEEE Computer Society Conference on Computer Vision and Pattern Recognition. CVPR 2001, p I-I (2001).

(5) Gurney, K. *An introduction to neural networks*; CRC press (2018).

(6) Goodfellow, I.; Bengio, Y. & Courville, A. *Deep learning*; MIT press (2016).

(7) Rasmussen, C. E. & Williams, C. *Gaussian processes for machine learning, vol. 2, no. 3*; Cambridge, MA, USA: MIT Press (2006).

(8) Boyle, P. Gaussian processes for regression and optimisation. (2007).

(9) Goldberg, P.; Williams, C. & Bishop, C. Regression with input-dependent noise: A Gaussian process treatment. *Adv. Neural Inf. Process. Syst.* **10**, 493-499 (1997).

(10) Kersting, K.; Plagemann, C.; Pfaff, P. & Burgard, W. Proceedings of the 24th international conference on Machine learning, p 393-400 (2007).

(11) Wang, A. Y.-T. et al. Machine Learning for Materials Scientists: An Introductory Guide toward Best Practices. *Chem. Mater.* **32**, 4954-4965 (2020).

(12) James, G.; Witten, D.; Hastie, T. & Tibshirani, R. *An introduction to statistical learning*; Springer (2013).

(13) Fyfe, C. A. et al. One- and two-dimensional high-resolution solid-state NMR studies of zeolite lattice structures. *Chem. Rev.* **91**, 1525-1543 (1991).

(14) Batool, S. R.; Sushkevich, V. L. & van Bokhoven, J. A. Correlating Lewis acid activity to extra-framework aluminum species in zeolite Y introduced by Ion-exchange. *J. Catal.* **408**, 24-35 (2022).
