# Supplementary material for: Machine learning-assisted crystal engineering of a zeolite
Source: Nat Commun. 2023 May 31;14:3152. doi: 10.1038/s41467-023-38738-5 (PMC10232492; doi:10.1038/s41467-023-38738-5)
Supplement: Supplementary file 2 — Reporting Summary [file 41467_2023_38738_MOESM2_ESM.pdf]

nature portfolio

Corresponding author(s): Michael Tsapatsis

Last updated by author(s): Mar 27, 2023

Reporting Summary

Nature Portfolio wishes to improve the reproducibility of the work that we publish. This form provides structure for consistency and transparency in reporting. For further information on Nature Portfolio policies, see our Editorial Policies and the Editorial Policy Checklist.

Statistics

For all statistical analyses, confirm that the following items are present in the figure legend, table legend, main text, or Methods section.

n/a	Confirmed	
☐	☒	The exact sample size (n) for each experimental group/condition, given as a discrete number and unit of measurement
☐	☒	A statement on whether measurements were taken from distinct samples or whether the same sample was measured repeatedly
☐	☒	The statistical test(s) used AND whether they are one- or two-sided<br>*Only common tests should be described solely by name; describe more complex techniques in the Methods section.*
☐	☒	A description of all covariates tested
☐	☒	A description of any assumptions or corrections, such as tests of normality and adjustment for multiple comparisons
☐	☒	A full description of the statistical parameters including central tendency (e.g. means) or other basic estimates (e.g. regression coefficient) AND variation (e.g. standard deviation) or associated estimates of uncertainty (e.g. confidence intervals)
☐	☒	For null hypothesis testing, the test statistic (e.g. F, t, r) with confidence intervals, effect sizes, degrees of freedom and P value noted<br>*Give P values as exact values whenever suitable.*
☐	☒	For Bayesian analysis, information on the choice of priors and Markov chain Monte Carlo settings
☐	☒	For hierarchical and complex designs, identification of the appropriate level for tests and full reporting of outcomes
☐	☒	Estimates of effect sizes (e.g. Cohen's d, Pearson's r), indicating how they were calculated

Our web collection on statistics for biologists contains articles on many of the points above.

Software and code

Policy information about availability of computer code

Data collection	The codes used to train the Machine learning models can be accessed in the public Gitlab repository. (https://gitlab.com/nicolasevangelou/zeolites_ml.git)
Data analysis	The codes used to train the Machine learning models can be accessed in the public Gitlab repository. (https://gitlab.com/nicolasevangelou/zeolites_ml.git)

For manuscripts utilizing custom algorithms or software that are central to the research but not yet described in published literature, software must be made available to editors and reviewers. We strongly encourage code deposition in a community repository (e.g. GitHub). See the Nature Portfolio guidelines for submitting code & software for further information.

Data

Policy information about availability of data

All manuscripts must include a data availability statement. This statement should provide the following information, where applicable:

- Accession codes, unique identifiers, or web links for publicly available datasets
- A description of any restrictions on data availability
- For clinical datasets or third party data, please ensure that the statement adheres to our policy

The codes used to train the Machine learning models can be accessed in the public Gitlab repository. (https://gitlab.com/nicolasevangelou/zeolites_ml.git)

Human research participants

Policy information about studies involving human research participants and Sex and Gender in Research.

Reporting on sex and gender	Not Applicable for our work
Population characteristics	Not Applicable for our work
Recruitment	Not Applicable for our work
Ethics oversight	Not Applicable for our work

Note that full information on the approval of the study protocol must also be provided in the manuscript.

Field-specific reporting

Please select the one below that is the best fit for your research. If you are not sure, read the appropriate sections before making your selection.

☐ Life sciences ☐ Behavioural & social sciences ☒ Ecological, evolutionary & environmental sciences

For a reference copy of the document with all sections, see nature.com/documents/nr-reporting-summary-flat.pdf

Ecological, evolutionary & environmental sciences study design

All studies must disclose on these points even when the disclosure is negative.

Study description	Machine Learning-Assisted Crystal Engineering of a Zeolite
Research sample	Faujasite zeolites, a kind of microporous aluminosilicate materials used for acid catalysis.
Sampling strategy	No sample-size calculation was performed, 174 synthesis experiments were performed step by step, We optimized synthesis conditions step by step, new recipes are developed based on prior recipes. Our trained machine learning models based on collected synthesis data suggested 4 more set of experiment conditions.
Data collection	Zeolite materials used in this work were synthesized by organic template-free methods.
Timing and spatial scale	178 (174+4) synthesis experiments are not collected at the same time. We optimized synthesis conditions step by step, new recipes are developed based on prior recipes. Since each sample was synthesized for 3 ~ 14 days, the whole data collection lasted for 2 ~ 3 years. Samples with similar synthesis conditions showed similar properties, and samples with the same synthesis conditions were repeatable.
Data exclusions	No data were excluded from the analyses.
Reproducibility	174 synthesis experiments were performed, and we have listed their synthesis conditions in details. From the supporting table we provided, it can be shown that samples with similar synthesis conditions showed similar properties, and samples with the same synthesis conditions were repeatable.
Randomization	Randomization was not relevant to our work since our inorganic material synthesis optimization is based on step-by-step research, new recipes are developed based on prior recipes (samples are not independent with each other).
Blinding	Blinding was not relevant to our work since our inorganic material synthesis optimization is based on step-by-step research, new recipes are developed based on prior recipes (samples are not independent with each other).

Did the study involve field work? ☐ Yes ☒ No

Reporting for specific materials, systems and methods

We require information from authors about some types of materials, experimental systems and methods used in many studies. Here, indicate whether each material, system or method listed is relevant to your study. If you are not sure if a list item applies to your research, read the appropriate section before selecting a response.

Materials & experimental systems

n/a	Involved in the study
☒	☐ Antibodies
☒	☐ Eukaryotic cell lines
☒	☐ Palaeontology and archaeology
☒	☐ Animals and other organisms
☒	☐ Clinical data
☒	☐ Dual use research of concern

Methods

n/a	Involved in the study
☒	☐ ChIP-seq
☒	☐ Flow cytometry
☒	☐ MRI-based neuroimaging
